# Polyphenol exposure of mothers and infants assessed by LC–MS/MS based biomonitoring in breast milk

**DOI:** 10.1007/s00216-024-05179-y

**Published:** 2024-02-16

**Authors:** Sabrina Berger, Ian Oesterle, Kolawole I. Ayeni, Chibundu N. Ezekiel, Annette Rompel, Benedikt Warth

**Affiliations:** 1 Department of Food Chemistry and Toxicology, Faculty of Chemistry, University of Vienna, 1090 Vienna, Austria; 2 Universität Wien, Fakultät für Chemie, Institut für Biophysikalische Chemie, 1090 Wien, Austria; 3 Vienna Doctoral School of Chemistry (DoSChem), University of Vienna, 1090 Vienna, Austria; 4 Department of Microbiology, Babcock University, Ilishan-Remo, Ogun State Nigeria; 5 Institute for Bioanalytics and Agro-Metabolomics, Department of Agrobiotechnology (IFA-Tulln), University of Natural Resources and Life Sciences Vienna (BOKU), Konrad-Lorenz Str. 20, 3430 Tulln, Austria; 6 Exposome Austria, Research Infrastructure and National EIRENE Node, Vienna, Austria

**Keywords:** Polyphenols, Breast milk, Tandem mass spectrometry, Human biomonitoring, Exposome research

## Abstract

Exposure to polyphenols is relevant throughout critical windows of infant development, including the breastfeeding phase. However, the quantitative assessment of polyphenols in human breast milk has received limited attention so far, though polyphenols may positively influence infant health. Therefore, a targeted LC–MS/MS assay was developed to investigate 86 analytes representing different polyphenol classes in human breast milk. The sample preparation consisted of liquid extraction, salting out, freeze-out, and a dilution step. Overall, nearly 70% of the chemically diverse polyphenols fulfilled all strict validation criteria for full quantitative assessment. The remaining analytes did not fulfill all criteria at every concentration level, but can still provide useful semi-quantitative insights into nutritional and biomedical research questions. The limits of detection for all analyzed polyphenols were in the range of 0.0041–87 ng*mL^−1^, with a median of 0.17 ng*mL^−1^. Moreover, the mean recovery was determined to be 82% and the mean signal suppression and enhancement effect was 117%. The developed assay was applied in a proof-of-principle study to investigate polyphenols in breast milk samples provided by twelve Nigerian mothers at three distinct time points post-delivery. In total, 50 polyphenol analytes were detected with almost half being phenolic acids. Phase II metabolites, including genistein-7-β-D-glucuronide, genistein-7-sulfate, and daidzein-7-β-D-glucuronide, were also detected in several samples. In conclusion, the developed method was demonstrated to be fit-for-purpose to simultaneously (semi-) quantify a wide variety of polyphenols in breast milk. It also demonstrated that various polyphenols including their biotransformation products were present in breast milk and therefore likely transferred to infants where they might impact microbiome development and infant health.

**Graphical abstract:**

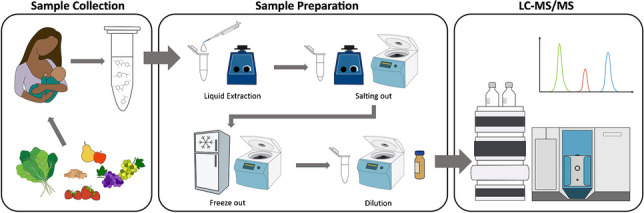

**Supplementary information:**

The online version contains supplementary material available at 10.1007/s00216-024-05179-y.

## Introduction

Polyphenols are secondary plant metabolites that contain a minimum of one aromatic ring substituted with at least one hydroxyl group [1, 2]. In general, they can be split into two major groups, flavonoids and non-flavonoids, that can be further divided into several classes (see Fig. S1). Examples of flavonoids are flavanones, flavones, flavonols, isoflavones, and proanthocyanidins, whereas non-flavonoids contain stilbenes, lignans, and phenolic acids such as hydroxybenzoic acids, hydroxycinnamic acids, and hydroxyphenylacetic acids. As polyphenols typically have functional groups, namely hydroxyl groups, they are frequently and abundantly conjugated by xenobiotic metabolizing enzymes in the human body. The resulting conjugates, mostly glucuronides and sulfates, are commonly found in human biofluids, especially in urine. The biotransformation of xenobiotics, including polyphenols, impacts their chemical properties and bioavailability [3].

Polyphenols are widely studied due to various health benefits, including antibacterial, anti-inflammatory, and antioxidant properties [4–7]. Current research indicates that polyphenols may contribute to a reduced risk of noncommunicable diseases such as cancer [8], cardiovascular disease [9], and neurodegenerative disorders [10, 11]. Besides potentially beneficial effects, polyphenols may also exhibit adverse properties that depend on various factors, e.g., dosage and environmental interactions [12]. These adverse human effects of polyphenols include reducing iron absorption [13–15], interactions with drugs and other xenobiotics [16–19], inhibiting of digestive enzymes [12], and affecting the hormonal balance [20, 21]. For example, combinatory effects between polyphenols and mycotoxins may contribute to increased estrogenic effects of both the polyphenols and the mycotoxins [22]. In addition to their bioactive properties, polyphenols are of great interest as they are a class of molecules prevalent in numerous plant-based foods including fruits, vegetables, grains, tea, cocoa, and coffee [23–25].

Due to the health-promoting effects of polyphenols and their prevalence in foodstuff, significant research interest exists in evaluating uptake, distribution, metabolism, and excretion of polyphenols in humans. In particular, the question arises if polyphenols are present in breast milk and follow lactational transfer to infants, and if so, whether they have a positive or negative influence on infant health, such as aiding in preventing the development of chronic diseases [26] or modulating microbiome development [27]. In general, breast milk is considered the ideal food for infants. The World Health Organization (WHO) recommends exclusively breastfeeding infants for the first 6 months of life and to continue breastfeeding following the introduction of complementary foods for up to 2 years or longer [28, 29]. To determine the potential impact of polyphenols on infant development and health during this critical window of susceptibility, reliable quantification in breast milk is needed. This would allow investigation of the transfer and biotransformation of ingested polyphenols from the diet of the mothers to their breast milk and subsequently their infants. Moreover, this information would yield new insights to pediatricians and mothers, potentially allowing a tailored adjustment of their diet to positively impact their infant’s health. For example, it could be investigated if the consumption of a polyphenol-rich diet may be an alternative to antibiotics for either treating or preventing (mild) urinary tract infections in susceptible neonates and infants.

Since polyphenols are an extensive family of diverse molecules containing many different classes, it is advantageous to quantify individual polyphenols rather than simply the total polyphenol content [30]. Therefore, a suitable sample preparation approach and a sensitive and specific analytical method are required for the comprehensive quantification of polyphenols. An essential technique in modern human biomonitoring is liquid chromatography coupled to triple quadrupole mass spectrometry (LC–MS/MS) using multiple reaction monitoring (MRM) mode [31]. Targeted LC–MS/MS allows to selectively detect and quantify specific analytes with a high sensitivity, specificity, and accuracy.

Therefore, the aim of this study was to develop and in-house validate a targeted LC–MS/MS method to quantify a comprehensive selection of analytes representing all main polyphenol classes in human breast milk. This involved transferring a previously published method for polyphenols in other human specimens (urine, serum, and plasma) [32] to the highly complex breast milk matrix. A sample preparation method was developed to extract 86 polyphenols representing 15 different chemical classes for broad coverage. After optimization, the method was validated and applied in a pilot study to prove its suitability and fit-for-purpose.

## Materials and methods

### Chemicals, reagents, and solvents

Information on the reference standards, reagents, and solvents used during method development, validation, and pilot study are available in Table S1 in the supplementary information (SI). Single standard stock solutions were prepared by dissolving the solid polyphenol standards in methanol (MeOH), as described by Oesterle et al. [32]. For optimization of the sample preparation and the method validation, individual stock solutions were mixed at different concentrations and diluted with MeOH to prepare multiple working solutions with concentrations between 0.2 and 130,000 ng*mL^−1^. All working and individual standard solutions were stored at − 20 °C.

### Sample preparation

As breast milk is a highly complex biological matrix, different sample preparation approaches were tested and optimized, including solid phase extraction (SPE) with *Waters* Oasis cartridges. The final optimized sample preparation protocol was established as follows: to an aliquot of 200 µL of human breast milk, 400 µL of acetonitrile (ACN) acidified with 1% v/v formic acid (FA) was added and thoroughly vortexed for 3 min. Subsequently, 80 mg anhydrous magnesium sulfate and 20 mg sodium chloride were added, and the sample was again vortexed for 3 min. The sample was then centrifuged for 10 min (2000 × g, 4 °C), and the supernatant was chilled for 2 h at − 20 °C. Following the freeze-out step, the sample was centrifuged for 2 min (18,000 × g, 4 °C) and the supernatant diluted 1:1 with acidified water (1% v/v FA). The sample was then centrifuged for 5 min (18,000 × g, 4 °C) and the supernatant was transferred to an amber LC glass vial. Enzymatic deconjugation was not performed as several conjugated reference standards were included in the method for direct determination and because deconjugation enzymes are typically contaminated with a high number of xenobiotics, especially polyphenols [33].

### LC–MS/MS instrumentation

The UHPLC-ESI-QTrap-MS/MS system used was composed of a 1290 Infinity II LC (*Agilent*) connected to a QTrap 7500 MS (*Sciex*), equipped with a heated electrospray ionization source (ESI). Data was acquired in scheduled multiple reaction monitoring (sMRM) mode using fast polarity switching. An optimized LC–MS/MS method that was previously developed for the measurement of polyphenols in other complex biological matrices, i.e., urine, serum, and plasma [32], was transferred from a QTrap 6500^+^ to a QTrap 7500 system and used as the basis for the breast milk assay described here. The majority of the LC and MS parameters remained the same; however, some parameters, such as retention times, retention time windows, and declustering potential, were adjusted accordingly (Table S2). A VanGuard precolumn (1.8 μm, *Waters*) attached to an Acquity UPLC HSS T3 column (1.8 μm, 2.1 × 100 mm, *Waters*) was used to achieve chromatographic separation. The temperature of the column compartment was set to 30 °C and of the autosampler to 7 °C. The mobile phases used were 0.1% v/v FA in H_2_O (eluent A) and 0.1% v/v FA in ACN (eluent B). The injection volume was 3 µL and the flow rate was set to 0.6 mL*min^−1^. The gradient (Table S3) started with 5% eluent B and was held for 2 min. Afterwards, eluent B was raised linearly to 64% within 10 min and then increased to 95% for a 2 min hold. Eluent B was then immediately decreased to 5% for a final 2 min re-equilibration step. The following ESI parameters were used: curtain gas 35 arb, sheath gas 90 arb, drying gas 90 arb, collision gas set to medium, source temperature 550 °C, and entrance potential at 10 V in positive and − 10 V in negative mode. The voltage of the ion capillary was set to 5500 V in positive and − 4500 V in negative mode.

### Validation experiments

The method was validated in-house following the guidelines set by Eurachem [34] and the EU Commission decision 2002/657/EC [35]. Analytical figures of merit including selectivity, repeatability (RSD_r_), intermediate precision (RSD_R_), regression coefficient (*R*^2^), recovery (*R*_E_), and signal suppression or enhancement (SSE) were evaluated at three concentration levels.

Due to a lack of matrix-matched reference material, multiple breast milk samples were pooled and used as “blank” breast milk for spiking experiments and for the matrix-matched calibration curves [36]. For spiking and creation of the calibration curves, a multi-standard working solution was prepared from the individual polyphenol stock solutions. This working solution was then serially diluted to create five additional multi-standard working solutions. With these six working solutions, a six-point neat solvent (ACN:H_2_O:FA, 49.5:49.5:1) and a matrix-matched calibration curve (calibration ranges are reported in Table 1) were prepared. During method optimization, a multi-standard solution was measured to estimate the LOQs of the analytes. Based on these values, the calibration points for each analyte were set as 0.33, 1, 3, 10, 30, and 100 times their respective estimated LOQ. Matrix-matched samples were spiked at three different concentration levels: low, middle, and high (Table S4) before the sample preparation procedure (pre-spiked samples). For each validation experiment, triplicates of the pre-spiked samples were prepared at each spiking level. Overall, three individual validation experiments were performed over the course of 3 months, and one of the validation experiments included two additional re-measurements of the acquisition sequence on the same day to determine the intraday stability (RSD_r_) of the method. To ensure the selectivity of the method, solvent and matrix-matched blanks and spiked samples were examined for any potential interfering signals throughout the validation procedure.

**Table 1:** Range of the calibration curve, regression coefficient (*R*^2^), signal suppression and enhancement (SSE), limit of detection (LOD), limit of quantification (LOQ), and the mean recovery (*R*_E_) of the three spiking levels for each analyte as evaluated during in-house validation. Parameters that could not be determined are listed as n.d

Analyte	CAS number	Calibration range (ng*mL^−1^)	*R*^2^	SSE (%)	LOD (ng*mL^−1^)	LOQ (ng*mL^−1^)	*R*_E_ (%)
***Dihydrochalcones***							
Phloretin	60–82-2	0.015–7.2	0.991	114	0.017	0.034	96
***Hydroxybenzoic acids***							
3,5-Dihydroxybenzoic acid	99–10-5	0.22–29^a^	0.903	120	0.41	0.82	44
3-Hydroxybenzoic acid	99–06-9	1.2–590	0.989	109	0.84	1.7	96
4-Hydroxybenzoic acid	99–96-7	0.08–35	0.988	109	0.19	0.38	88
Benzoic acid	65–85-0	3.5–1600	0.976	107	46^b^	92^b^	84
Ellagic acid	476–66-4	1.74–78^c^	0.991	163	8.9^b^	18^b^	8
Ethyl gallate	831–61-8	0.004–2.4	0.992	113	0.0024	0.0048	88
Gallic acid	149–91-7	0.023–3.0^a^	0.905	129	0.028	0.056	33
Protocatechuic acid	99–50-3	0.015–6.6	0.873	112	0.059	0.12	42
Salicylic acid	69–72-7	0.2–27^a^	0.994	124	0.48	0.96	86
Syringic acid	530–57-4	0.022–11	0.992	112	0.068	0.14	95
Vanillic acid	121–34-6	0.16–70	0.988	111	0.17	0.33	94
***Hydroxycinnamic acids***							
Caffeic acid	501–16-6	0.3–130	0.993	110	0.55	1.1	88
Caffeic acid-3-β-D-glucuronide	1093679–73-2	0.014–6.8	0.991	107	0.0085	0.017	69
Chlorogenic acid	327–97-9	0.29–38^a^	0.996	112	0.32	0.65	60
Cinnamic acid	621–82-9	1.5–650	0.994	113	2.1	4.3	90
Dihydrocaffeic acid	1078–61-1	0.082–36	0.996	110	0.16	0.33	88
Dihydroferulic acid	1135–23-5	0.11–49	0.989	114	0.35	0.71	100
Ferulic acid/Isoferulic acid	537–98-4/537–76-5	0.058–26	0.994	110	0.096	0.19	89
p-Coumaric acid	501–98-4	0.044–19	0.990	109	0.046	0.092	94
Sinapic acid	530–59-6	0.022–11	0.993	110	0.087	0.17	96
trans-m-Coumaric acid	588–30-7	0.25–110	0.992	107	0.69	1.4	86
trans-o-Coumaric acid	583–17-5	0.13–64	0.994	107	0.2	0.4	94
***Hydroxyphenylacetic acids***							
3-(3-Hydroxyphenyl)propionic acid	621–54-5	0.09–37	0.990	108	0.38	0.76	95
3-Hydroxyphenylacetic acid	621–37-4	2–890	0.990	107	4.5	9	98
4-Hydroxyphenylacetic acid	156–38-7	2.6–1100	0.991	104	5.9	12	94
Homoprotocatechuic acid	102–32-9	0.4–182	0.992	107	1.7	3.4	93
Homovanillic acid	306–08-1	0.73–331	0.991	109	0.64	1.3	97
***Lignans***							
Enterodiol	80226–00-2	0.005–2.1	0.987	109	0.017	0.034	88
Enterolactone	78473–71-9	0.014–6.8	0.992	117	0.018	0.036	89
***Others***							
2,6-Dimethoxyphenol	91–10-1	0.05–21	0.986	106	0.061	0.12	98
3,5-Dimethoxy-4-hydroxyphenylacetic acid	4385–56-2	0.31–140	0.993	109	0.43	0.86	97
3-Methylcatechol	488–17-5	0.15–67	0.992	109	0.16	0.32	94
4-Methylcatechol	452–86-8	0.33–150	0.993	110	0.24	0.48	94
Catechol	120–80-9	3.3–130^a^	0.759	110	5	10	57
Eugenol	97–53-0	2.2–990	0.985	103	4.7	9.4	91
Hydroxytyrosol	90–05-1	0.041–19	0.992	112	0.034	0.068	90
Pyrogallol	10597–60-1	0.42–190	0.985	127	1.4	2.8	91
Thymol	89–83-8	0.67–300	0.937	99	69^b^	140^b^	76
Urolithin A	1143–70-0	0.007–3.6	0.992	111	0.014	0.028	90
***Stilbenes***							
Dihydroresveratrol	58,436–28-5	0.035–16	0.991	109	0.054	0.11	91
Polydatin	65914–17-2	0.012–5	0.988	106	0.061	0.12	99
Pterostilbene	537–42-8	0.016–7	0.994	121	0.034	0.068	94
Resveratrol	501–36-0	0.03–14	0.992	113	0.043	0.086	89
***Anthocyanins***							
Cyanidin	87725–42-6	4.4–2000^a^	0.975	115	39^b^	78^b^	46
Cyanidin-3-O-glucoside	47705–70-4	0.06–8.1^a^	0.995	140	0.18^b^	0.36^b^	15
Cyanidin-3-O-rutinoside	28338–59-2	0.052–6.9^a^	0.995	136	0.26^b^	0.52^b^	21
Cyanidin-3-O-sambubioside	63535–17-1	0.1–15^a^	0.996	138	0.29^b^	0.58^b^	7
Delphinidin	528–53-0	5.3–703^a^	0.959	178	28^b^	56^b^	23
Delphinidin-3-O-glucoside	50986–17-9	2.1–300^a^	0.981	160	11^b^	22^b^	10
***Catechins***							
(-)-Epicatechin	490–46-0	0.2–90	0.992	107	0.24	0.48	90
(-)-Epicatechin gallate	1257–08-5	0.08–36	0.989	117	0.13	0.26	74
(-)-Epigallocatechin	970–74-1	1.4–620	0.968	123	5.3	11	92
(-)-Epigallocatechin gallate	989–51-5	1–440	0.970	135	4.2	8.4	74
(-)-Gallocatechin	3371–27-5	1.4–620	0.983	250	4.9	9.8	86
( +)-Catechin	154–23-4	0.12–53	0.993	113	0.17	0.34	83
***Flavanones***							
(+/-)-Naringenin	153–18-4	0.008–1.1^a^	0.992	121	0.036	0.072	86
8-Prenylnaringenin	53846–50-7	0.02–8.8	0.992	114	0.016	0.032	93
Hesperetin	520–33-2	0.009–3.8	0.993	114	0.013	0.026	96
Hesperidin	520–26-3	0.006–2.6	0.991	120	0.0094	0.019	93
Isoxanthohumol	521–48-2	0.004–1.8	0.994	115	0.0054	0.011	93
Naringin	10236–47-2	0.23–100	0.989	112	0.56	1.1	84
Neohesperidin	13241–33-3	0.3–140	0.992	110	0.6	1.2	85
Neohesperidin dihydrochalcone	20702–77-6	0.006–2.6	0.994	109	0.0085	0.017	85
Xanthohumol	6754–58-1	0.012–5.3	0.992	109	0.017	0.034	91
***Flavones***							
Apigenin	520–36-5	0.009–3.9	0.988	117	0.0047	0.0094	89
Diosmetin	520–34-3	0.005–2.9	0.990	112	0.015	0.03	93
Diosmin	520–27-4	0.024–11	0.982	119	0.069	0.14	100
***Flavonols***							
( +)-Rutin	480–41-1	0.03–13	0.988	106	0.031	0.062	67
Isorhamnetin	480–19-3	0.006–2.6	0.993	119	0.0089	0.018	79
Kaempferol	520–18-3	0.12–60	0.990	121	0.17	0.34	90
Kaempferol-3-O-glucuronide	22688–78-4	0.008–3.5	0.992	111	0.013	0.026	87
Quercetin	117–39-5	0.052–7.1^a^	0.991	129	0.12	0.24	63
Quercetin-7-O-β-D-glucuronide	38934–20-2	0.031–14	0.989	121	0.066	0.13	62
***Isoflavones***							
Biochanin A	491–80-5	0.009–3.9	0.994	117	0.014	0.028	90
Daidzein	486–66-8	0.01–4.8	0.992	109	0.034	0.068	89
Daidzein-7-β-D-glucuronide	38482–80-3	0.032–14	0.993	107	0.063	0.13	88
Genistein	446–72-0	0.01–4.3	0.977	119	0.0047	0.0094	79
Genistein-7-β-D-glucuronide	38482–81-4	0.04–16	0.993	108	0.054	0.11	92
Genistein-7-sulfate	182322–62-9	0.27–13^d^	0.930	105	0.18	0.36	59
S-Equol	531–95-3	0.42–190	0.986	106	2.2	4.4	88
***Proanthocyanidins***							
Procyanidin A2	41743–41-3	0.07–31	0.983	108	0.24	0.48	82
Procyanidin B1	20315–25-7	1.1–500	0.994	106	1.1	2.2	67
Procyanidin B2	29106–49-8	0.3–130	0.950	108	16^b^	33^b^	69
Procyanidin C1	37064–30-5	0.21–93	0.986	113	0.62	1.2	60

^a^The maximum concentrations of the calibration curve exceeded the range of linearity, thus the highest calibration point was excluded

^b^No chromatographic peak at the lowest spiking level thus, the standard deviation of the next highest spiking level with a chromatographic peak was used to calculate LOD and LOQ

^c^The two highest concentrations of the calibration curve exceeded the range of linearity, thus they were excluded

^d^The concentrations of the calibration curve were chosen too high, and the limit of linearity was reached. Therefore, the three highest calibration points were excluded

The recovery was calculated by dividing the measured concentration of the pre-spiked samples by the theoretical concentration spiked at each of the three different levels. For each spiking level, the overall recovery was calculated as the mean of all measurements (*n* = 9). Limit of detection (LOD) was evaluated by dividing the standard deviation of the measured concentration of the pre-spiked samples (low level) by the square root of the number of replicates of all measurements (*n* = 9) and multiplying it by three. The limit of quantification (LOQ) was defined as two times the LOD. Intermediate precision and repeatability were evaluated at each spiking level. The intermediate precision was defined as the relative standard deviation of the measured concentration of the nine pre-spiked samples from the three separate validation experiments, measured on different days. Intraday repeatability was defined as the relative standard deviation of the measured concentration of the nine pre-spiked samples from the validation experiment that was measured three times on the same day. The regression coefficient from each matrix-matched calibration curve was calculated. Signal suppression and enhancement (SSE) effect was calculated by dividing the slope of the matrix-matched calibration curve by the slope of the solvent calibration curve and expressed as percentage. Therefore, a SSE value below 100% indicates signal suppression, while a SSE value greater than 100% indicates signal enhancement [37]. The mean of the regression coefficients and the signal suppression and enhancement effect over the three validation experiments were calculated and reported. Ensuring the evaluation of these validation figures of merit for each analyte, the following criteria for validation requirements were used: a recovery between 50 and 120%, a regression coefficient of at least 0.95, and repeatability and intermediate precision below 45%, 30%, and 25% for low, middle, and high spiking levels, respectively. The repeatability and intermediate precision criteria were determined with the Horwitz equation [34].

Data analysis, peak integration, and concentration calculations were evaluated with *SCIEX OS (v3.0)*. All chromatographic peaks were smoothed with a low-grade filter. A 1/x weighting was applied to all calibration curves. Standard addition was applied to the calibration curves of analytes in which a signal was detected in the non-spiked matrix-matched samples. Calculations of the standard addition and the other validation figures of merit were performed in *Excel 16.0*.

### Biological samples

The pooled breast milk used for method development and validation was kindly provided by the Semmelweis Women’s Clinic in Vienna [36, 38]. The proof-of-principle experiments included aliquots of breast milk samples from a previous study conducted by Ayeni et al. [39] that explored mycotoxin exposure patterns in different biological matrices and a potential impact on gut microbiome development. Details of sample collection are reported in Ayeni et al. [39]. In brief, breast milk samples were collected from twelve Nigerian mothers from Ilishan-Remo, Ogun state. The mothers’ age ranged between 25 and 40 years, and their diet consisted of various cereal-based foods (e.g., bread, rice, ogi), tubers (yam, cassava), legumes (e.g., beans), vegetables (e.g., okra, onion), fruits (e.g., tomatoes, oranges, apples, bananas), fish, and meat. The breast milk was expressed manually by the mothers and stored in a fridge overnight until they were collected by trained study personnel and stored at − 20 °C. The samples were transported on dry ice to the laboratory in Vienna for mass spectrometric analysis. Ethical approval was obtained from the Ethical Committee of Babcock University (BUHREC421/21R, BUHREC466/23). Prior to their inclusion in the studies, all mothers were informed and provided written consent.

For the positive identification of the polyphenol analytes in the biological samples, stringent criteria were defined. Analytes with a retention time deviation greater than 0.05 min compared to their respective matrix-matched calibration curve were excluded. Additionally, only analytes that had both the quantifier and qualifier ions present, with an ion ratio deviation of less than 20% compared to their respective matrix-matched calibration curve, were considered. For analytes that showed a chromatographic signal near the LOD, an ion ratio deviation of up to 50% was considered acceptable, since the background noise has a strong influence on the ion ratios at these low concentrations. For all positively identified analytes, the concentration was determined using the matrix-matched calibration curve and corrected with the recoveries calculated during method validation.

## Results and discussion

### Method optimization

Extracting a wide range of analytes from a complex biological matrix such as breast milk is a challenging task. In several studies, a QuEChERS approach (quick, easy, cheap, effective, rugged, and safe) has been successfully utilized to extract analytes such as pesticides [38, 40, 41] and other xenobiotics [42–44] from foods with a high lipid content. Few studies [45, 46] investigated the quantification of polyphenols in breast milk, but these did not include as many analytes from multiple polyphenol classes. Moreover, the method presented here includes phase II metabolites of polyphenols such as sulfates and glucuronides, whereas previous studies from Song et al. [45] and Lu et al. [46] used *β*-glucuronidase/sulfatase treatment to deconjugate potential phase II metabolites. As a starting point, a method established for quantifying mycotoxins in breast milk was selected [36, 47]. This method combined a QuEChERS approach with a freeze-out step, a SPE cleanup, and an evaporation step. Here, in the first step, the procedure was scaled down in order to use a reduced volume of breast milk (200 µL instead of 1 mL) and improve the high-throughput feasibility. However, the results showed low recoveries and severe matrix interferences. Therefore, various extraction solvents, including ACN, MeOH, and hexane, both pure and acidified with up to 3% v/v FA, were tested at different extraction ratios (solvent to breast milk), e.g., 1:1 v/v, 2:1 v/v, and 3:1 v/v, for lipid removal. Different approaches were also tested to optimize the SPE step, including acidifying the ACN with up to 3% FA used to elute the analytes from the C18 SPE cartridges (Oasis HLB Prime, 1 cc, 30 mg, *Waters*). Protocols with and without the SPE step as well as procedures with and without the drying step with a vacuum concentrator were additionally tested.

The final, optimized sample preparation procedure, which yielded the overall best analyte recoveries with the least signal suppression/enhancement effects, is described in “Sample preparation.” In brief, the procedure contained a liquid extraction step with acidified ACN, a salting-out step with anhydrous magnesium sulfate and sodium chloride, a freeze-out step, and finally a dilution step with acidified H_2_O.

### Validation experiments

Overall, the in-house validation was successful with 59 out of 86 (69%) of the polyphenol analytes fulfilling all validation criteria at all three spiking levels. An additional ten polyphenols (11%) passed all validation criteria for the medium and high spiking levels. A summary of the validation results is listed in Table 1 and the detailed results are reported in Tables S4–S5. In comprehensive multi-analyte human biomonitoring assays, pragmatic compromises are essential to keep a fine balance between covering as many analytes as possible while ensuring high sensitivity and minimal matrix interferences [48]. Consequently, it was not expected that all 86 analytes will perform ideally applying this method. For the polyphenols that did not fulfill all the strict validation figures of merit, semi-quantification is still possible and can be helpful in comprehensive exposome studies as well as for answering biological and nutrition-related questions. The selectivity of the method was evaluated by comparing the matrix-matched samples enriched with standards to the matrix-matched “blank” and solvent samples enriched with standards. No interferences were detected for the majority of the analytes. Due to a lack of available reference material, the biological matrix used was not a true “blank”, thus, several analytes, e.g., (+/-)-naringenin, had a chromatographic peak present in the matrix-matched “blank” (Table S4). Consequently, standard addition was applied for these analytes. Moreover, despite having individual standards for the isomers ferulic acid and isoferulic acid, these two analytes co-eluted and were acquired as a sum parameter because the same MRM transitions were observed during MS parameter optimization.

The recovery, intermediate precisions, and repeatability of the method are listed in Table S4, with the mean recoveries also reported in Table 1. For 70% of all analytes, the mean recoveries, calculated from the three spiking levels of each analyte, were in the range of 80–120%. The intermediate precision of the low, middle, and high spiking levels was in the ranges of 5–61%, 4–56%, and 7–62%, respectively, and the repeatability for the three spiking levels was in the ranges of 4–87%, 3–59%, and 2–71%, respectively. These results demonstrate the overall stability of the workflow for most analytes, both intraday and interday when taking into account that the higher values were typically derived from very few analytes for which full quantitative assessment was not intended by design. The LOD and LOQ values, calibration range, regression coefficient, and SSE are reported in Table 1. The linear calibration curves of each analyte from one validation sequence are depicted in Table S7. It was observed that the regression coefficients for all analytes were between 0.76 and 0.996, with a median *R*^2^ of 0.991. Moreover, 93% of all analytes had a regression coefficient greater than 0.95. For some analytes, the maximum concentrations chosen for calibration were too high and exceeded the linear range of the detector; thus, the highest points of the calibrations were removed (Table S4). As expected, the limits of detection varied greatly between the different analytes and the polyphenol classes. The LODs for all analytes ranged between 0.0041 and 87 ng*mL^−1^, with a median LOD of 0.17 ng*mL^−1^. Many of the included polyphenol classes showed very low LODs, such as flavanones, flavonols, hydroxycinnamic acids, isoflavones, and stilbenes with LODs ranging from 0.0069 to 0.48 ng*mL^−1^, 0.015–0.15 ng*mL^−1^, 0.014–2.5 ng*mL^−1^, 0.0041–1.9 ng*mL^−1^, and 0.039–0.069 ng*mL^−1^, respectively. The SSE was evaluated throughout the validation procedure by comparing the slope of the matrix-matched calibration curve with that of the solvent calibration curve. The SSE was calculated in a manner that a value of 100% indicates that there is no effect of the biological matrix on the ionization efficiency, while a value above 100% would indicate an enhanced signal and a value below 100% that the signal is decreased. Overall, the SSE for all the analytes was in the range of 99% (thymol) to 250% ((-)-gallocatechin). Furthermore, 91% of all analytes had a SSE between 99 and 130%. The two polyphenol classes that showed the highest average SSE were anthocyanins and catechins which were 145% and 141%, respectively. The signal enhancement of these two classes may be attributed to their structure, as e.g. anthocyanins have a positive charge unlike other polyphenol classes. Although breast milk is an extremely complex matrix, the optimized sample preparation resulted in minimal SSE, a high sensitivity, and decent recoveries for most analytes.

**Fig. 1:**
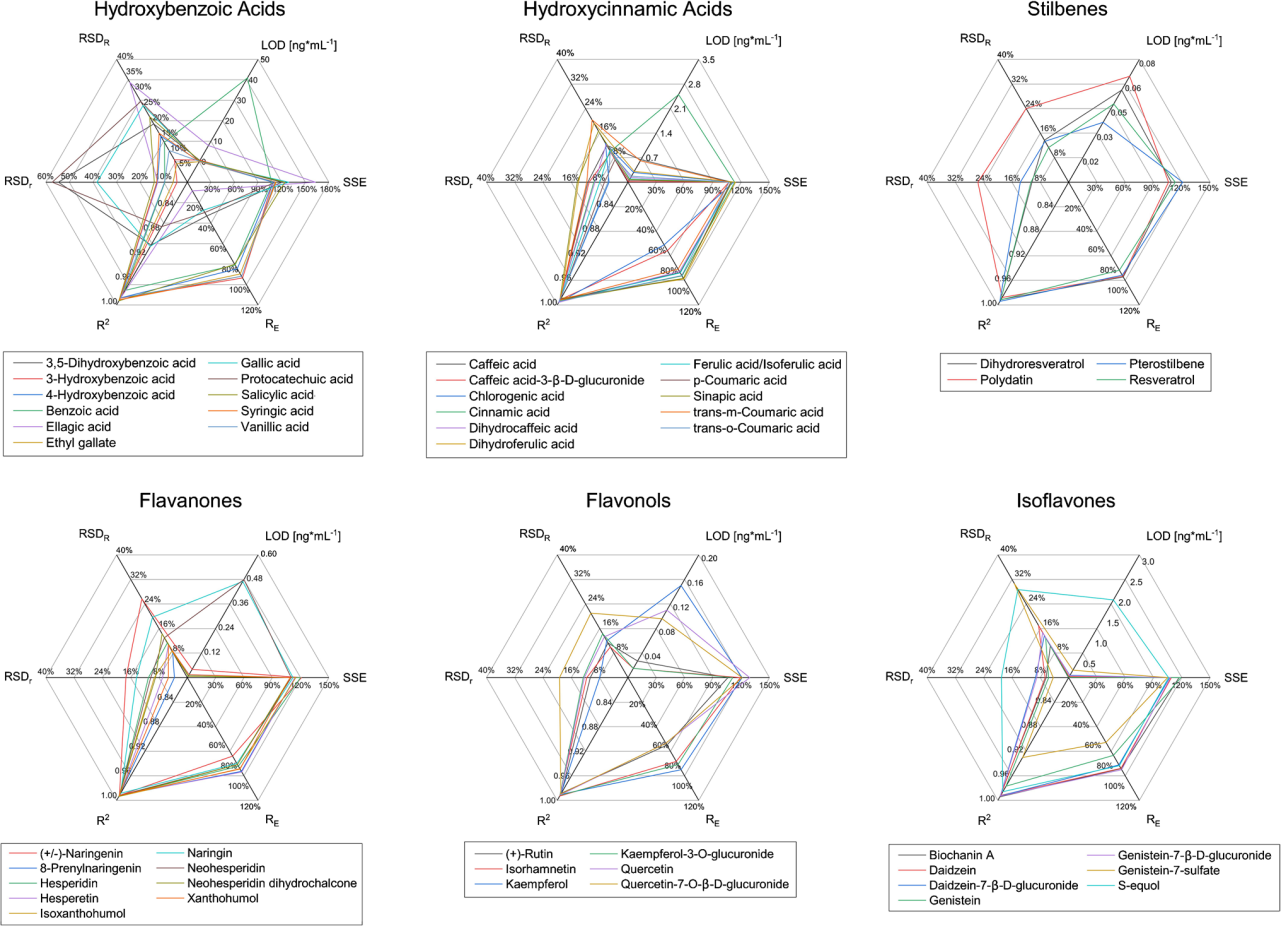
Analytical figures of merit evaluated during method validation for six selected polyphenol classes (three flavonoid and three non-flavonoid classes). Detailed results for all analytes are reported in Table 1, S4, and S5. The recovery (*R*_E_), intermediate precision (RSD_R_), and repeatability (RSD_r_) are displayed as the mean of the three spiking levels (low, middle, high). The limit of detection (LOD), calculated from the standard deviation of the lowest spiking level, and signal suppression and enhancement effect (SSE), calculated from the slopes of the calibration curves, are also displayed. For graphical representations of the remaining polyphenol classes, the interested reader is referred to the SI (Fig. S2)

Since the presented assay is a comprehensive multi-analyte method, it was expected that some polyphenol classes performed better than others based on the accepted compromises during sample preparation, chromatographic separation, and mass spectrometric detection. However, the classes without superb performance were not excluded, to give a more holistic overview. The overall validation results and specific figures of merit that did not meet the validation criteria are shown in Table S5. For example, the anthocyanins did not fulfill all validation criteria. This could be attributed to their structure with a positive charge, which makes anthocyanins more polar than other polyphenols. Therefore, during sample preparation, anthocyanins may remain in the aqueous phase during the liquid–liquid extraction step with an organic solvent, leading to their lower recoveries. Moreover, carry-over was observed for anthocyanins in the LC–MS/MS method; thus, for a successful validation, different or more acidic chromatographic conditions would be needed [49, 50]. Also, several catechins, proanthocyanidins, and hydroxybenzoic acids were not successfully validated as some of these analytes showed carry-over. In addition, for the two hydroxybenzoic acids, benzoic acid and ellagic acid, only one MRM transition was available. On the contrary, for dihydrochalcones, flavanones, flavones, flavonols, hydroxycinnamic acids, isoflavones, lignans, and stilbenes, more than 70% of the included analytes fulfilled all stringent validation criteria. The analytical figures of merit evaluated during the method validation for all analytes, separated by polyphenol class, are displayed in Fig. 1 and Fig. S2. It can be observed that polyphenols from the same chemical class typically behave in a similar manner, as they show comparable recoveries, SSEs, intermediate precisions, and repeatability.

Comparing this novel workflow with previously published methods is challenging as only a limited number of methods have been published that were designed specifically for polyphenols in human breast milk. Many biomonitoring methods investigating xenobiotics in breast milk focused on toxicants, including mycotoxins [36, 47, 51], heavy metals [52, 53], persistent organic pollutants [54, 55], volatile organic compounds [56], phthalates [57], and perfluorinated compounds [58], to study their transfer and potential adverse health impact on infants. The methods that quantify polyphenols in breast milk commonly focus on a fraction of the number of analytes that were included in the method developed here and do not comprehensively investigate all the main polyphenol classes [38, 45, 46, 59]. A method published by Song et al. [45] measured eight flavonoids and several carotenoids in breast milk and reported LODs that were higher than those established in the present study for the majority of the analytes common between both methods. For example, the LODs determined for epicatechin gallate, hesperetin, and quercetin (2.7 ng*mL^−1^, 6.7 ng*mL^−1^, and 2.5 ng*mL^−1^, respectively) were approximately 21, 516, and 21 times, respectively, higher than the LODs determined herein. The next-generation biomonitoring method developed by Jamnik et al. [38] for a wide range of xenobiotics in different biofluids showed LODs that were overall in a similar range as reported here for breast milk, e.g., for the analytes 8-prenylnaringenin, isoxanthohumol, and resveratrol, Jamnik et al. [38] reported LODs of 0.0075 ng*mL^−1^, 0.0048 ng*mL^−1^, and 0.15 ng*mL^−1^, respectively, whereas the LODs reported here were at 0.016 ng*mL^−1^, 0.0054 ng*mL^−1^, and 0.043 ng*mL^−1^ respectively. However, unlike in this work, the included polyphenols, 8-prenylnaringenin, daidzein, enterodiol, enterolactone, genistein, isoxanthohumol, resveratrol, and xanthumol did not fulfill their defined validation criteria. Finally, Lu et al. [46] analyzed twelve polyphenols (six flavonoids and six non-flavonoids) in breast milk. Lu et al. [46] reported mainly higher LODs than the values achieved with the method presented here. For instance, their reported LODs for kampferol, quercetin, and daidzein (2.2 ng*mL^−1^, 1.2 ng*mL^−1^, and 0.5 ng*mL^−1^, respectively) were approximately 15, 11, and 19 times, respectively, higher than the LODs determined with the workflow presented here. Considering the large quantity of positively validated analytes and their relatively low LODs, it can be concluded that, despite its broad chemical coverage and the quite generic sample preparation, the method performs favorably.

### Application of the developed method to human breast milk samples

To show its applicability in real-life samples, the validated method was applied in a pilot study to comprehensively assess the polyphenol profiles in 30 breast milk samples from twelve Nigerian mothers obtained at months one, six, and twelve post-delivery. Since some mothers dropped out of the study, and others did not breastfeed until the twelfth month, not all samples were available for every time point.

**Fig. 2:**
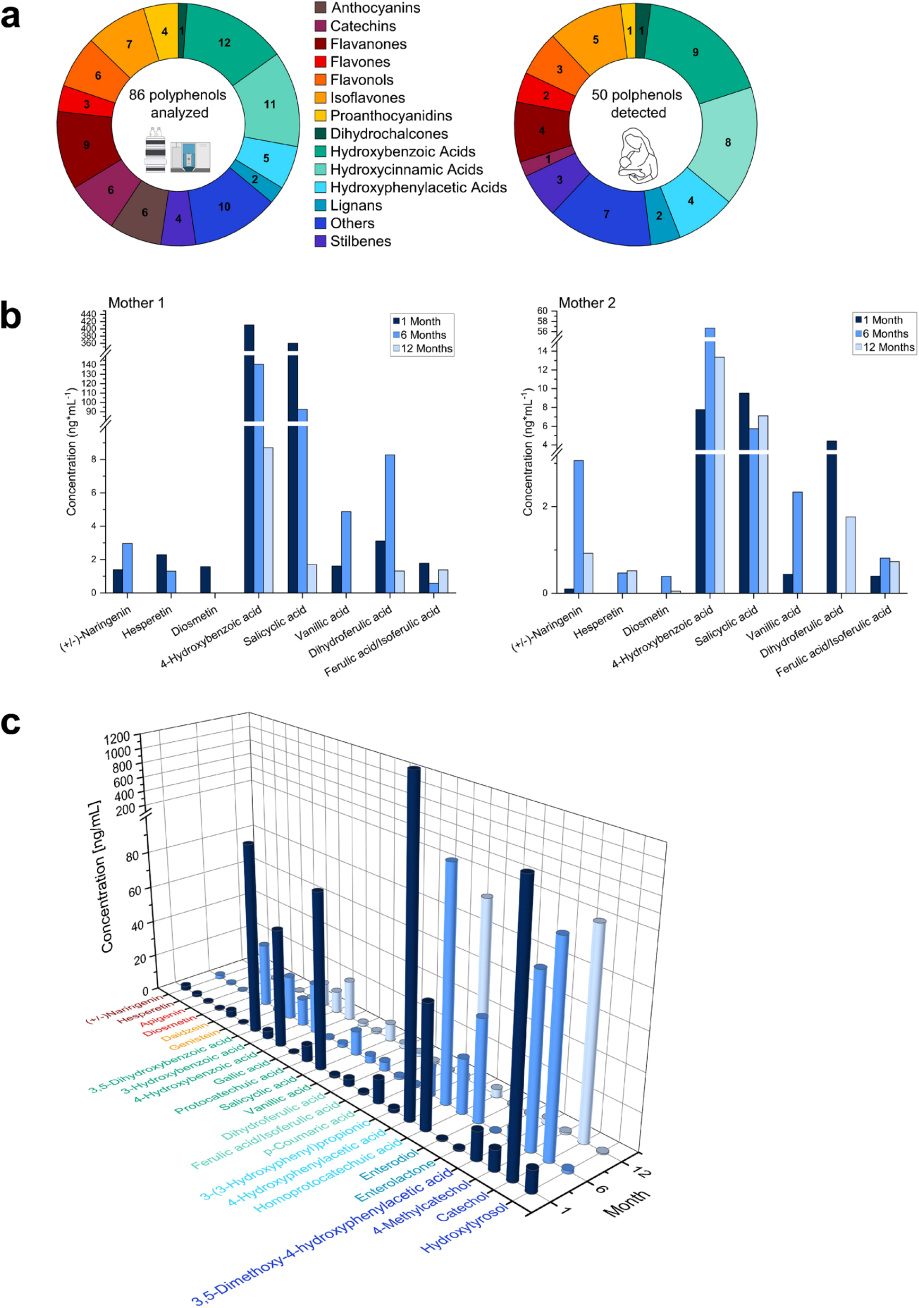
** a** Pie charts showing the number of polyphenol analytes included in the method (left) and the number of polyphenol analytes detected in the pilot study (right) separated by their polyphenol class. **b** Boxplots of the concentrations for selected analytes at the three different sampling time points for two Nigerian mothers. Only analytes detected with concentrations over the LOQ are displayed. **c** A 3D boxplot of the average concentration between the twelve mothers for each analyte detected, separated by time points. Only the analytes that were detected at least once per time point and had a concentration over the LOQ are shown

From the 86 polyphenol analytes included in the method, a total of 50 polyphenols, including some metabolic products, were identified in the breast milk samples (Fig. 2a, Table 2). The majority of the detected polyphenols were phenolic acids, a class that includes numerous biotransformation products of larger polyphenols, such as proanthocyanidins [60–62]. Several analytes were detected in a high number of the samples including salicylic acid (found in all 30 samples), an abundant plant metabolite, (+/-)-naringenin (27 samples), a biomarker for citrus fruit consumption [63, 64], and protocatechuic acid (17 samples), a hydroxybenzoic acid present in many vegetables and fruits, and one of the main metabolites of anthocyanins and procyanidins [65, 66]. The polyphenol contents in breast milk can be significantly influenced by several factors. These include dietary habits and the metabolism of the mothers, as well as the polyphenol content of the consumed food, which can be influenced by geographic location and climatic conditions [67]. Examples of chromatographic peaks for polyphenols identified in the pilot study for selected analytes are illustrated in Fig. 2d and e. The quantification of polyphenols present in breast milk provides only a brief insight on the breast milks’ current composition, and it is difficult to compare between different mothers and time points, especially as the sample size is relatively small.

**Table 2:** Minimum (min), maximum (max), and mean concentration^e^ of the 50 detected polyphenols in the pilot study of breast milk samples from Nigerian mothers. In addition, the number of samples (*n*) in which the analyte was positively detected, out of 30 total samples, is listed. The limit of quantification (LOQ) for each detected polyphenol is also given

Analyte	LOQ (ng*mL^−1^)	Min (ng*mL^−1^)	Max (ng*mL^−1^)	Mean ± standard deviation (ng*mL^−1^)	*n*
***Dihydrochalcones***					
Phloretin	0.034	< LOQ	< LOQ	-	2
***Hydroxybenzoic acids***					
3,5-Dihydroxybenzoic acid	0.82	< LOQ	400	49 ± 110	16
3-Hydroxybenzoic acid	1.7	< LOQ	43	12 ± 12	21
4-Hydroxybenzoic acid	0.38	6.7	410	38 ± 76	30
Ethyl gallate	0.0048	0.05	0.05	0.05	1
Gallic acid	0.056	0.098	3.2	0.98 ± 1.2	6
Protocatechuic acid	0.12	< LOQ	32	3.9 ± 9	17
Salicylic acid	0.96	1.4	360	41 ± 93	30
Syringic acid	0.14	< LOQ	9.8	2.8 ± 4	7
Vanillic acid	0.32	0.44	4.9	2 ± 1.5	12
***Hydroxycinnamic acids***					
Caffeic acid	1.1	2.7	2.8	2.8 ± 0.05	2
Chlorogenic acid	0.65	< LOQ	8.1	4.2 ± 3.3	4
Cinnamic acid	4.3	16	24	20 ± 5.7	2
Dihydrocaffeic acid	0.33	< LOQ	290	88 ± 120	6
Dihydroferulic acid	0.71	1.2	8.3	3.4 ± 2.3	9
Ferulic acid/Isoferulic acid	0.19	< LOQ	5	1.3 ± 1.2	29
p-Coumaric acid	0.092	< LOQ	23	5.6 ± 7.8	17
Sinapic acid	0.17	< LOQ	2	1.2 ± 1.2	6
***Hydroxyphenylacetic acids***					
3-(3-Hydroxyphenyl)propionic acid	0.76	< LOQ	77	8.5 ± 20	18
4-Hydroxyphenylacetic acid	12	< LOQ	12000	970 ± 2800	23
Homovanillic acid	3.4	< LOQ	14	6.9 ± 4.2	11
Homoprotocatechuic acid	1.3	4.4	65	27 ± 28	5
***Lignans***					
Enterodiol	0.034	0.14	110	22 ± 42	6
Enterolactone	0.038	0.21	1.9	0.54 ± 0.5	11
***Others***					
2,6-Dimethoxyphenol	0.12	0.33	0.39	0.36 ± 0.04	2
3,5-Dimethoxy-4-hydroxyphenylacetic acid	0.86	2.9	24	8 ± 7.6	7
4-Methylcatechol	0.48	0.96	170	41 ± 72	5
Catechol	10	< LOQ	2100	410 ± 550	24
Hydroxytyrosol	0.068	0.16	23	4.3 ± 8.6	7
Pyrogallol	2.8	3.6	5.4	4.4 ± 0.84	5
Urolithin A	0.028	0.15	0.39	0.27 ± 0.17	2
***Stilbenes***					
Dihydroresveratrol	0.11	< LOQ	< LOQ	-	2
Polydatin	0.12	1	1	1	1
Pterostilbene	0.068	1.4	1.4	1.4	1
***Catechins***					
(-)-Epicatechin	0.48	1.2	3.5	2.4 ± 1.7	2
***Flavanones***					
(+/-)-Naringenin	0.072	< LOQ	13	2.1 ± 2.9	27
8-Prenylnaringenin	0.032	1.3	1.3	1.3	1
Hesperetin	0.026	0.4	2.3	0.96 ± 0.7	6
Xanthohumol	0.034	< LOQ	< LOQ	-	1
***Flavones***					
Apigenin	0.0094	0.047	1.8	0.38 ± 0.59	8
Diosmetin	0.03	< LOQ	1.6	0.27 ± 0.41	20
***Flavonols***					
Isorhamnetin	0.018	< LOQ	0.34	0.1 ± 0.12	10
Kaempferol	0.34	< LOQ	0.64	0.6 ± 0.06	5
Kaempferol-3-O-glucuronide	0.026	0.21	0.82	0.51 ± 0.43	2
***Isoflavones***					
Daidzein	0.068	< LOQ	67	16 ± 25	15
Daidzein-7-β-D-glucuronide	0.13	0.13	0.59	0.42 ± 0.22	5
Genistein	0.0094	0.08	1.1	0.35 ± 0.4	10
Genistein-7-β-D-glucuronide	0.11	< LOQ	1.9	1.1 ± 0.89	5
Genistein-7-sulfate	0.36	< LOQ	< LOQ	-	11
***Proanthocyanidins***					
Procyanidin C1	1.2	< LOQ	< LOQ	-	6

^e^The concentrations were calculated using the matrix-matched calibration curve and corrected with the recovery determined during the method validation

As previously mentioned, comparing the polyphenol concentrations to other studies is not straightforward since only a few published reports focused on polyphenols in human breast milk. A previous study by Jamnik et al. [38] investigated xenobiotics in breast milk from one individual over the first 211 days after birth, including several polyphenols. In that study, 8-prenylnargingenin, daidzein, enterodiol, and enterolactone were quantified at mean concentrations of 0.11 ng*mL^−1^, 0.032 ng*mL^−1^, 0.013 ng*mL^−1^, and < LOQ, respectively, which was lower than the values of 1.3 ng*mL^−1^, 16 ng*mL^−1^, 22 ng*mL^−1^, and 0.54 ng*mL^−1^, respectively, reported for the same analytes in the present study. Song et al. [45] investigated the phytochemical content in breast milk samples, collected at three different time points, from 17 mothers donated by the Cincinnati Children’s Hospital Medical Center and reported epicatechin, (+/-)-naringenin, hesperetin, and kaempferol at higher average concentrations (42 ng*mL^−1^, 60 ng*mL^−1^, 120 ng*mL^−1^, and 7 ng*mL^−1^, respectively) compared to the values reported here (2.4 ng*mL^−1^, 2.1 ng*mL^−1^, 0.96 ng*mL^−1^, and 0.6 ng*mL^−1^, respectively). Furthermore, Song et al. [45] detected epigallocatechin, epigallocatechin gallate, and quercetin, which were not detected in the Nigerian samples. Lu et al. [46] detected twelve different polyphenols in 89 breast milk samples from Hong Kong women. Higher mean concentrations were reported for quercetin, (+/-)-naringenin, caffeic acid, and protocatechuic acid (41 ng*mL^−1^, 110 ng*mL^−1^, 30 ng*mL^−1^, and 112 ng*mL^−1^, respectively) compared to the values of 2.1 ng*mL^−1^, 2.8 ng*mL^−1^, and 3.9 ng*mL^−1^ for (+/-)-naringenin, caffeic acid, and protocatechuic acid, respectively, in the present study. However, chlorogenic acid, (-)-epicatechin, and daidzein had similar average concentrations of 2 ng*mL^−1^, 9 ng*mL^−1^, and 15 ng*mL^−1^, respectively, compared to the present study. An increased consumption of e.g. tea, which is rich in flavanols, can lead to an increased quercetin concentration, which could explain the amount of quercetin found in Lu et al. [46], whereas an increased intake of legumes and seeds can lead to an increased enterodiol and enterlactone concentrations, as their parent molecule, matairesinol, is prevalent in legumes and seeds [68, 69]. The disparities in the type and concentrations of polyphenols found in the various studies can be attributed to several factors such as different diets of the mothers, differences in analytical sensitivities, and sample size, as well as seasonal and growth-related differences in polyphenol contents [67].

Polyphenols readily undergo phase II biotransformation in the small intestine and liver; hence, a higher concentration of glucuronidated, compared to unconjugated, metabolites are typically detected in urine [60, 70, 71]. Phase II conjugated metabolites, including daidzein-7-β-D-glucuronide and genistein-7-β-D-glucuronide, were detected in several breast milk samples albeit at low concentrations (0.42 ng*mL^−1^ and 1.1 ng/mL^−1^, respectively). Overall, genistein and daidzein were present in more breast milk samples than their respective glucuronides. Interestingly, when a sample contained both, the parent compound and the glucuronidated compound, the glucuronide concentration was usually higher than the parent compound (Fig. 2e). It must be noted that as polyphenols have several hydroxyl groups, different positional isomers are possible and only one isomer was included in this method. Thus, different positional isomers of conjugated metabolites could be missed, especially as the LODs for most of the conjugated metabolites were similar or lower than their respective parent compound, e.g., caffeic acid-3-β-D-glucuronide had an LOD of 0.0085 ng*mL^−1^ and caffeic acid had 0.55 ng*mL^−1^. To get a more complete picture of polyphenol biotransformation, additional analyses, for example, by untargeted workflows, would be beneficial [72]. Previous studies have also shown that phase II metabolites of other xenobiotics, such as plasticizers, pesticides, and phytoestrogens, can be found in breast milk [33, 41, 57]. Further research is needed to study the pathways and presence of polyphenols and their biotransformation products in human milk.

**Fig. 3:**
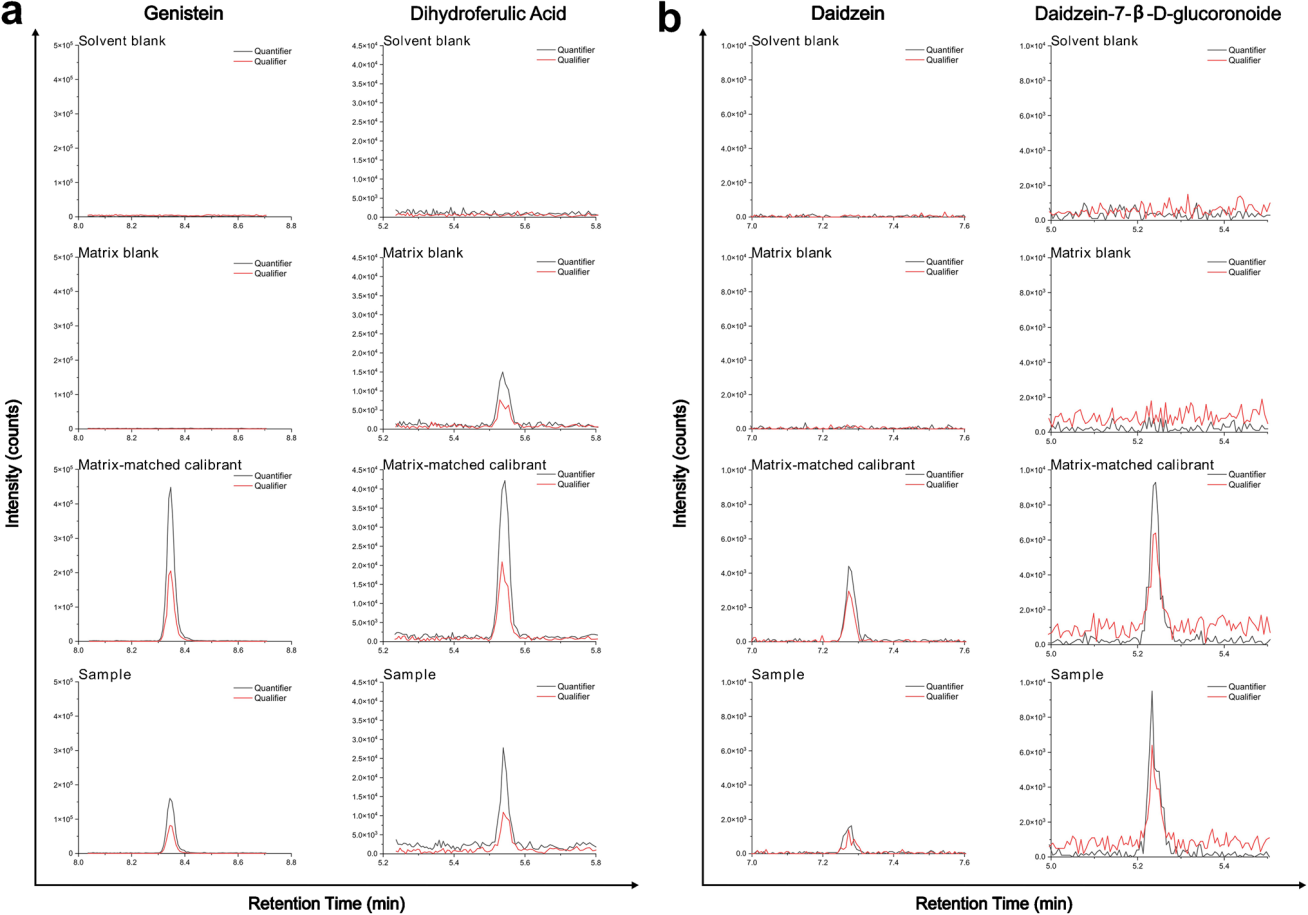
**a** MRM chromatograms (quantifier and qualifier ions) of a solvent blank, a non-spiked breast milk “blank,” a matrix-matched calibrant (0.43 ng*mL^−1^ for genistein and 1.5 ng*mL^−1^ for dihydroferulic acid), and a breast milk sample obtained from a Nigerian mother. **b** MRM chromatograms (quantifier and qualifier ions) of daidzein and daizein-7-β-D-glucuronide from the same mother and same timepoint, with the MRM chromatograms of a solvent blank, a matrix-matched breast milk “blank,” and a matrix-matched calibrant (0.037 ng*mL^−1^ for daidzein and 0.11 ng*mL^−1^ for daizein-7-β-D-glucuronide)

A rough estimation of the exposure levels of infants to polyphenols was conducted. In order to exclude other possible polyphenol sources, e.g., from complementary foods, only breast milk sampled at month one after birth was used for this estimation. Analytes that were detected below the LOQ value were considered positive and the corresponding LOQ value was applied (i.e., upper bound scenario). An average infant body weight of 4 kg [73] and a daily consumption of 500 mL breast milk were assumed. Based on this estimation (individual, median, and mean daily intakes are reported in Table S6), it was derived that the approximate daily intake per polyphenol detected was in the lower microgram per kilogram body weight range, with the median analyte concentration ranging from 0.0044 µg*kg^−1^ body weight per day (phloretin) to 31 µg*kg^−1^ body weight per day (catechol). The most common analytes detected in the breast milk samples were 4-hydroxybenzoic acid, diosmetin, salicylic acid, ferulic acid, and (+/-)-naringenin, and had an estimated median daily intake of 1.9 µg*kg^−1^, 0.022 µg* kg^−1^, 0.54 µg* kg^−1^, 0.094 µg* kg^−1^, and 0.14 µg* kg^−1^ of body weight, respectively. Though numerous known polyphenols have not been included in this method, the sum of the investigated polyphenols detected yielded an estimated median daily intake of 57 µg*kg^−1^. It must be noted that these estimations were calculated for only one sampling time point and should be interpreted with caution. However, the estimates provide rough insights into the exposure of infants towards a large panel of polyphenols. Therefore, to better ascertain the daily polyphenol exposure, further studies are needed that include a larger sample size and information on the polyphenol content of the food consumed by the mothers on the day of sampling (Fig. 3).

## Conclusion and outlook

In conclusion, the successful optimization and in-house validation of an LC–MS/MS method targeting 86 polyphenols that are representatives of all major polyphenol classes in human breast milk are presented. Despite low sample volumes, a high-throughput sample preparation, and a wide variety of analytes, this approach demonstrated high sensitivity while retaining high recoveries and low signal suppression and enhancement effects. Moreover, the application of the method in a pilot study demonstrated its feasibility to be readily used in large cohort studies. Thus, it can be applied to investigate and better comprehend the transfer of ingested dietary polyphenols to breast milk, which would be beneficial in further nutritional intervention and prevention studies. Moreover, it can also be used to investigate human metabolism in vivo. Its application in large cohorts would also aid the advanced investigation of the impact of polyphenols in nutritional intervention studies. Finally, this method can also be applied, to better understand the transfer of polyphenols to newborns. Especially in the era of exposome-type research, it may reveal new insights on potential health benefits and polyphenol impact on microbiome development and of co-exposure and mixture of toxicological effects with other xenobiotics that infants are exposed to via their diet and environment.

### Supplementary information

Below is the link to the electronic supplementary material.

Supplementary file1 (DOCX 3.32 MB)
