# Supplementary material for: Polyphenol exposure of mothers and infants assessed by LC–MS/MS based biomonitoring in breast milk
Source: Anal Bioanal Chem. 2024 Feb 16;416(7):1759–74. doi: 10.1007/s00216-024-05179-y (PMC10899372; doi:10.1007/s00216-024-05179-y)
Supplement: Supplementary file 1 — Supplementary file1 (DOCX 3.32 MB) [file 216_2024_5179_MOESM1_ESM.docx]

**Supplementary Information**

**Polyphenol exposure of mothers and infants assessed by
LC-MS/MS based biomonitoring in breast milk**

Sabrina Berger^†^, Ian Oesterle^†,‡,§^, Kolawole I. Ayeni^†,⁑^, Chibundu N. Ezekiel^+^, Annette Rompel^‡^, and Benedikt Warth^†,||,*^

^†^University of Vienna, Faculty of Chemistry, Department of Food Chemistry and Toxicology, 1090 Vienna, Austria

^‡^Universität Wien, Fakultät für Chemie, Institut für Biophysikalische Chemie, 1090 Wien, Austria; www.bpc.univie.ac.at

^§^University of Vienna, Vienna Doctoral School of Chemistry (DoSChem), 1090 Vienna, Austria

^⁑^Department of Microbiology, Babcock University, Ilishan Remo, Ogun State, Nigeria

^+^University of Natural Resource and Life Science Vienna (BOKU), Department of Agrobiotechnology (IFA-Tulln), Institute for Bioanalytics and Agro-Metabolomics, Konrad-Lorenz Str. 20, 3430 Tulln, Austria.

^||^Exposome Austria, Research Infrastructure and National EIRENE Node, Austria

*Corresponding author: Benedikt Warth. benedikt.warth@univie.ac.at, +43-1-4277-70806

**Figure S1:** Molecular structures of the analytes included in this study……………………………...…….2

**Table S1:** Supplier information on the reagents, solvents, and chemicals used……...……………...…...….3

**Table S2:** Multiple reaction monitoring (MRM) parameters………….…………...…..…………..…….…8

**Table S3:** LC gradient……………………………...……………………………………………...……....12

**Table S4:** Spiking levels, recovery, intermediate precision and repeatability………......................….…...13

**Table S5:** Method validation outcome……………………………..……………..………..…………..….17

**Figure S2:** Analytical figures of merit obtained during the method validation of the remaining eight polyphenol classes…………………………………………………………………………………………20

**Table S6:** Estimated daily polyphenol intake……………………..……………..………..…………..…...21

**Table S7:** Calibration curves………………………………………………………………………………23

**References**……………………………………………………………………………………….………..33


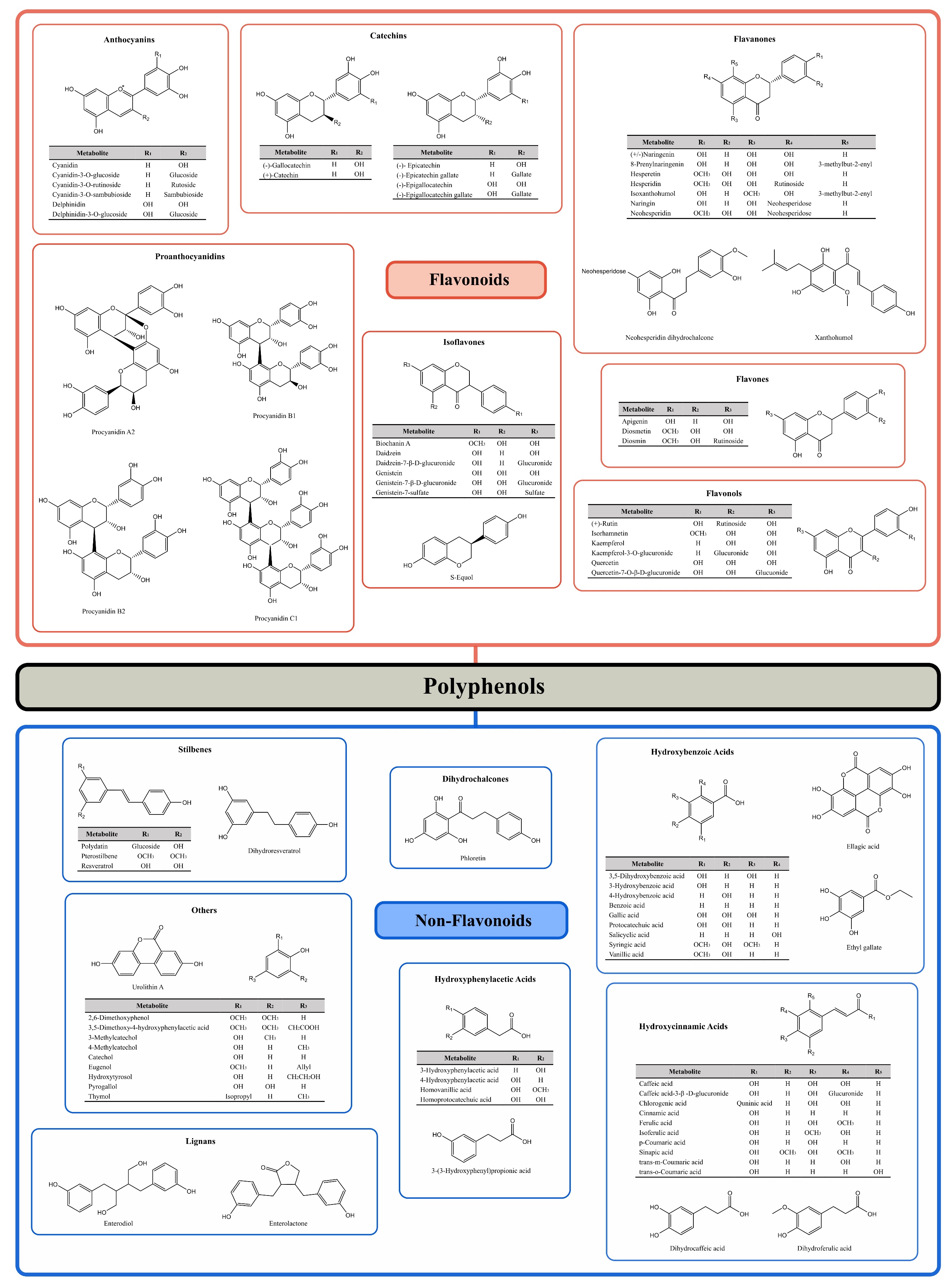


**Figure S1.** Molecular structures of the analytes included in this study, divided into their polyphenol classes [1].

**Table S1.** Supplier information on the reagents, solvents, and chemicals used. The polyphenol standards are the same as reported by Oesterle et al. [1]

| **Item** | **Supplier** | **Item Number** | **Lot Number** | **Purity** |
| --- | --- | --- | --- | --- |
| ***Dihydrochalcones*** |  |  |  |  |
| Phloretin | Cayman Chemical Company | 14452-1g | 0447930-17 | 1 |
| ***Hydroxybenzoic Acids*** |  |  |  |  |
| Caffeic acid | Sigma | C0625 | 089K1114 | 0.993 |
| 3-Hydroxybenzoic acid | Sigma-Aldrich | 36333-100mg | BCBV2381 | 0.998 |
| 4-Hydroxybenzoic acid | Sigma | 240141-50g | BCCB8991 | 1 |
| Benzoic acid | Sigma-Aldrich | 242381 | MKBG9391V | 0.999 |
| Ellagic acid | Sigma | E-2250 | 70K1240 | 0.99 |
| Ethyl gallate | Phytolab | 83080-100mg | 5950 | 1 |
| Gallic acid | Sigma | G7384-100g | SLBW1280 | 1 |
| Protocatechuic acid | Sigma | P-5630 | 072K3446 | 0.998 |
| Salicyclic acid | Sigma-Aldrich | 247588 | 09712LE | 0.998 |
| Syringic acid | Sigma | S6881-5g | BCCB1235 | 0.995 |
| Vanillic acid | Sigma | V-2250 | 50H7714 | 0.99 |
| ***Hydroxycinnamic Acids*** |  |  |  |  |
| Caffeic acid | Sigma | C0625 | 089K1114 | 99.3 |
| Caffeic acid-3-β-D-glucuronide | Toronto Research Chemicals | C080015-1mg | 1-KMR-141-3 | 0.9965 |
| Chlorogenic acid | Aldrich | C3878 | SLBL9959V | 0.99 |
| Cinnamic acid | Fluka | 96340 | 408492/1 | 0.993 |
| Dihydrocaffeic acid | Aldrich | 10.260-1 | 1235963 | 0.98 |
| Dihydroferulic acid | Aldrich | 17803 | BCBH4069V | 1 |
| Ferulic acid | Fluka | 46278 | 357835/1 | 1.0004 |
| Isoferulic acid | Toronto Research Chemicals | H946180-2.5mg | 12-XJZ-152-1 | 0.98 |
| p-Coumaric acid | Sigma | C9008-5g | 095K1340 | 0.997 |
| Sinapic acid | Sigma | D7927 | 0000071240 | 0.985 |
| trans-m-Coumaric acid | Phytolab | 83258-10mg | 10022 | 1 |
| **Item** | **Supplier** | **Item Number** | **Lot Number** | **Purity** |
| trans-o-Coumaric acid | Phytolab | 82343-100mg | 4341 | 0.9985 |
| ***Hydroxyphenylacetic Acids*** |  |  |  |  |
| 3-(3-Hydroxyphenyl)propionic acid | Sigma-Aldrich | 91779-10mg | BCCB6466 | 0.998 |
| 3-Hydroxyphenylacetic acid | Aldrich | H49901-5g | STBB5523 | 0.992 |
| 4-Hydroxyphenylacetic acid | Sigma | H50004-5g | BCCB4700 | 0.997 |
| Homoprotocatechuic acid | Aldrich | 85,021-7 | S23418-404 | 0.994 |
| Homovanillic acid | Sigma-Aldrich | 69673-25mg | BCCC5315 | 0.996 |
| ***Lignans*** |  |  |  |  |
| Enterodiol | Phytolab | 80436-10mg | 11769 | 0.9846 |
| Enterolactone | Phytolab | 80437-10mg | 5185 | 0.9907 |
| ***Others*** |  |  |  |  |
| 2,6-Dimethoxyphenol | Aldrich | D135550 | MKBG7714V | 0.994 |
| 3,5-Dimethoxy-4-hydroxyphenylacetic acid | Aldrich | 631310 | 07406JC | 0.991 |
| 3-Methylcatechol | Aldrich | M34006 | MKBB9900 | 0.991 |
| 4-Methylcatechol | Aldrich | M34200 | MKBB9773 | 0.988 |
| Catechol | Sigma | C-9510 | 96F-0536 | 0.99 |
| Eugenol | Extrasynthese | 6178 S | 08-0921/0 | 0.9978 |
| Hydroxytyrosol | Extrasynthese | 4999 S | 12-0520/0 | 1 |
| Pyrogallol | Merck | 612 | 1151574 | 0.995 |
| Thymol | Sigma | 16254 | SZBB0460V | 0.994 |
| Urolithin A | Sigma-Aldrich | SML1791-5mg | 0000076439 | 0.988 |
| ***Stilbenes*** |  |  |  |  |
| Dihydroresveratrol | Toronto Research Chemicals | D678960-1mg | 2-MJJ-97-1 | 0.98 |
| Polydatin | Extrasynthese | 4974 S | 02-0822/0 | 0.992 |
| Pterostilbene | Toronto Research Chemicals | P839890-50mg | 1-NYL-63-1 | 0.98 |
| Resveratrol | Extrasynthese | 4963 S | 02-1219/0 | 0.997 |
| **Item** | **Supplier** | **Item Number** | **Lot Number** | **Purity** |
| ***Anthocyanins*** |  |  |  |  |
| Cyanidin chloride | Extrasynthese | 0909 S | 38-0723/0 | 0.98 |
| Cyanidin-3-O-glucoside chloride | Extrasynthese | 0915 S | 45 – 1114/0 | 0.976 |
| Cyanidin-3-O-rutinoside chloride | Extrasynthese | 0914 S | 31 – 0917/0 | 0.973 |
| Cyanidin-3-O-sambubioside chloride | Extrasynthese | 0949 S | 04 – 0311/0 | 0.972 |
| Delphinidin chloride | Extrasynthese | 0904 S | 63 – 0814/0 | 0.978 |
| Delphinidin-3-O-glucoside chloride | Extrasynthese | 0938 S | 27 – 1023/0 | 0.995 |
| ***Catechins*** |  |  |  |  |
| (-)-Epicatechin | Sigma | E-1753 | 32H2519 | 0.98 |
| (-)-Epicatechin gallate | Extrasynthese | 0978 S | 12-0721/0 | 0.993 |
| (-)-Epigallocatechin | Extrasynthese | 0979 S | 11-0703/0 | 0.992 |
| (-)-Epigallocatechin gallate | Sigma | E4143 | 031M1175V | 0.95 |
| (-)-Gallocatechin | Extrasynthese | 0973 S | 05-1014/0 | 0.989 |
| (+)-Catechin | Extrasynthese | 0976 S | 25-0204/0 | 1 |
| ***Flavanones*** |  |  |  |  |
| (+/-)-Naringenin | Sigma | N5893-1g | BCBC1784 | 0.96 |
| 8-Prenylnaringenin | Sigma | 75119-5mg | BCCB4227 | 1 |
| Hesperetin | Cayman Chemical Company | Cat. 10006084 | 121938-5 | 0.984 |
| Hesperidin | Fluka | 52040 | 1117099 | 0.923 |
| Isoxanthohumol | Extrasynthese | 1367 S | 01-1020/0 | 0.995 |
| Naringin hydrate | Alfa Aesar | L10163 | 10126866 | 0.98 |
| Neohesperidin | Extrasynthese | 1132 S | 09-0924/0 | 0.996 |
| Neohesperidin dihydrochalcone | SAFC | W381101-25g-K | MKBD9742V | 0.85 |
| Xanthohumol | Extrasynthese | 1346 S | 03-0524/0 | 1 |
| ***Flavones*** |  |  |  |  |
| Apigenin | Sigma | 10798-25mg | E445301/1V | 0.954 |
| Diosmetin | Extrasynthese | 1108 S | 15-0312/0 | 0.998 |
| **Item** | **Supplier** | **Item Number** | **Lot Number** | **Purity** |
| Diosmin | Extrasynthese | 1109 S | 11-1021/0 | 0.996 |
| ***Flavonols*** |  |  |  |  |
| (+)-Rutin trihydrate | Aldrich | R230-3 | 10422EW | 0.95 |
| Isorhamnetin | Extrasynthese | 1120 S | 32-0215/0 | 0.993 |
| Kaempferol | Sigma | 60010 | BCCC4134 | 0.997 |
| Kaempferol-3-O-glucuronide | Extrasynthese | 1356 S | 03-0302/0 | 0.996 |
| Quercetin | Sigma | Q4951-10g | 060M1196V | 0.98 |
| Quercetin-7-O-β-D-glucuronide | Toronto Research Chemicals | Q509515-2.5mg | 12-QFY-8-3 | 0.9539 |
| ***Isoflavones*** |  |  |  |  |
| Biochanin A | Extrasynthese | 1349 S | 01-0106/0 | 0.999 |
| Daidzein | Extrasynthese | 1370 S | 03-0204/0 | 0.995 |
| Daidzein-7-β-D-glucuronide potassium salt | Toronto Research Chemicals | D103510-5mg | 3-LGA-24-2 | 0.95 |
| Genistein | Extrasynthese | 1372 S | 01 – 0114/0 | 1 |
| Genistein-7-β-D-glucuronide | Toronto Research Chemicals | G350015-2.5mg | 3-JAE-172-1 | 0.95 |
| Genistein-7-sulfate sodium salt | Toronto Research Chemicals | G350045-2.5mg | 10-JLI-110-4 | 0.9989 |
| S-equol | Sigma | SML2147-5mg | 0000081122 | 0.97 |
| ***Proanthocyanidins*** |  |  |  |  |
| Procyanidin A2 | Extrasynthese | 0985 S | 05-0128/0 | 0.999 |
| Procyanidin B1 | Phytolab | 89764-5mg | 14872 | 0.9739 |
| Procyanidin B2 | Toronto Research Chemicals | P755830-1mg | 52-GHZ-187-1 | 0.9826 |
| Procyanidin C1 | Phytolab | 89537-5mg | 15436 | 0.974 |
| ***Reagents*** |  |  |  |  |
| Methanol | Honeywell | 34966 | Various | LC-MS grade |
| Acetonitrile | Honeywell | 34967 | Various | LC-MS grade |
| Water | VWR | 63645.320 | Various | LC-MS grade |
| Formic acid | Bartelt | SO9679B001 | 1373 811 | UPLC-MS Optigrade |
| Hexane | Roth | 7567.1 | Various | ≥ 96% |
| **Item** | **Supplier** | **Item Number** | **Lot Number** | **Purity** |
| Ethylacetate | Sigma-Aldrich | 33211-1L-R | SZBD128SV | ≥ 99.5% |
| 2-Propanol | Honeywell | 34965 | Various | LC-MS grade |
| Magnesium sulfate, anhydrous | Acros Organics | 4134850000 | A0379632 | 97% |
| Sodium chloride | Roth | 0962.2 | 390289120 | ≥ 99.8% |

**Table S2.** Multiple reaction monitoring (MRM) parameters of the included analytes. All values that could not be determined are marked as n.d. (not determined). The delustering potential for all analytes was set to -10V.

| **Analyte** | **Retention Time**  **(min)** | **Total Retention Time Window (s)** | **Parent Ion**  ***m/z*** | **Product Ions (Quantifier/Qualifier)**  ***m/z*** | **Collision Energy (V)** | **Cell Exit Potential (V)** | **Ion**  **Ratio**  **(%)** |
| --- | --- | --- | --- | --- | --- | --- | --- |
| ***Dihydrochalcones*** |  |  |  |  |  |  |  |
| Phloretin | 8.3 | 20 | 272.982 | 166.9/123.2 | -26/-30 | -17/-47 | 22 |
| ***Hydroxybenzoic Acids*** |  |  |  |  |  |  |  |
| 3,5-Dihydroxybenzoic acid | 2.6 | 25 | 152.862 | 108.8/67 | -28/-28 | -17/-19 | 12 |
| 3-Hydroxybenzoic acid | 4.7 | 20 | 136.873 | 92.9/64.9 | -16/-32 | -11/-35 | 9 |
| 4-Hydroxybenzoic acid | 4.0 | 20 | 136.891 | 92.9/65.1 | -18/-38 | -23/-9 | 16 |
| Benzoic acid | 6.3 | 20 | 120.945 | 77.1 | -18 | -13 | n.d.^a^ |
| Ellagic acid | 5.9 | 25 | 300.873 | 145.0/255.3 | -50/-16 | -9/-19 | n.d.^[[1]](#footnote-1)^ |
| Ethyl gallate | 5.4 | 20 | 196.970 | 123.9/168.9 | -26/-24 | -23/-11 | 58 |
| Gallic acid | 1.3 | 20 | 168.915 | 125.0/79.0 | -20/-26 | -9/-7 | 16 |
| Protocatechuic acid | 2.7 | 25 | 152.939 | 108.1/80.9 | -34/-26 | -11/-11 | 26 |
| Salicylic acid | 6.6 | 20 | 136.937 | 92.9/65.1 | -24/-36 | -13/-13 | 15 |
| Syringic acid | 4.8 | 20 | 196.943 | 181.9/121.1 | -18/-22 | -17/-23 | 15 |
| Vanillic acid | 4.6 | 20 | 166.926 | 152.0/108.1 | -18/-26 | -13/-7 | 64 |
| ***Hydroxycinnamic Acids*** |  |  |  |  |  |  |  |
| Caffeic acid | 4.7 | 20 | 178.914 | 107.0/89.0 | -30/-42 | -7/-7 | 85 |
| Caffeic acid-3-β-D-glucuronide | 4.2 | 20 | 355.074 | 135.0/179.2 | -36/-30 | -21/-5 | 110 |
| Chlorogenic acid | 4.3 | 20 | 353.006 | 191.0/85.0 | -20/-52 | -13/-11 | 17 |
| Cinnamic acid | 7.7 | 20 | 146.936 | 77.0/103.0 | -28/-14 | -7/-17 | 660 |
| Dihydrocaffeic acid | 4.4 | 20 | 180.971 | 58.9/121.0 | -22/-22 | -9/-9 | 42 |
| Dihydroferulic acid | 5.6 | 20 | 194.954 | 136.0/121.1 | -24/-34 | -11/-9 | 48 |
| Ferulic acid/Isoferulic acid | 5.8 | 20 | 192.941 | 134.1/178.0 | -22/-18 | -9/-11 | 56 |
| p-Coumaric acid | 5.5 | 20 | 162.923 | 119.0/92.9 | -22/-38 | -13/-11 | 17 |
| **Analyte** | **Retention Time**  **(min)** | **Total Retention Time Window (s)** | **Parent Ion**  ***m/z*** | **Product Ions (Quantifier/Qualifier)**  ***m/z*** | **Collision Energy (V)** | **Cell Exit Potential (V)** | **Ion**  **Ratio**  **(%)** |
| Sinapic acid | 5.9 | 20 | 222.999 | 208.0/164.1 | -18/-18 | -13/-11 | 55 |
| trans-m-Coumaric acid | 6.0 | 20 | 162.864 | 119.1/91.0 | -12/-32 | -7/-33 | 18 |
| trans-o-Coumaric acid | 6.5 | 20 | 162.942 | 118.8/116.9 | -18/-34 | -47/-17 | 18 |
| ***Hydroxyphenylacetic Acids*** |  |  |  |  |  |  |  |
| 3-(3-Hydroxyphenyl)propionic acid | 5.7 | 20 | 164.973 | 106.1/121.0 | -28/-16 | -1/-23 | 310 |
| 3-Hydroxyphenylacetic acid | 4.8 | 20 | 150.972 | 107.1/79.2 | -12/-28 | -11/-39 | 10 |
| 4-Hydroxyphenylacetic acid | 4.4 | 20 | 150.937 | 79.1/107.1 | -22/-10 | -9/-7 | 350 |
| Homovanillic acid | 4.8 | 20 | 180.887 | 122.0/135.9 | -24/-10 | -19/-21 | 9 |
| Homoprotocatechuic acid | 3.4 | 20 | 166.928 | 123.0/121.9 | -12/-30 | -13/-7 | 14 |
| ***Lignans*** |  |  |  |  |  |  |  |
| Enterodiol | 7.4 | 20 | 300.927 | 253.1/271.2 | -30/-30 | -11/-23 | 33 |
| Enterolactone | 8.6 | 20 | 296.944 | 253.1/107.1 | -28/-32 | -13/-17 | 53 |
| ***Others*** |  |  |  |  |  |  |  |
| 2,6-Dimethoxyphenol | 6.2 | 20 | 154.833 | 123.1/77.1 | 15/25 | 10/4 | 48 |
| 3,5-Dimethoxy-4-hydroxyphenylacetic acid | 5.0 | 20 | 211.02 | 167.1/136.9 | -10/-32 | -21/-9 | 55 |
| 3-Methylcatechol | 5.8 | 20 | 122.97 | 107.8/95.0 | -24/-22 | -55/-7 | 24 |
| 4-Methylcatechol | 5.4 | 20 | 122.935 | 108.0/104.9 | -30/-28 | -11/-11 | 51 |
| Catechol | 2.6 | 25 | 108.908 | 91.0/80.9 | -26/-22 | -13/-39 | 66 |
| Eugenol | 9.2 | 20 | 164.896 | 136.8/123.9 | 13/23 | 14/6 | 40 |
| Hydroxytyrosol | 2.7 | 25 | 152.936 | 123.1/122.5 | -20/-30 | -13/-9 | 10 |
| Pyrogallol | 1.5 | 25 | 124.932 | 69.0/79.0 | -24/-26 | -11/-19 | 180 |
| Thymol | 5.8 | 20 | 149.012 | 134.0/133.6 | -20/-16 | -11/-23 | 39 |
| Urolithin A | 7.3 | 20 | 226.838 | 197.8/181.8 | -44/-38 | -11/-13 | 44 |
| ***Stilbenes*** |  |  |  |  |  |  |  |
| Dihydroresveratrol | 7.2 | 20 | 230.956 | 121.1/137.1 | 21/25 | 6/4 | 130 |
| Polydatin | 5.8 | 20 | 389.061 | 227.3/143.1 | -16/-58 | -13/-21 | 21 |
| Pterostilbene | 11.0 | 20 | 257.118 | 242.4/133.2 | 33/27 | 14/12 | 260 |
| **Analyte** | **Retention Time**  **(min)** | **Total Retention Time Window (s)** | **Parent Ion**  ***m/z*** | **Product Ions (Quantifier/Qualifier)**  ***m/z*** | **Collision Energy (V)** | **Cell Exit Potential (V)** | **Ion**  **Ratio**  **(%)** |
| Resveratrol | 7.1 | 20 | 226.977 | 185.0/142.9 | -26/-34 | -29/-51 | 57 |
| ***Anthocyanins*** |  |  |  |  |  |  |  |
| Cyanidin | 4.5 | 30 | 286.837 | 230.9/213.1 | 33/39 | 20/24 | 33 |
| Cyanidin-3-O-glucoside | 4.5 | 30 | 448.866 | 286.8/212.8 | 35/77 | 44/10 | 13 |
| Cyanidin-3-O-rutinoside | 4.6 | 30 | 595.127 | 287.0/449.1 | 31/29 | 24/22 | 16 |
| Cyanidin-3-O-sambubioside | 4.5 | 30 | 581.015 | 286.9/212.9 | 35/95 | 40/12 | 13 |
| Delphinidin | 5.1 | 20 | 302.813 | 229.0/201.2 | 37/37 | 14/18 | 29 |
| Delphinidin-3-O-glucoside | 4.2 | 30 | 465.163 | 303.1/228.9 | 35/71 | 24/18 | 16 |
| ***Catechins*** |  |  |  |  |  |  |  |
| (-)- Epicatechin | 5.0 | 20 | 288.948 | 245.1/203.0 | -20/-26 | -17/-15 | 46 |
| (-)-Epicatechin gallate | 5.9 | 20 | 440.878 | 169.0/289.0 | -22/-28 | -27/-17 | 70 |
| (-)-Epigallocatechin | 4.3 | 25 | 304.943 | 124.9/179.0 | -26/-20 | -35/-9 | 57 |
| (-)-Epigallocatechin gallate | 5.1 | 20 | 457.091 | 168.9/125.0 | -24/-48 | -11/-13 | 41 |
| (-)-Gallocatechin | 2.6 | 25 | 304.962 | 124.9/219.0 | -22/-20 | -13/-37 | 31 |
| (+)-Catechin | 4.4 | 20 | 288.903 | 123.2/203.0 | -42/-26 | -5/-33 | 110 |
| ***Flavanones*** |  |  |  |  |  |  |  |
| (+/-)-Naringenin | 8.3 | 20 | 271.004 | 151.0/119.1 | -24/-34 | -13/-3 | 56 |
| 8-Prenylnaringenin | 10.6 | 20 | 339.074 | 219.0/119.0 | -26/-40 | -29/-7 | 58 |
| Hesperetin | 6.5 | 20 | 609.045 | 301.0/286.2 | -34/-52 | -25/-21 | 47 |
| Hesperidin | 8.6 | 20 | 300.976 | 163.9/135.9 | -32/-38 | -9/-7 | 29 |
| Isoxanthohumol | 9.4 | 20 | 352.998 | 118.9/233.1 | -26/-26 | -27/-55 | 89 |
| Naringin | 6.3 | 20 | 580.954 | 272.8/152.8 | 21/65 | 16/22 | 55 |
| Neohesperidin | 6.5 | 20 | 610.963 | 449.1/303.0 | 11/27 | 32/18 | 290 |
| Neohesperidin dihydrochalcone | 7.1 | 20 | 611.058 | 303.0/125.0 | -46/-52 | -19/-13 | 25 |
| Xanthohumol | 12.3 | 20 | 352.996 | 233.2/119.1 | -24/-56 | -29/-15 | 83 |
| ***Flavones*** |  |  |  |  |  |  |  |
| Apigenin | 8.3 | 30 | 268.945 | 117.0/151.0 | -44/-36 | -9/-19 | 55 |
| **Analyte** | **Retention Time**  **(min)** | **Total Retention Time Window (s)** | **Parent Ion**  ***m/z*** | **Product Ions (Quantifier/Qualifier)**  ***m/z*** | **Collision Energy (V)** | **Cell Exit Potential (V)** | **Ion**  **Ratio**  **(%)** |
| Diosmetin | 6.3 | 20 | 607.023 | 298.9/283.8 | -36/-68 | -43/-17 | 79 |
| Diosmin | 8.6 | 25 | 298.925 | 284.1/256.0 | -32/-40 | -19/-27 | 14 |
| ***Flavonols*** |  |  |  |  |  |  |  |
| (+)-Rutin | 5.7 | 20 | 609.041 | 300.1/301.0 | -44/-38 | -9/-21 | 51 |
| Isorhamnetin | 8.6 | 25 | 314.919 | 299.8/151.0 | -34/-34 | -17/-5 | 22 |
| Kaempferol | 8.5 | 35 | 284.952 | 117.1/184.9 | -54/-40 | -13/-15 | 120 |
| Kaempferol-3-O-glucuronide | 6.3 | 20 | 460.963 | 284.9/112.9 | -32/-20 | -17/-17 | 35 |
| Quercetin | 7.6 | 25 | 300.911 | 151.0/178.9 | -32/-26 | -9/-27 | 47 |
| Quercetin-7-O-β-D-glucuronide | 5.8 | 20 | 477.062 | 300.8/150.9 | -28/-48 | -25/-11 | 17 |
| ***Isoflavones*** |  |  |  |  |  |  |  |
| Biochanin A | 10.5 | 20 | 282.89 | 267.9/239.0 | -26/-44 | -21/-21 | 30 |
| Daidzein | 7.3 | 20 | 253.013 | 131.9/224.0 | -50/-36 | -19/-21 | 150 |
| Daidzein-7-β-D-glucuronide | 5.3 | 20 | 429.141 | 252.8/113.0 | -32/-22 | -23/-17 | 74 |
| Genistein | 8.4 | 20 | 268.962 | 133.0/132.1 | -38/-54 | -15/-13 | 46 |
| Genistein-7-β-D-glucuronide | 6.1 | 20 | 444.932 | 269.0/174.9 | -40/-18 | -45/-17 | 53 |
| Genistein-7-sulfate | 7.1 | 30 | 348.798 | 268.9/133.1 | -38/-58 | -53/-7 | 18 |
| S-Equol | 8.4 | 20 | 240.938 | 120.9/119.0 | -20/-26 | -19/-13 | 72 |
| ***Proanthocyanidins*** |  |  |  |  |  |  |  |
| Procyanidin A2 | 6.0 | 20 | 574.998 | 284.9/448.9 | -38/-26 | -35/-35 | 71 |
| Procyanidin B1 | 4.1 | 20 | 579.296 | 409.2/288.9 | 27/19 | 26/18 | 83 |
| Procyanidin B2 | 4.7 | 20 | 578.552 | 426.8/409.0 | 23/29 | 32/34 | 83 |
| Procyanidin C1 | 5.1 | 20 | 867.202 | 579.1/577.2 | 21/21 | 26/36 | 66 |

**Table S3.** LC gradient applied in the final method.

| **Time [min]** | **Eluent A [%]** | **Eluent B [%]** |
| --- | --- | --- |
| 0 | 95 | 5 |
| 2 | 95 | 5 |
| 12 | 36 | 64 |
| 12.01 | 5 | 95 |
| 14 | 5 | 95 |
| 14.01 | 95 | 5 |
| 16 | 95 | 5 |

**Table S4.** Concentration of the three different spiking levels: low level (LL), middle level (ML), and high level (HL), used during validation for each analyte. The calculated recovery (R_E_), Intermediate precision (RSD_R_) and interday repeatability (RSD_r_) are given. Figures of merit which could not be determined are listed as n.d.

| **Analyte** | **Spiking Level (LL/ML/HL)**  **(ng*mL^-1^)** | **R_E_ ± RSD_R_ (LL) (%)** | **R_E_ ± RSD_R_ (ML) (%)** | **R_E_ ± RSD_R_ (HL) (%)** | **RSD_r_**  **(LL/ML/HL)**  **(%)** |
| --- | --- | --- | --- | --- | --- |
| ***Dihydrochalcones*** |  |  |  |  |  |
| Phloretin^[[2]](#footnote-2)^ | 0.22/2.8/8.8 | 98 ± 10 | 96 ± 12 | 94 ± 12 | 7/10/7 |
| ***Hydroxybenzoic Acids*** |  |  |  |  |  |
| 3,5-Dihydroxybenzoic acid | 2.9/39/120 | 39 ± 36 | 47 ± 8 | 46 ± 13 | 85/36/43 |
| 3-Hydroxybenzoic acid | 18/240/710 | 92 ± 7 | 99 ± 6 | 96 ± 9 | 4/5/4 |
| 4-Hydroxybenzoic acidb | 1.1/14/42 | 75 ± 31 | 93 ± 6 | 95 ± 8 | 30/5/3 |
| Benzoic acid^b^ | 47/630/1900 | n.d. | 80 ± 12 | 88 ± 14 | n.d./10/9 |
| Ellagic acid^b^ | 23/310/930 | n.d. | 10 ± 39 | 6 ± 26 | n.d./19/6 |
| Ethyl gallate | 0.071/0.95/2.9 | 84 ± 5 | 90 ± 4 | 91 ± 9 | 8/7/3 |
| Gallic acid | 0.3/4.1/12 | 18 ± 53 | 40 ± 8 | 41 ± 14 | 50/28/37 |
| Protocatechuic acid | 0.2/2.6/7.9 | 42 ± 53 | 37 ± 12 | 46 ± 14 | 59/42/71 |
| Salicylic acid^b^ | 2.7/36/110 | 74 ± 31 | 92 ± 14 | 91 ± 19 | 32/6/4 |
| Syringic acid | 0.32/4.3/13 | 104 ± 26 | 91 ± 10 | 91 ± 12 | 27/7/5 |
| Vanillic acid | 2.1/28/84 | 101 ± 10 | 91 ± 7 | 89 ± 14 | 15/7/7 |
| ***Hydroxycinnamic Acids*** |  |  |  |  |  |
| Caffeic acid | 4/53/160 | 86 ± 21 | 91 ± 7 | 88 ± 8 | 26/5/3 |
| Caffeic acid-3-β-D-glucuronide | 0.2/2.7/8.2 | 63 ± 8 | 71 ± 9 | 72 ± 12 | 17/10/6 |
| Chlorogenic acid | 3.8/51/150 | 58 ± 19 | 59 ± 9 | 63 ± 10 | 5/8/3 |
| Cinnamic acid | 19/260/780 | 92 ± 15 | 89 ± 7 | 89 ± 8 | 17/7/5 |
| Dihydrocaffeic acid | 1.1/15/44 | 88 ± 22 | 89 ± 7 | 86 ± 8 | 17/7/4 |
| Dihydroferulic acid^b^ | 1.5/20/59 | 113 ± 27 | 95 ± 16 | 92 ± 15 | 24/10/8 |
| Ferulic acid/Isoferulic acidb | 1/14/41 | 80 ± 15 | 95 ± 6 | 93 ± 10 | 13/7/4 |
| p-Coumaric acid | 0.58/7.8/23 | 90 ± 11 | 96 ± 4 | 95 ± 7 | 14/3/3 |
| **Analyte** | **Spiking Level (LL/ML/HL)**  **(ng*mL^-1^)** | **R_E_ ± RSD_R_ (LL) (%)** | **R_E_ ± RSD_R_ (ML) (%)** | **R_E_ ± RSD_R_ (HL) (%)** | **RSD_r_**  **(LL/ML/HL)**  **(%)** |
| Sinapic acidb | 0.33/4.4/13 | 99 ± 34 | 95 ± 7 | 95 ± 9 | 30/10/3 |
| trans-m-Coumaric acid | 3.3/45/130 | 63 ± 42 | 99 ± 9 | 95 ± 10 | 27/4/4 |
| trans-o-Coumaric acid | 1.9/26/77 | 93 ± 14 | 94 ± 8 | 95 ± 8 | 7/6/4 |
| ***Hydroxyphenylacetic Acids*** |  |  |  |  |  |
| 3-(3-Hydroxyphenyl)propionic acidb | 1.1/15/44 | 91 ± 49 | 98 ± 7 | 95 ± 9 | 42/8/3 |
| 3-Hydroxyphenylacetic acid | 27/360/1100 | 93 ± 23 | 104 ± 10 | 96 ± 9 | 46/5/3 |
| 4-Hydroxyphenylacetic acid | 34/460/1400 | 101 ± 22 | 89 ± 17 | 92 ± 19 | 22/9/6 |
| Homovanillic acid | 9.9/130/400 | 97 ± 23 | 97 ± 9 | 96 ± 12 | 28/5/5 |
| Homoprotocatechuic acid | 5.5/73/220 | 88 ± 17 | 97 ± 8 | 93 ± 8 | 15/4/2 |
| ***Lignans*** |  |  |  |  |  |
| Enterodiol | 0.064/0.85/2.6 | 87 ± 36 | 87 ± 8 | 91 ± 8 | 23/7/6 |
| Enterolactone | 0.2/2.7/8.1 | 84 ± 13 | 90 ± 13 | 92 ± 15 | 16/5/4 |
| ***Others*** |  |  |  |  |  |
| 2,6-Dimethoxyphenol | 0.62/8.3/25 | 100 ± 13 | 100 ± 11 | 95 ± 14 | 13/14/14 |
| 3,5-Dimethoxy-4 -hydroxyphenylacetic acid^b^ | 4.3/58/170 | 94 ± 14 | 102 ± 10 | 94 ± 12 | 19/7/4 |
| 3-Methylcatechol | 2/27/80 | 90 ± 11 | 96 ± 6 | 95 ± 11 | 12/4/3 |
| 4-Methylcatechol | 4.4/59/180 | 92 ± 7 | 94 ± 6 | 95 ± 10 | 9/5/3 |
| Catecholb | 13/170/520 | 85 ± 45 | 53 ± 36 | 34 ± 53 | 68/59/38 |
| Eugenol | 30/390/1200 | 87 ± 24 | 93 ± 15 | 92 ± 13 | 26/12/8 |
| Hydroxytyrosol | 0.56/7.5/23 | 87 ± 9 | 91 ± 5 | 91 ± 8 | 13/5/2 |
| Pyrogallol | 5.8/77/230 | 92 ± 33 | 92 ± 9 | 89 ± 13 | 45/23/14 |
| Thymol | 8.9/120/360 | n.d. | n.d. | 76 ± 32 | n.d./n.d./44 |
| Urolithin A | 0.11/1.5/4.4 | 87 ± 20 | 90 ± 6 | 92 ± 10 | 16/7/4 |
| ***Stilbenes*** |  |  |  |  |  |
| Dihydroresveratrol | 0.47/6.2/19 | 88 ± 17 | 92 ± 11 | 93 ± 13 | 18/5/8 |
| Polydatin | 0.15/2.1/6.2 | 121 ± 38 | 88 ± 21 | 87 ± 13 | 60/10/7 |
| Pterostilbeneb | 0.21/2.9/8.6 | 99 ± 19 | 91 ± 11 | 91 ± 10 | 26/8/7 |
| **Analyte** | **Spiking Level (LL/ML/HL)**  **(ng*mL^-1^)** | **R_E_ ± RSD_R_ (LL) (%)** | **R_E_ ± RSD_R_ (ML) (%)** | **R_E_ ± RSD_R_ (HL) (%)** | **RSD_r_**  **(LL/ML/HL)**  **(%)** |
| Resveratrol | 0.42/5.6/17 | 86 ± 15 | 92 ± 8 | 89 ± 11 | 24/4/4 |
| ***Anthocyanins*** |  |  |  |  |  |
| Cyanidin | 59/780/2400 | n.d. | 43 ± 15 | 48 ± 15 | n.d./39/43 |
| Cyanidin-3-O-glucoside | 0.81/11/32 | n.d. | 16 ± 13 | 14 ± 14 | n.d./20/6 |
| Cyanidin-3-O-rutinoside | 0.69/9.2/28 | n.d. | 20 ± 18 | 21 ± 20 | n.d./22/9 |
| Cyanidin-3-O-sambubioside | 1.5/20/61 | n.d. | 7 ± 28 | 6 ± 19 | n.d./37/13 |
| Delphinidin | 70/940/2800 | n.d. | 23 ± 17 | 22 ± 13 | n.d./35/36 |
| Delphinidin-3-O-glucoside | 30/400/1200 | n.d. | 11 ± 31 | 8 ± 16 | n.d./43/13 |
| ***Catechins*** |  |  |  |  |  |
| (-)- Epicatechin | 2.7/36/110 | 94 ± 12 | 85 ± 9 | 91 ± 10 | 20/10/8 |
| (-)-Epicatechin gallate | 1.1/14/43 | 90 ± 17 | 65 ± 12 | 66 ± 8 | 32/5/6 |
| (-)-Epigallocatechin | 19/250/740 | 101 ± 37 | 89 ± 9 | 87 ± 14 | 46/27/11 |
| (-)-Epigallocatechin gallate | 13/180/530 | 123 ± 31 | 50 ± 19 | 49 ± 18 | 22/14/13 |
| (-)-Gallocatechin | 19/250/740 | 90 ± 35 | 84 ± 9 | 84 ± 12 | 87/17/12 |
| (+)-Catechin | 1.6/21/64 | 85 ± 16 | 78 ± 5 | 85 ± 13 | 16/4/6 |
| ***Flavanones*** |  |  |  |  |  |
| (+/-)-Naringeninb | 0.11/1.4/4.3 | 78 ± 50 | 88 ± 11 | 91 ± 16 | 43/6/3 |
| 8-Prenylnaringenin | 0.26/3.5/11 | 93 ± 8 | 95 ± 6 | 92 ± 11 | 4/4/3 |
| Hesperetinb | 0.11/1.5/4.6 | 96 ± 16 | 91 ± 10 | 92 ± 8 | 10/6/5 |
| Hesperidin | 0.079/1.1/3.2 | 99 ± 15 | 94 ± 10 | 95 ± 7 | 14/7/12 |
| Isoxanthohumolb | 0.054/0.73/2.2 | 96 ± 13 | 92 ± 6 | 92 ± 7 | 19/5/4 |
| Naringin | 3/40/120 | 85 ± 28 | 86 ± 14 | 80 ± 17 | 16/13/14 |
| Neohesperidin | 4/54/160 | 86 ± 22 | 84 ± 8 | 84 ± 9 | 14/11/6 |
| Neohesperidin dihydrochalcone | 0.079/1/3.1 | 86 ± 16 | 82 ± 15 | 87 ± 12 | 9/9/9 |
| Xanthohumolb | 0.16/2.1/6.3 | 92 ± 14 | 91 ± 10 | 90 ± 8 | 8/5/3 |
| ***Flavones*** |  |  |  |  |  |
| Apigenin | 0.12/1.6/4.7 | 87 ± 6 | 86 ± 9 | 94 ± 11 | 6/6/3 |
| **Analyte** | **Spiking Level (LL/ML/HL)**  **(ng*mL^-1^)** | **R_E_ ± RSD_R_ (LL) (%)** | **R_E_ ± RSD_R_ (ML) (%)** | **R_E_ ± RSD_R_ (HL) (%)** | **RSD_r_**  **(LL/ML/HL)**  **(%)** |
| Diosmetin | 0.086/1.2/3.5 | 92 ± 25 | 92 ± 9 | 95 ± 11 | 7/6/3 |
| Diosmin | 0.34/4.5/14 | 120 ± 20 | 93 ± 20 | 79 ± 21 | 17/18/12 |
| ***Flavonols*** |  |  |  |  |  |
| (+)-Rutinb | 0.39/5.2/16 | 78 ± 13 | 61 ± 9 | 62 ± 12 | 12/12/8 |
| Isorhamnetin | 0.079/1.1/3.2 | 95 ± 15 | 71 ± 5 | 72 ± 10 | 28/5/2 |
| Kaempferol | 1.8/24/72 | 93 ± 13 | 90 ± 10 | 88 ± 13 | 8/8/8 |
| Kaempferol-3-O-glucuronide | 0.1/1.4/4.2 | 91 ± 18 | 85 ± 9 | 84 ± 16 | 21/6/11 |
| Quercetinb | 0.71/9.5/28 | 97 ± 15 | 47 ± 12 | 44 ± 13 | 15/11/11 |
| Quercetin-7-O-β-D-glucuronide | 0.43/5.8/17 | 80 ± 22 | 55 ± 21 | 51 ± 20 | 34/13/11 |
| ***Isoflavones*** |  |  |  |  |  |
| Biochanin A | 0.12/1.6/4.7 | 88 ± 18 | 91 ± 5 | 91 ± 8 | 12/4/3 |
| Daidzein | 0.15/2/5.9 | 84 ± 35 | 91 ± 7 | 93 ± 9 | 11/5/4 |
| Daidzein-7-β-D-glucuronide | 0.43/5.7/17 | 88 ± 22 | 88 ± 9 | 88 ± 10 | 16/9/3 |
| Genistein | 0.13/1.7/5.1 | 78 ± 6 | 65 ± 16 | 93 ± 17 | 5/7/5 |
| Genistein-7-β-D-glucuronide | 0.48/6.5/19 | 94 ± 15 | 91 ± 13 | 90 ± 16 | 12/10/5 |
| Genistein-7-sulfateb | 3.8/51/150 | 89 ± 7 | 59 ± 23 | 30 ± 62 | 6/4/3 |
| S-Equol | 5.8/77/230 | 77 ± 61 | 94 ± 13 | 92 ± 12 | 40/11/6 |
| ***Proanthocyanidins*** |  |  |  |  |  |
| Procyanidin A2 | 0.93/12/37 | 94 ± 34 | 78 ± 13 | 73 ± 11 | 38/14/17 |
| Procyanidin B1 | 15/200/590 | 60 ± 16 | 71 ± 7 | 71 ± 8 | 12/4/3 |
| Procyanidin B2 | 3.9/53/160 | n.d. | 70 ± 56 | 68 ± 22 | n.d./21/8 |
| Procyanidin C1 | 2.8/37/110 | 80 ± 31 | 53 ± 12 | 48 ± 12 | 51/12/6 |

**Table S5.** Method validation outcomes, the analytes where all figures of merit fit the validation criteria are check marked, while for the others the criteria that are out of acceptable range are listed. Evaluated figures of merit are regression coefficient (R²), recovery (R_E_), intermediate precision (RSD_R_) and repeatability (RSD_r_) at the low (LL), middle (ML) and high (HL) spiking level.

| **Analyte** | **Validation Outcome** |
| --- | --- |
| ***Dihydrochalcones*** |  |
| Phloretin | ✓ |
| ***Hydroxybenzoic Acids*** |  |
| 3,5-Dihydroxybenzoic acid | R_E_ LL/ML/HL, RSD_R_ LL, RSD_r_ LL/ML/HL |
| 3-Hydroxybenzoic acid | ✓ |
| 4-Hydroxybenzoic acid | ✓ |
| Benzoic acid | R_E_ LL, RSD_R_ LL, RSD_r_ LL |
| Ellagic acid | R_E_ LL/ML/HL, RSD_R_ LL, RSD_r_ LL |
| Ethyl gallate | ✓ |
| Gallic acid | R_E_ LL/ML/HL, RSD_R_ LL, RSD_r_ LL/HL |
| Protocatechuic acid | R_E_ LL/ML/HL, RSD_R_ LL, RSD_r_ LL/ML/HL, R^2^ |
| Salicylic acid | ✓ |
| Syringic acid | ✓ |
| Vanillic acid | ✓ |
| ***Hydroxycinnamic Acids*** |  |
| Caffeic acid | ✓ |
| Caffeic acid-3-β-D-glucuronide | ✓ |
| Chlorogenic acid | ✓ |
| Cinnamic acid | ✓ |
| Dihydrocaffeic acid | ✓ |
| Dihydroferulic acid | ✓ |
| Ferulic acid/Isoferulic acid | ✓ |
| p-Coumaric acid | ✓ |
| Sinapic acid | ✓ |
| trans-m-Coumaric acid | ✓ |
| trans-o-Coumaric acid | ✓ |
| ***Hydroxyphenylacetic Acids*** |  |
| 3-(3-Hydroxyphenyl)propionic acid | RSD_R_ LL, RSD_r_ LL |
| 3-Hydroxyphenylacetic acid | RSD_r_ LL |
| 4-Hydroxyphenylacetic acid | ✓ |
| Homovanillic acid | ✓ |
| Homoprotocatechuic acid | ✓ |
| ***Lignans*** |  |
| Enterodiol | ✓ |
| **Analyte** | **Validation Outcome** |
| Enterolactone | ✓ |
| ***Others*** |  |
| 2,6-Dimethoxyphenol | ✓ |
| 3,5-Dimethoxy-4-hydroxyphenylacetic acid | ✓ |
| 3-Methylcatechol | ✓ |
| 4-Methylcatechol | ✓ |
| Catechol | R_E_ ML/HL, RSD_R_ LL/ML/HL, RSD_r_ LL/ML/HL, R^2^ |
| Eugenol | ✓ |
| Hydroxytyrosol | ✓ |
| Pyrogallol | R_E_ LL, RSD_r_ LL |
| Thymol | R_E_ LL/ML, RSD_R_ LL/ML/HL, RSD_r_ LL/ML/HL, R^2^ |
| Urolithin A | ✓ |
| ***Stilbenes*** |  |
| Dihydroresveratrol | ✓ |
| Polydatin | RSD_r_ LL |
| Pterostilbene | ✓ |
| Resveratrol | ✓ |
| ***Anthocyanins*** |  |
| Cyanidin | R_E_ LL/ML/HL, RSD_R_ LL, RSD_r_ LL/ML/HL |
| Cyanidin-3-O-glucoside | R_E_ LL/ML/HL, RSD_R_ LL, RSD_r_ LL |
| Cyanidin-3-O-rutinoside | R_E_ LL/ML/HL, RSD_R_ LL, RSD_r_ LL |
| Cyanidin-3-O-sambubioside | R_E_ LL/ML/HL, RSD_R_ LL, RSD_r_ LL/ML |
| Delphinidin | R_E_ LL/ML/HL, RSD_R_ LL, RSD_r_ LL |
| Delphinidin-3-O-glucoside | R_E_ LL/ML/HL, RSD_R_ LL/ML, RSD_r_ LL/ML |
| ***Catechins*** |  |
| (-)-Epicatechin | ✓ |
| (-)-Epicatechin gallate | ✓ |
| (-)-Epigallocatechin | R_E_ LL, RSD_r_ LL |
| (-)-Epigallocatechin gallate | R_E_ LL |
| (-)-Gallocatechin | R_E_ LL, RSD_r_ LL |
| (+)-Catechin | ✓ |
| ***Flavanones*** |  |
| (+/-)-Naringenin | RSD_R_ LL, RSD_r_ LL |
| 8-Prenylnaringenin | ✓ |
| Hesperetin | ✓ |
| Hesperidin | ✓ |
| Isoxanthohumol | ✓ |
| **Analyte** | **Validation Outcome** |
| Naringin | ✓ |
| Neohesperidin | ✓ |
| Neohesperidin dihydrochalcone | ✓ |
| Xanthohumol | ✓ |
| ***Flavones*** |  |
| Apigenin | ✓ |
| Diosmetin | ✓ |
| Diosmin | ✓ |
| ***Flavonols*** |  |
| (+)-Rutin | ✓ |
| Isorhamnetin | ✓ |
| Kaempferol | ✓ |
| Kaempferol-3-O-glucuronide | ✓ |
| Quercetin | R_E_ HL |
| Quercetin-7-O-β-D-glucuronide | ✓ |
| ***Isoflavones*** |  |
| Biochanin A | ✓ |
| Daidzein | ✓ |
| Daidzein-7-β-D-glucuronide | ✓ |
| Genistein | ✓ |
| Genistein-7-β-D-glucuronide | ✓ |
| Genistein-7-sulfate | RSD_R_ HL, R^2^ |
| S-Equol | RSD_R_ LL, RSD_r_ LL |
| ***Proanthocyanidins*** |  |
| Procyanidin A2 | ✓ |
| Procyanidin B1 | ✓ |
| Procyanidin B2 | R_E_ LL, RSD_R_ LL/ML, RSD_r_ LL |
| Procyanidin C1 | R_E_ HL, RSD_r_ LL |


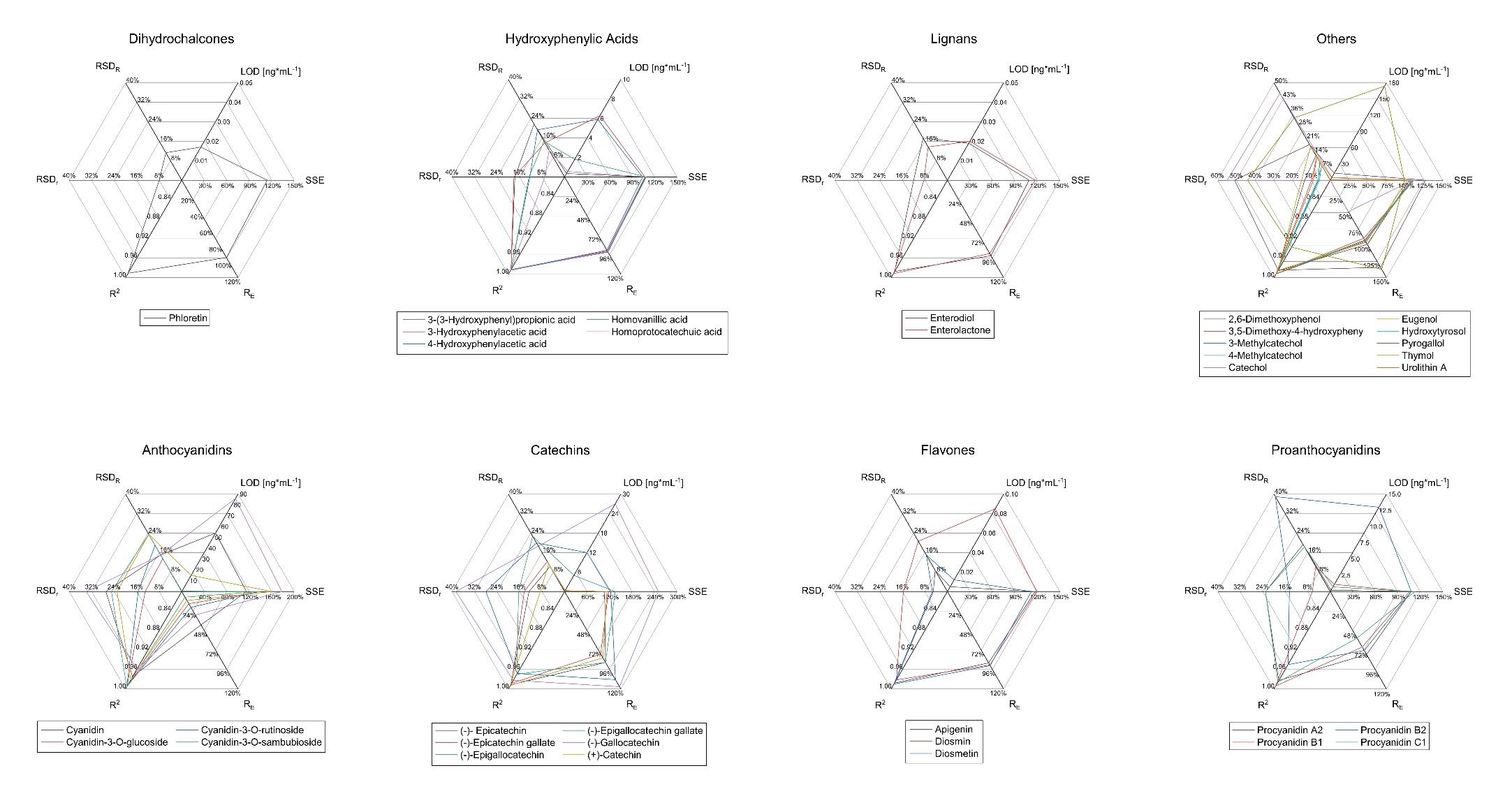


**Figure S2.** Analytical figures of merit evaluated during the method validation for remaining eight polyphenol classes (see Figure 1). The limit of quantification (LOQ), regression coefficient (R²), average recovery (R^E^), average intermediate precision (RSD_R_) and average repeatability (RSD_r_) are displayed.

**Table S6.** Estimated daily polyphenol intake calculated for each breast milk sample (S1-S10) from one month after birth and calculated mean and median values. All values are given in microgram analyte per kilogram infant bodyweight per day (µg*kg^-1^ bw day^-1^).

| **Analyte** | **Mean** | **Median** | | **S1** | | **S2** | | **S3** | | **S4** | | **S5** | | **S6** | | **S7** | | **S8** | | **S9** | | **S10** | |
| --- | --- | --- | --- | --- | --- | --- | --- | --- | --- | --- | --- | --- | --- | --- | --- | --- | --- | --- | --- | --- | --- | --- | --- |
| ***Dihydrochalcones*** |  |  |  | |  | |  | |  | |  | |  | |  | |  | |  | |  | |  |
| Phloretin | 0.0044 | 0.0044 | - | | - | | - | | - | | - | | - | | - | | - | | 0.0044 | | - | |  |
| ***Hydroxybenzoic Acids*** |  |  |  | |  | |  | |  | |  | |  | |  | |  | |  | |  | |  |
| 3,5-Dihydroxybenzoic acid | 8.6 | 0.34 | - | | - | | 0.42 | | 4.1 | | 0.1 | | 47 | | 0.25 | | - | | 0.1 | | - | |  |
| 3-Hydroxybenzoic acid | 0.46 | 0.44 | - | | - | | 0.44 | | 0.53 | | 0.25 | | 0.88 | | 0.23 | | - | | 0.66 | | 0.21 | |  |
| 4-Hydroxybenzoic acid | 7.5 | 1.9 | 0.91 | | 0.83 | | 6 | | 11 | | 1.1 | | 48 | | 0.94 | | 1.4 | | 2.4 | | 2.3 | |  |
| Gallic acid | 0.032 | 0.032 | - | | - | | - | | - | | - | | 0.032 | | - | | - | | - | | - | |  |
| Protocatechuic acid | 0.72 | 0.024 | 0.015 | | - | | 0.038 | | 0.39 | | - | | 4.5 | | 0.024 | | - | | 0.015 | | 0.015 | |  |
| Salicylic acid | 11 | 0.54 | 1.1 | | 0.32 | | 0.51 | | 28 | | 0.5 | | 42 | | 36 | | 0.16 | | 0.49 | | 0.56 | |  |
| Vanillic acid | 0.14 | 0.15 | 0.056 | | - | | 0.11 | | 0.2 | | 0.087 | | 0.21 | | 0.19 | | - | | - | | - | |  |
| ***Hydroxycinnamic Acids*** |  |  |  | |  | |  | |  | |  | |  | |  | |  | |  | |  | |  |
| Chlorogenic acid | 0.68 | 0.67 | - | | 1 | | - | | - | | 0.34 | | - | | - | | - | | - | | - | |  |
| Dihydrocaffeic acid | 1.3 | 0.16 | - | | - | | - | | 0.16 | | - | | 3.8 | | 0.041 | | - | | - | | - | |  |
| Dihydroferulic acid | 0.49 | 0.50 | 0.58 | | - | | - | | - | | - | | 0.41 | | - | | - | | - | | - | |  |
| Ferulic acid/Isoferulic acid | 0.12 | 0.094 | 0.046 | | 0.091 | | 0.39 | | 0.06 | | 0.096 | | 0.21 | | 0.026 | | 0.11 | | 0.076 | | 0.11 | |  |
| p-Coumaric acid | 1.1 | 0.79 | - | | - | | 0.08 | | 1.9 | | - | | 1.5 | | 2.8 | | 0.012 | | - | | 0.012 | |  |
| Sinapic acid | 0.021 | 0.021 | - | | 0.021 | | - | | - | | - | | - | | - | | - | | - | | - | |  |
| ***Hydroxyphenylacetic Acids*** |  |  |  | |  | |  | |  | |  | |  | |  | |  | |  | |  | |  |
| 3-(3-Hydroxyphenyl)propionic acid | 0.2 | 0.195 | 0.27 | | 0.095 | | - | | - | | 0.12 | | - | | - | | - | | - | | 0.31 | |  |
| 4-Hydroxyphenylacetic acid | 180 | 5.5 | 1.5 | | - | | 58 | | 19 | | 2.9 | | 1500 | | 5.5 | | 1.5 | | 47 | | 2 | |  |
| Homovanillic acid | 0.64 | 0.66 | - | | - | | 0.81 | | - | | - | | 0.46 | | - | | - | | - | | 0.66 | |  |
| Homoprotocatechuic acid | 7.8 | 7.8 | - | | - | | - | | - | | - | | 7.8 | | - | | - | | - | | - | |  |
| ***Lignans*** |  |  |  | |  | |  | |  | |  | |  | |  | |  | |  | |  | |  |
| Enterodiol | 0.054 | 0.054 | - | | - | | - | | - | | - | | 0.091 | | - | | - | | 0.017 | | - | |  |
| **Analyte** | **Mean** | **Median** | **S1** | | **S2** | | **S3** | | **S4** | | **S5** | | **S6** | | **S7** | | **S8** | | **S9** | | **S10** | |  |
| Enterolactone | 0.099 | 0.031 | - | | - | | 0.03 | | 0.24 | | - | | - | | - | | - | | 0.031 | | - | |  |
| ***Others*** |  |  |  | |  | |  | |  | |  | |  | |  | |  | |  | |  | |  |
| 2,6-Dimethoxyphenol | 0.04 | 0.04 | - | | - | | - | | - | | - | | 0.04 | | - | | - | | - | | - | |  |
| 3,5-Dimethoxy-4-hydroxyphenylacetic acid | 1.7 | 1.7 | - | | - | | - | | 0.66 | | - | | 2.8 | | - | | - | | - | | - | |  |
| 4-Methylcatechol | 1.4 | 1.4 | - | | - | | - | | - | | - | | 1.4 | | - | | - | | - | | - | |  |
| Catechol | 89 | 31 | 13 | | 1.3 | | - | | 160 | | 1.3 | | 290 | | - | | - | | 31 | | 120 | |  |
| Hydroxytyrosol | 1.5 | 1.5 | - | | - | | - | | - | | - | | 2.9 | | - | | - | | 0.019 | | - | |  |
| Pyrogallol | 0.47 | 0.47 | 0.5 | | 0.44 | | - | | - | | - | | - | | - | | - | | - | | - | |  |
| ***Flavanones*** |  |  |  | |  | |  | |  | |  | |  | |  | |  | |  | |  | |  |
| (+/-)-Naringenin | 0.27 | 0.14 | 0.012 | | 0.009 | | 0.24 | | 0.24 | | - | | 0.17 | | 0.14 | | 0.013 | | 1.6 | | 0.043 | |  |
| 8-Prenylnaringenin | 0.16 | 0.16 | - | | - | | - | | - | | - | | - | | - | | - | | 0.16 | | - | |  |
| Hesperetin | 0.17 | 0.17 | - | | - | | - | | - | | - | | 0.29 | | 0.052 | | - | | - | | - | |  |
| ***Flavones*** |  |  |  | |  | |  | |  | |  | |  | |  | |  | |  | |  | |  |
| Apigenin | 0.093 | 0.037 | - | | - | | 0.009 | | 0.037 | | - | | 0.23 | | - | | - | | - | | - | |  |
| Diosmetin | 0.055 | 0.022 | 0.0038 | | 0.011 | | 0.04 | | 0.1 | | - | | 0.2 | | - | | - | | 0.022 | | 0.0088 | |  |
| ***Flavonols*** |  |  |  | |  | |  | |  | |  | |  | |  | |  | |  | |  | |  |
| Isorhamnetin | 0.0073 | 0.0045 | 0.0023 | | - | | - | | 0.015 | | - | | 0.0045 | | - | | - | | - | | - | |  |
| Kaempferol | 0.043 | 0.043 | - | | - | | - | | 0.043 | | - | | - | | - | | - | | 0.043 | | - | |  |
| Kaempferol-3-O-glucuronide | 0.1 | 0.1 | - | | 0.1 | | - | | - | | - | | - | | - | | - | | - | | - | |  |
| ***Isoflavones*** |  |  |  | |  | |  | |  | |  | |  | |  | |  | |  | |  | |  |
| Daidzein | 0.26 | 0.066 | - | | - | | 0.0085 | | 1.1 | | - | | 0.066 | | 0.099 | | - | | - | | 0.0085 | |  |
| Genistein | 0.043 | 0.018 | - | | - | | 0.018 | | 0.14 | | - | | 0.014 | | 0.026 | | - | | - | | 0.013 | |  |
| Genistein-7-sulfate | 0.045 | 0.045 | - | | - | | - | | 0.045 | | - | | - | | 0.045 | | 0.045 | | - | | 0.045 | |  |
| ***Proanthocyanidins*** |  |  |  | |  | |  | |  | |  | |  | |  | |  | |  | |  | |  |
| Procyanidin C1 | 0.15 | 0.15 | 0.16 | | - | | 0.15 | | - | | 0.15 | | 0.15 | | - | | - | | - | | - | |  |

**Table S7.** Calibration curves for each analyte, with the analyte concentration [ng*mL^-1^] on the x-axis and the peak area on the y-axis.

| Catechol 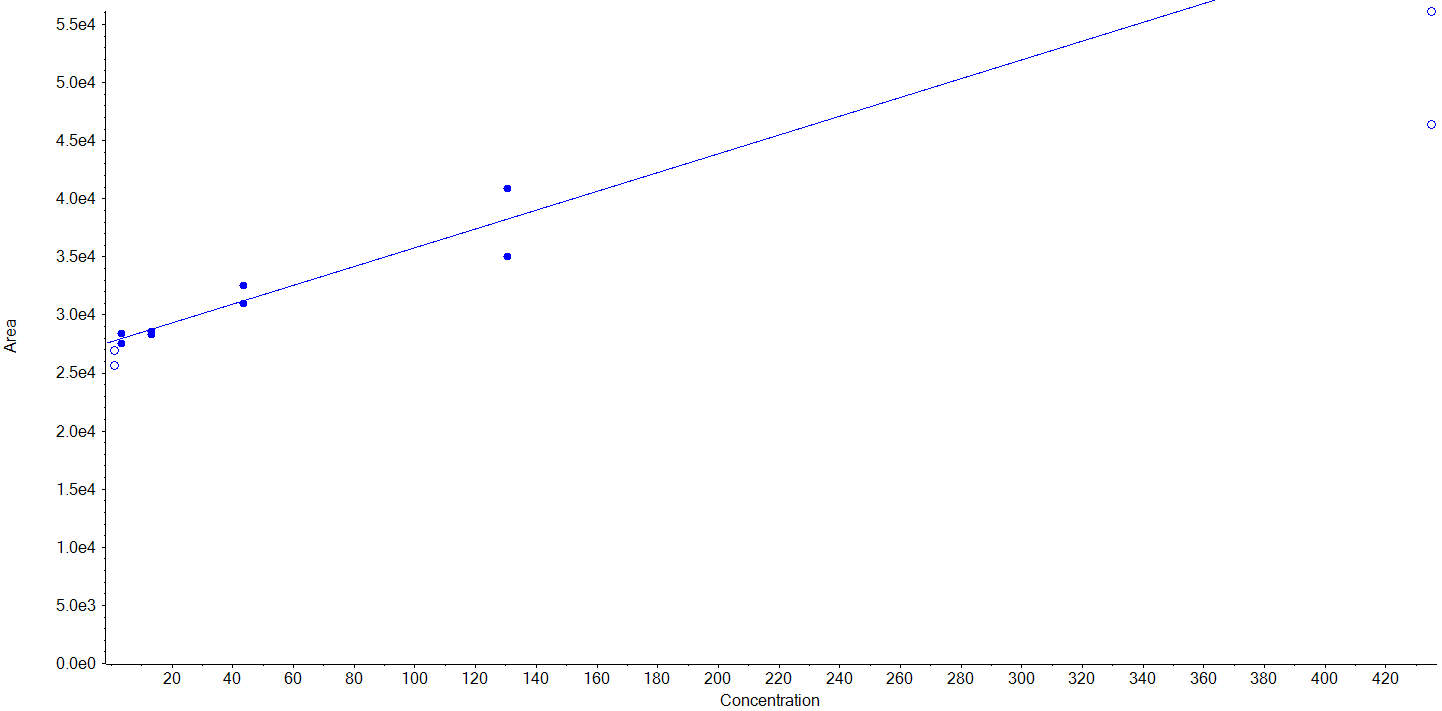 | Benzoic acid 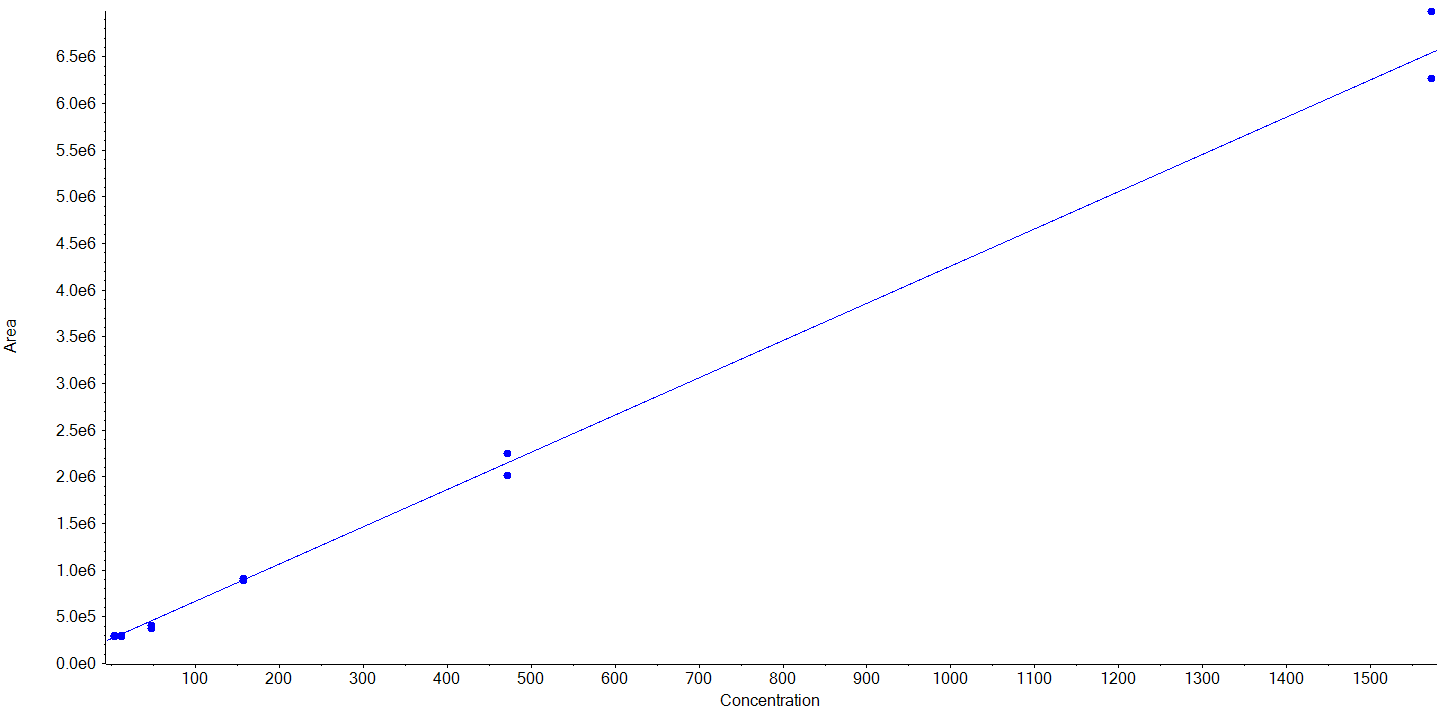 | 4-Methylcatechol 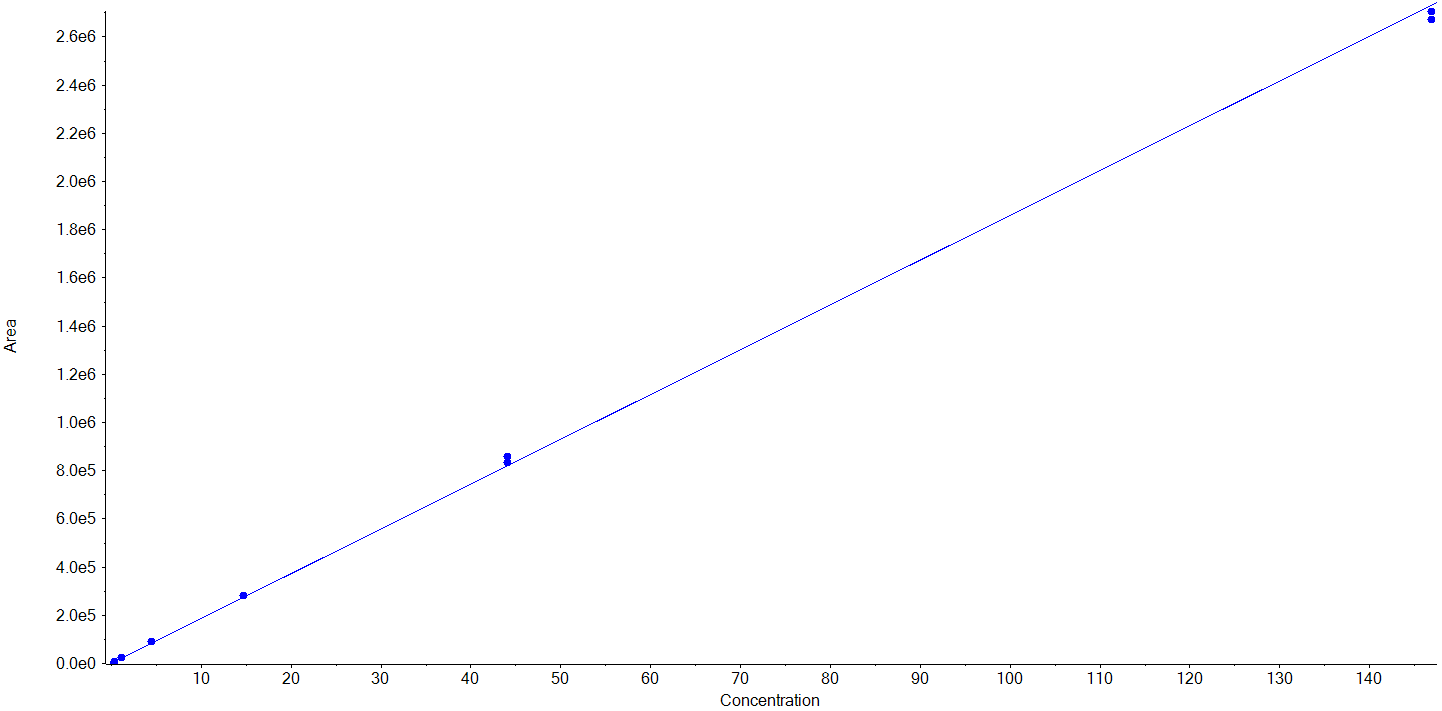 |
| --- | --- | --- |
| 4-Hydroxybenzoic acid 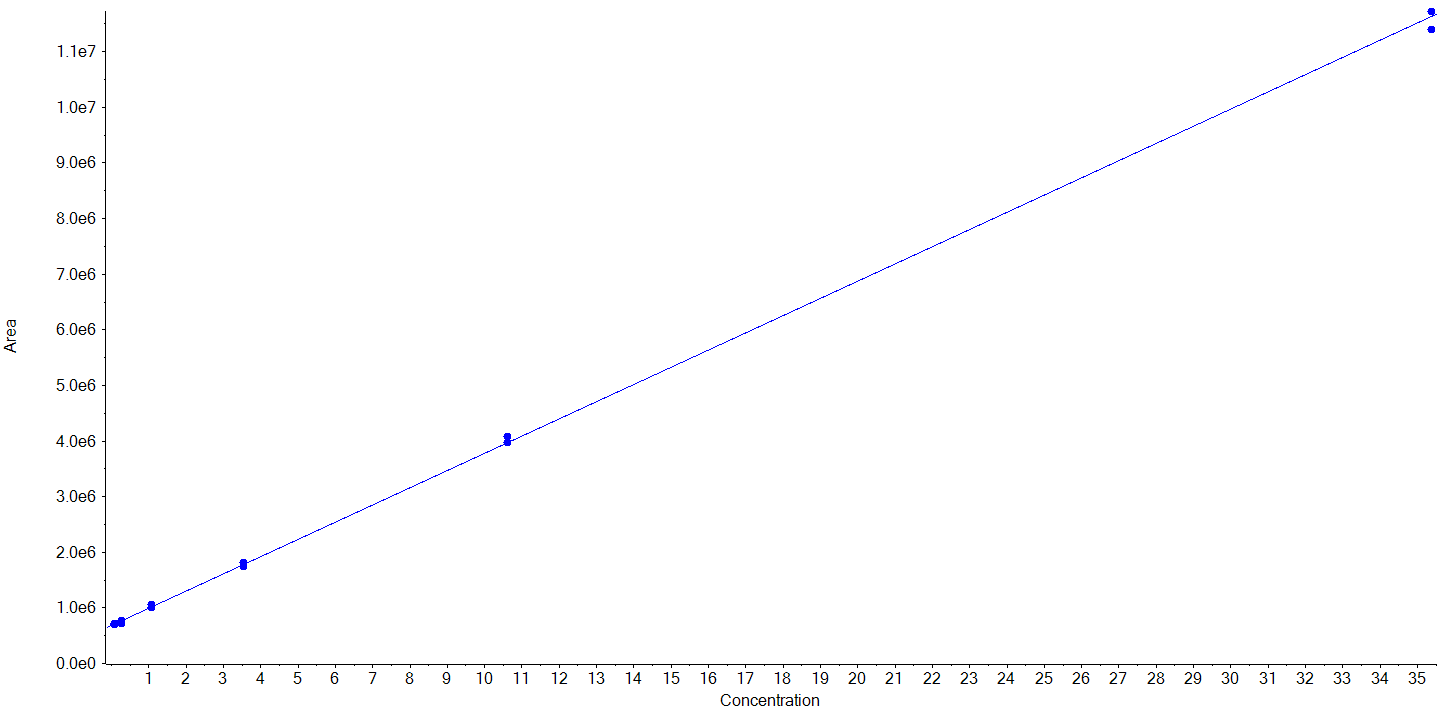 | Salicylic acid 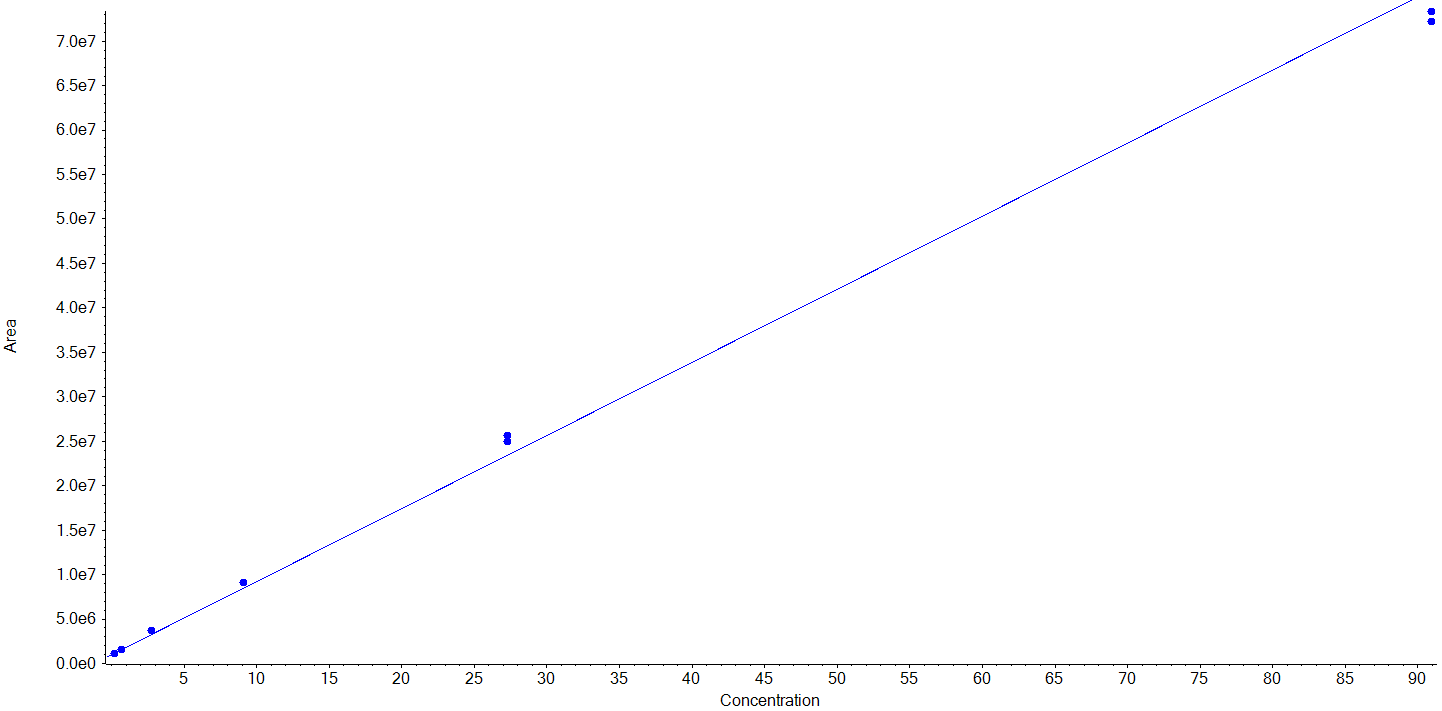 | Cinnamic acid 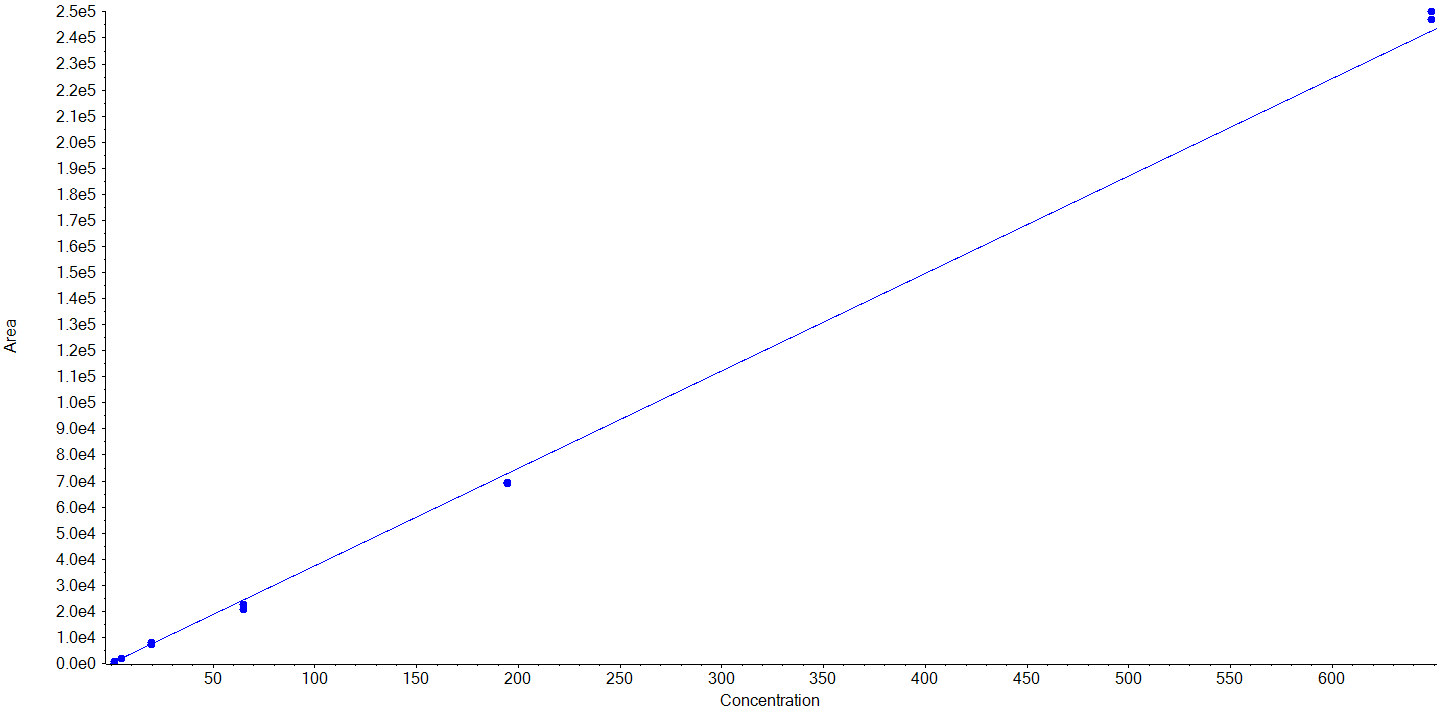 |
| 3-Methylcatechol 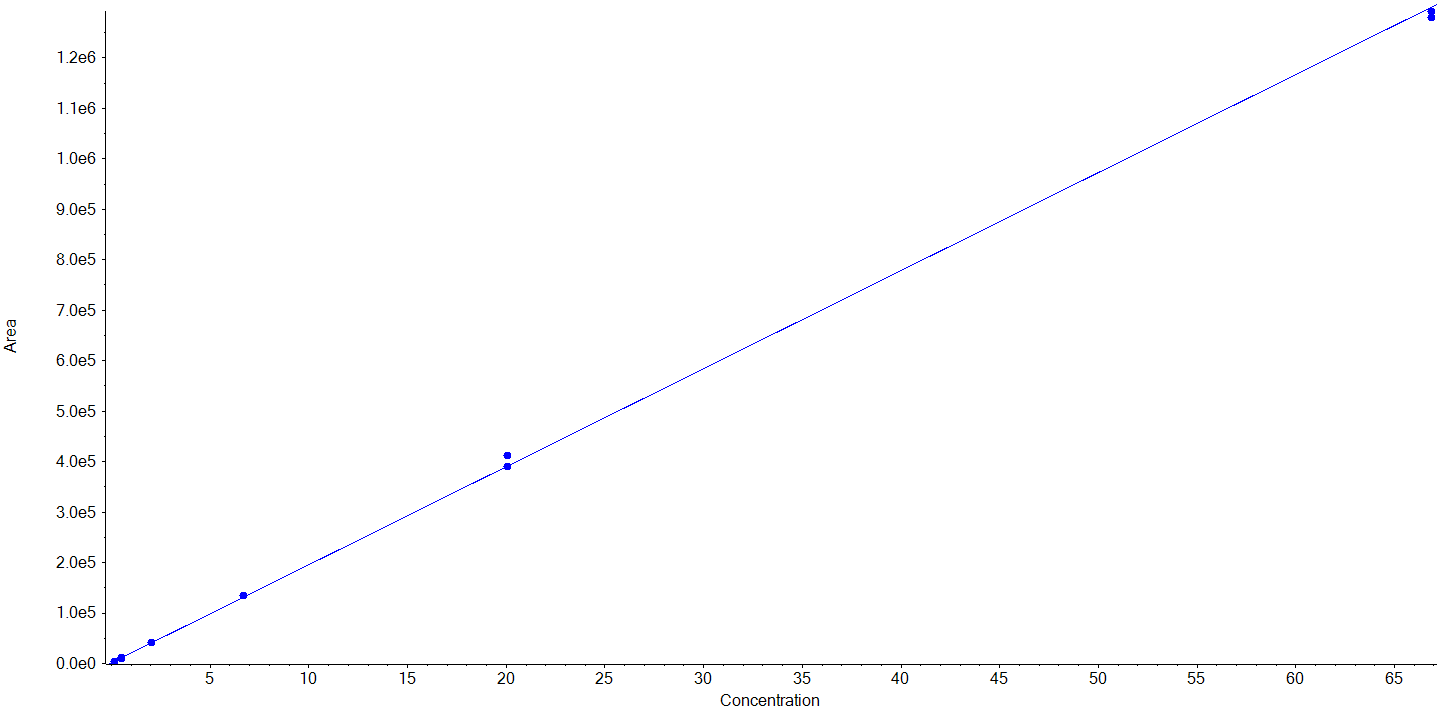 | Pyrogallol 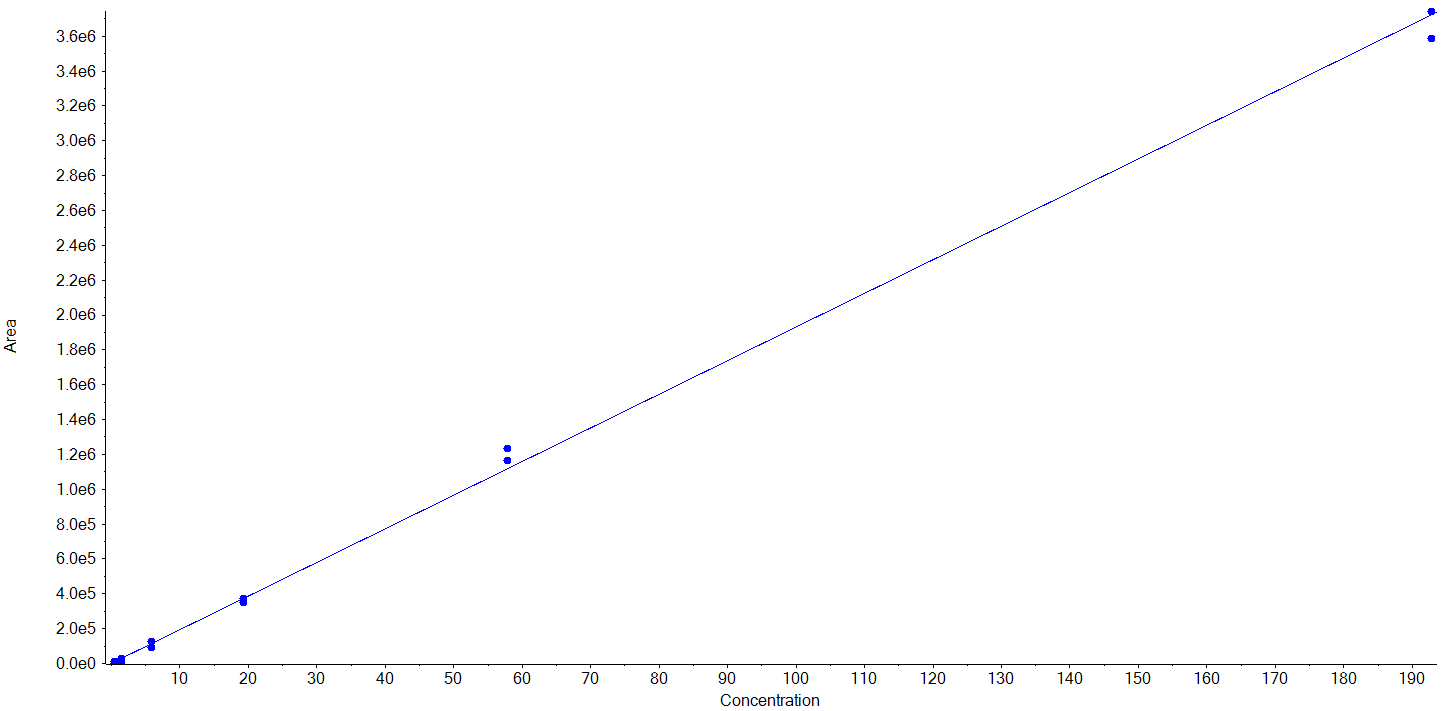 | 3-Hydroxybenzoic acid 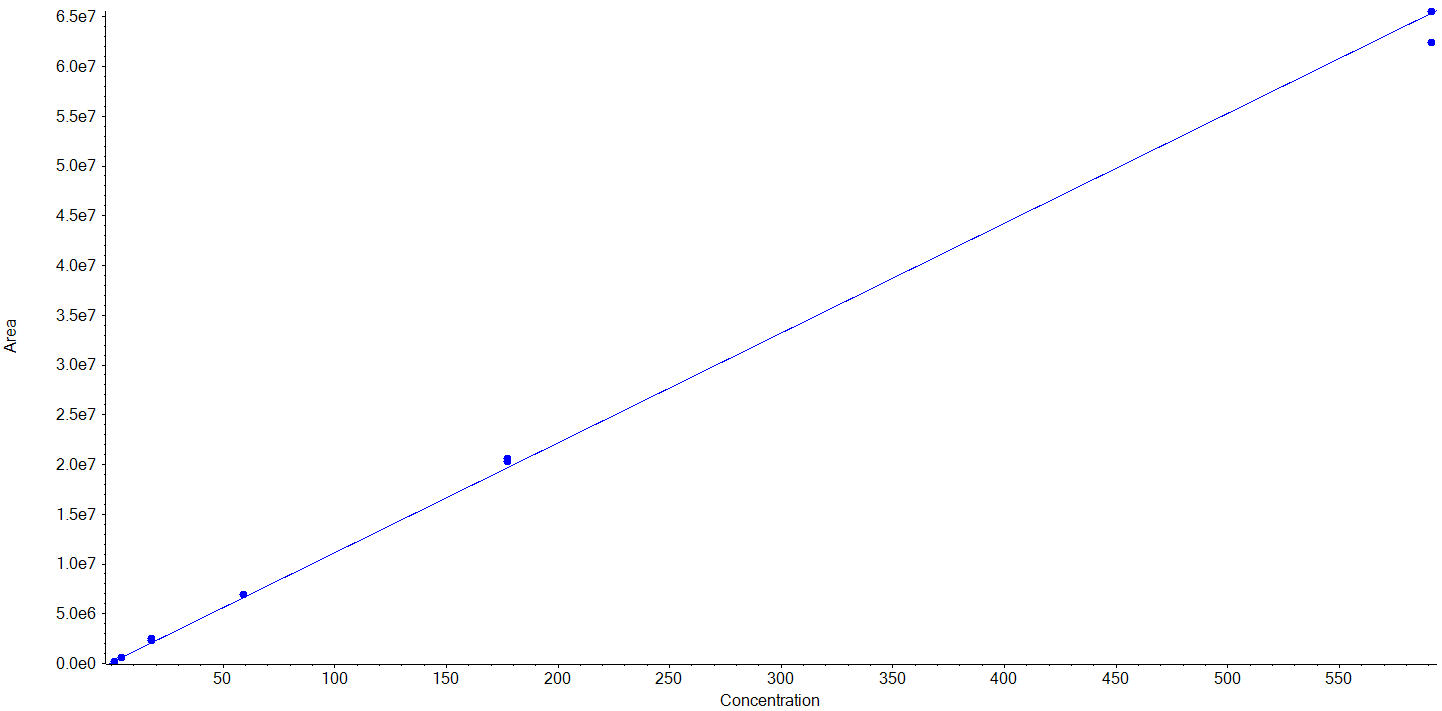 |
| Thymol 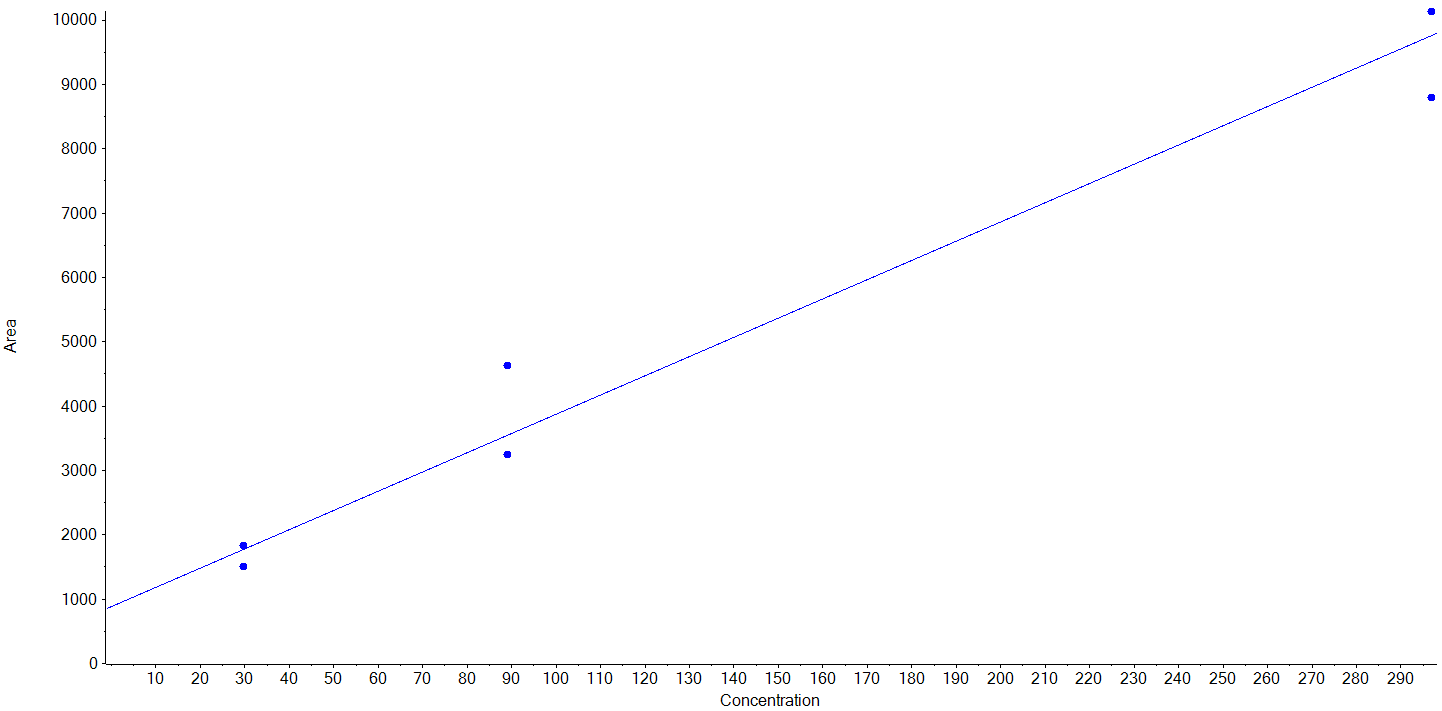 | 4-Hydroxyphenylacetic acid 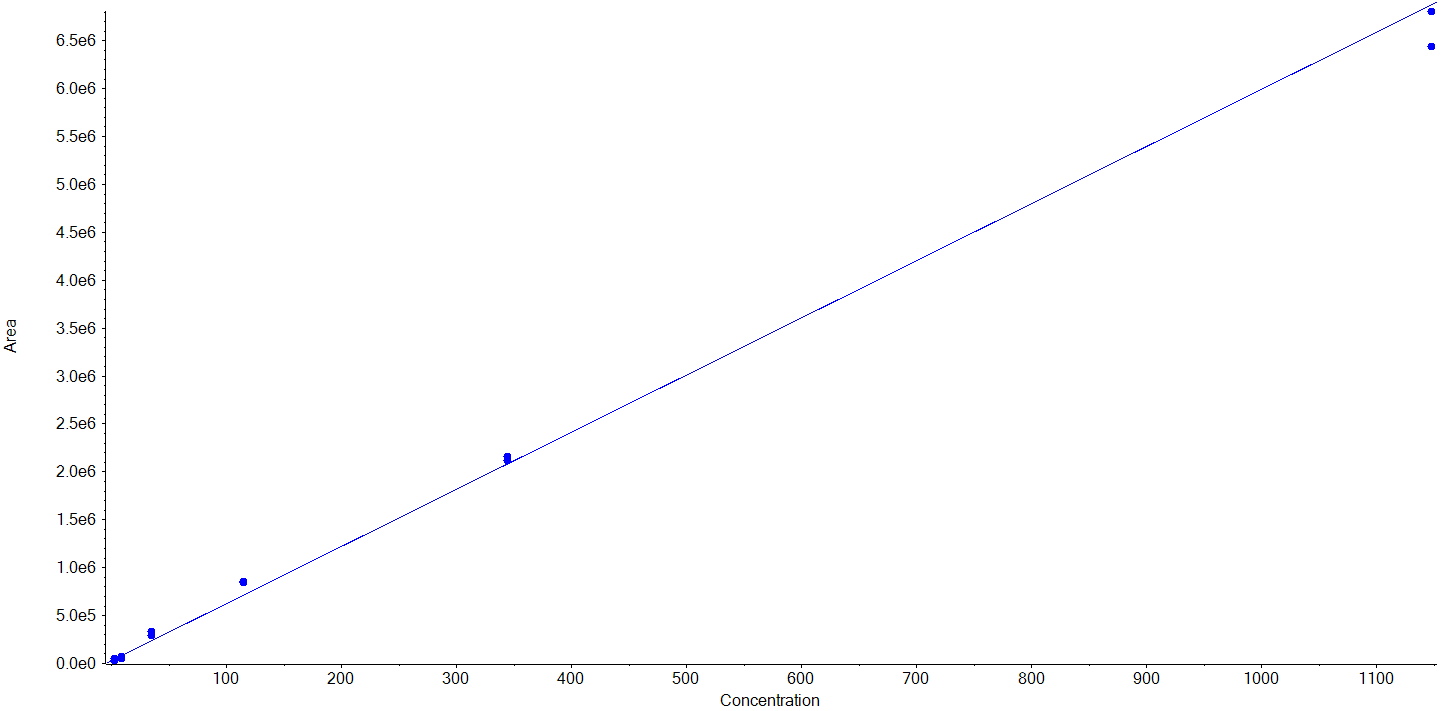 | 3-Hydroxyphenylacetic acid 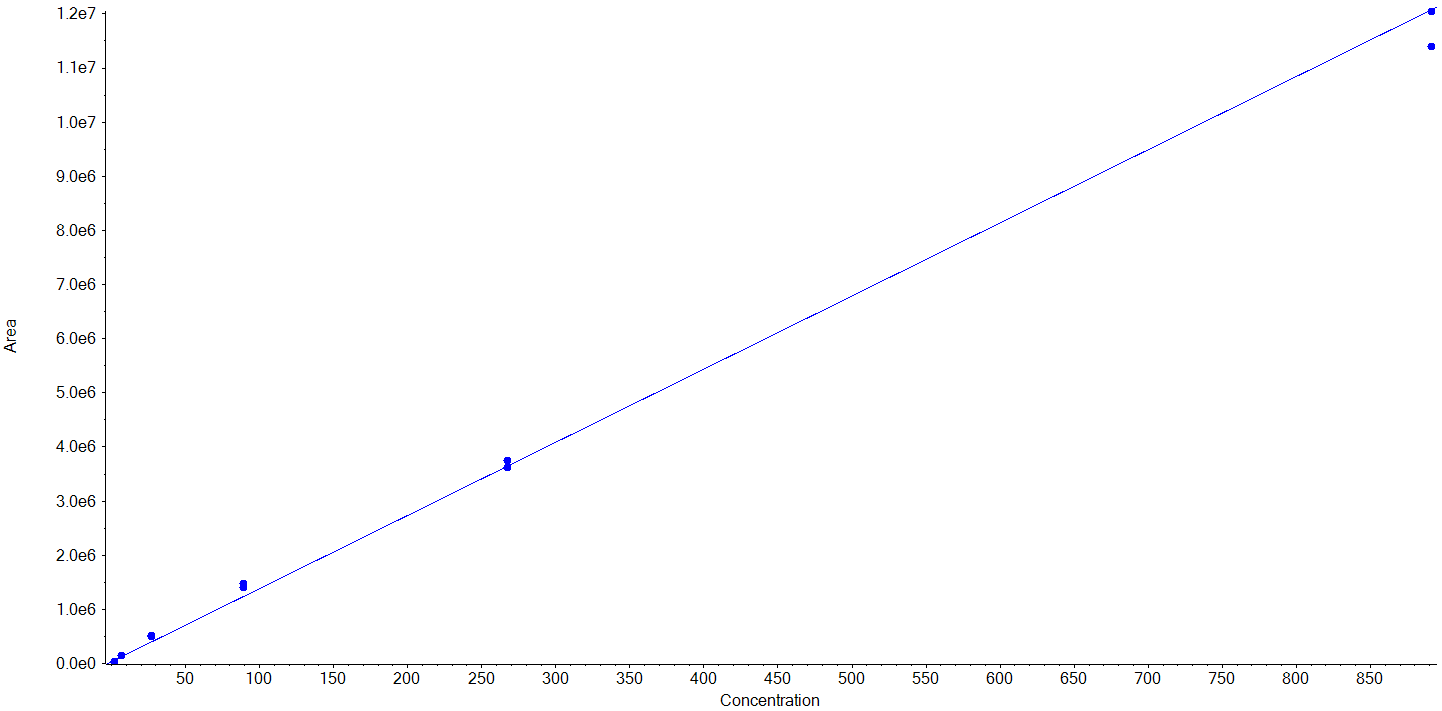 |
| 3,5-Dihydroxybenzoic acid 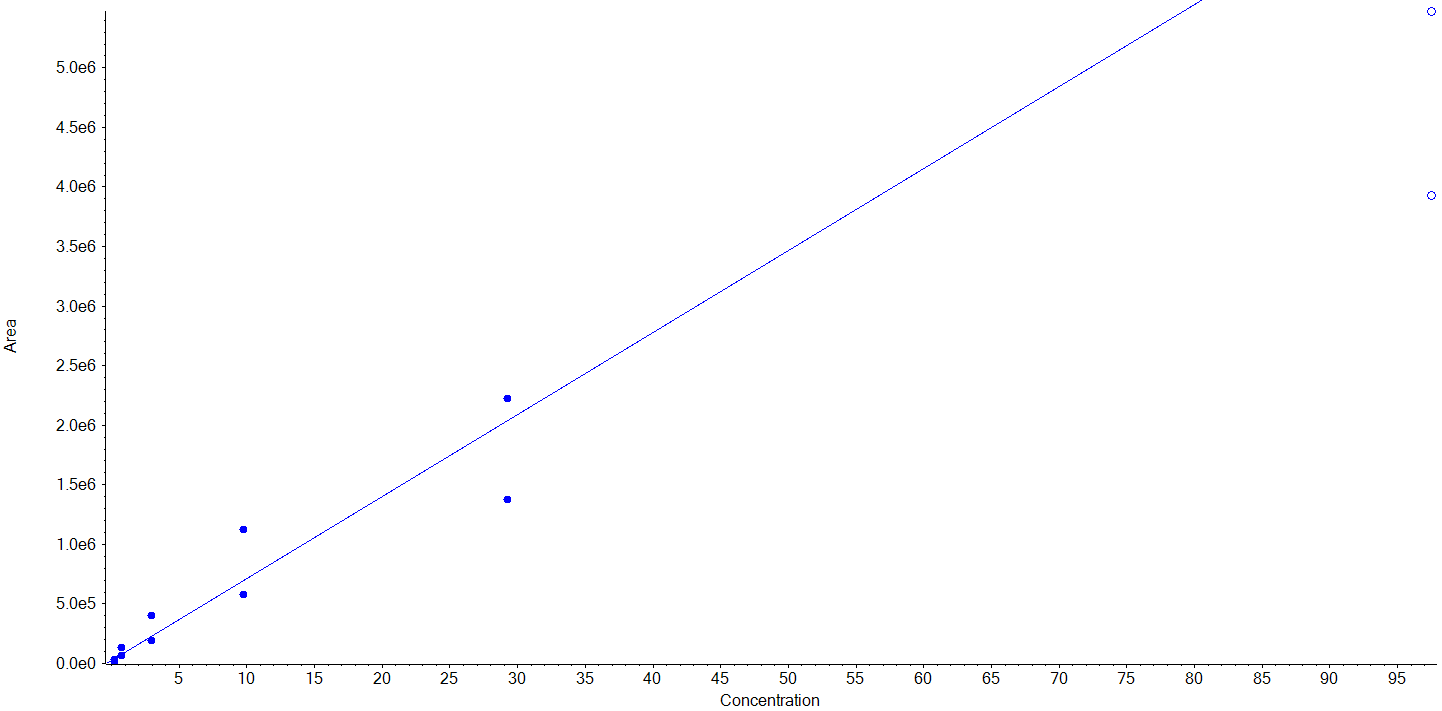 | Hydroxytyrosol 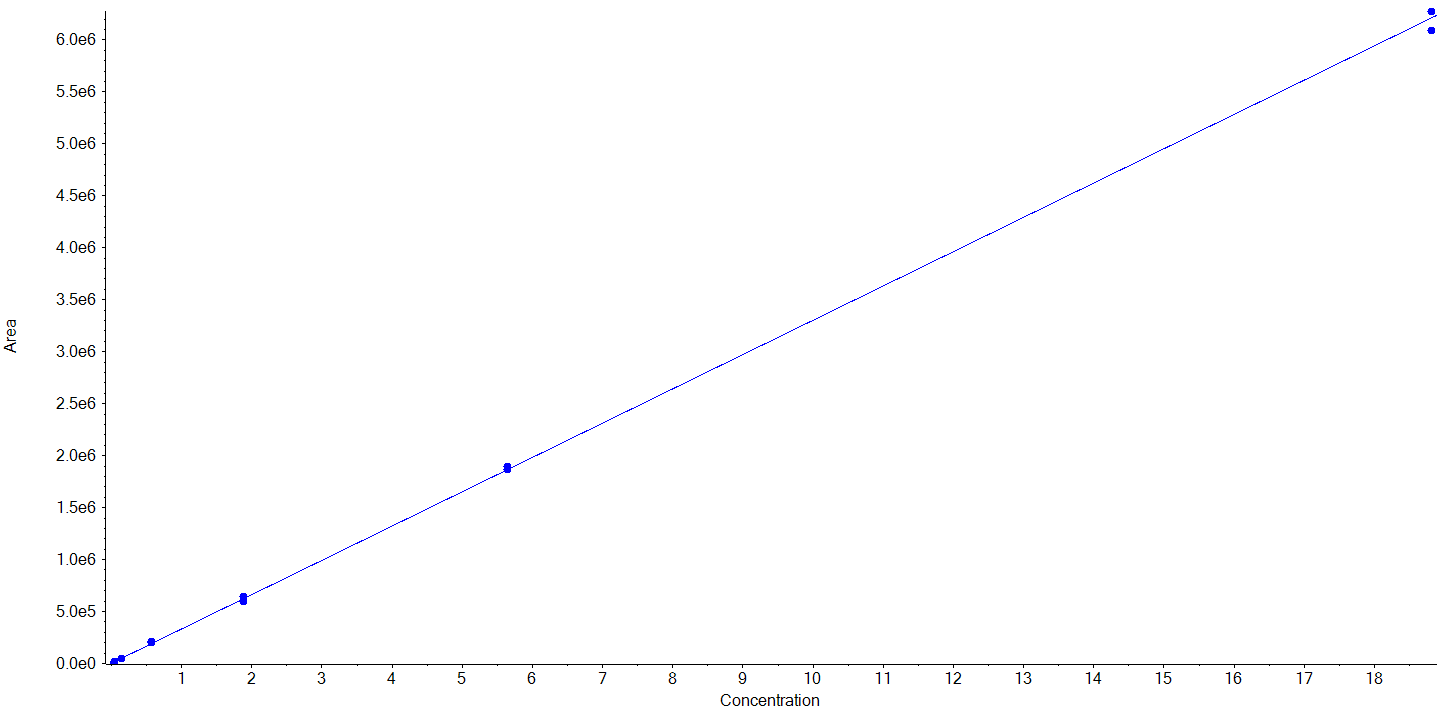 | Protocatechuic acid 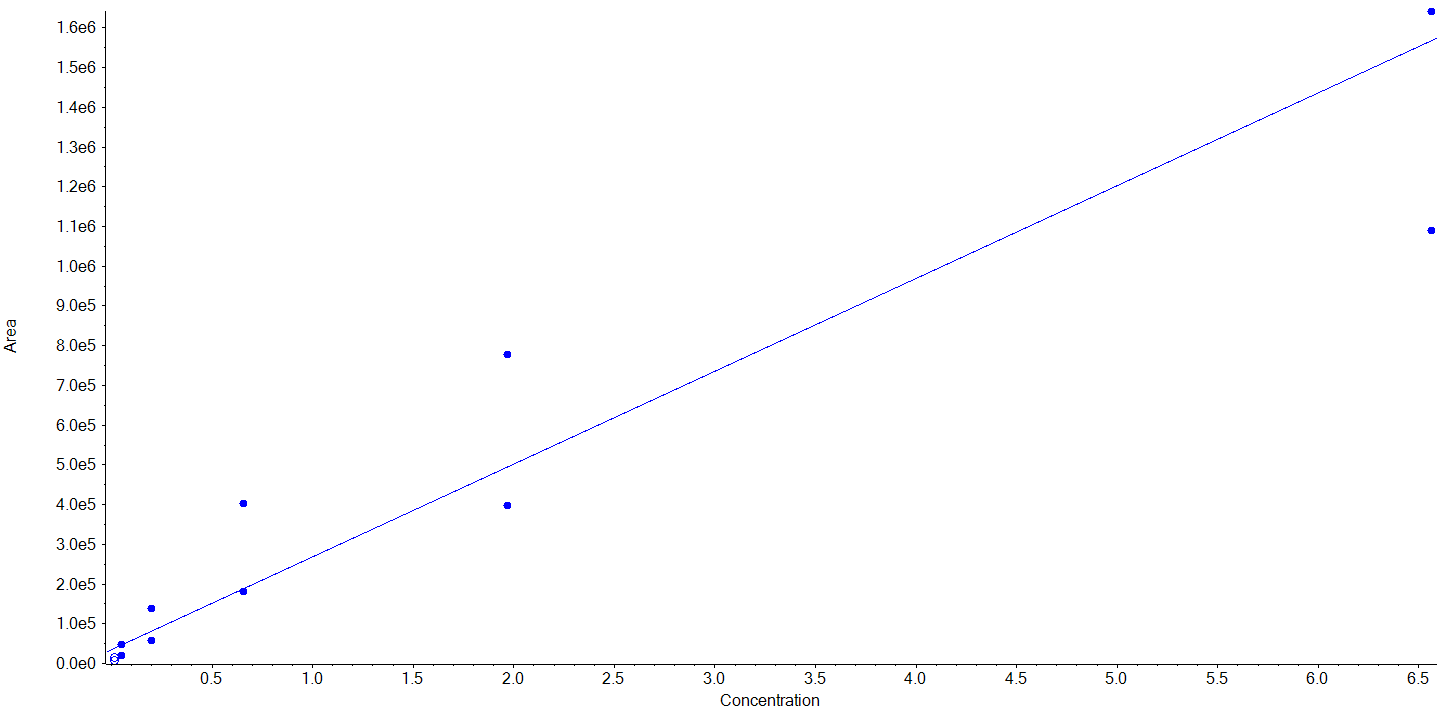 |
| trans-m-Coumaric acid 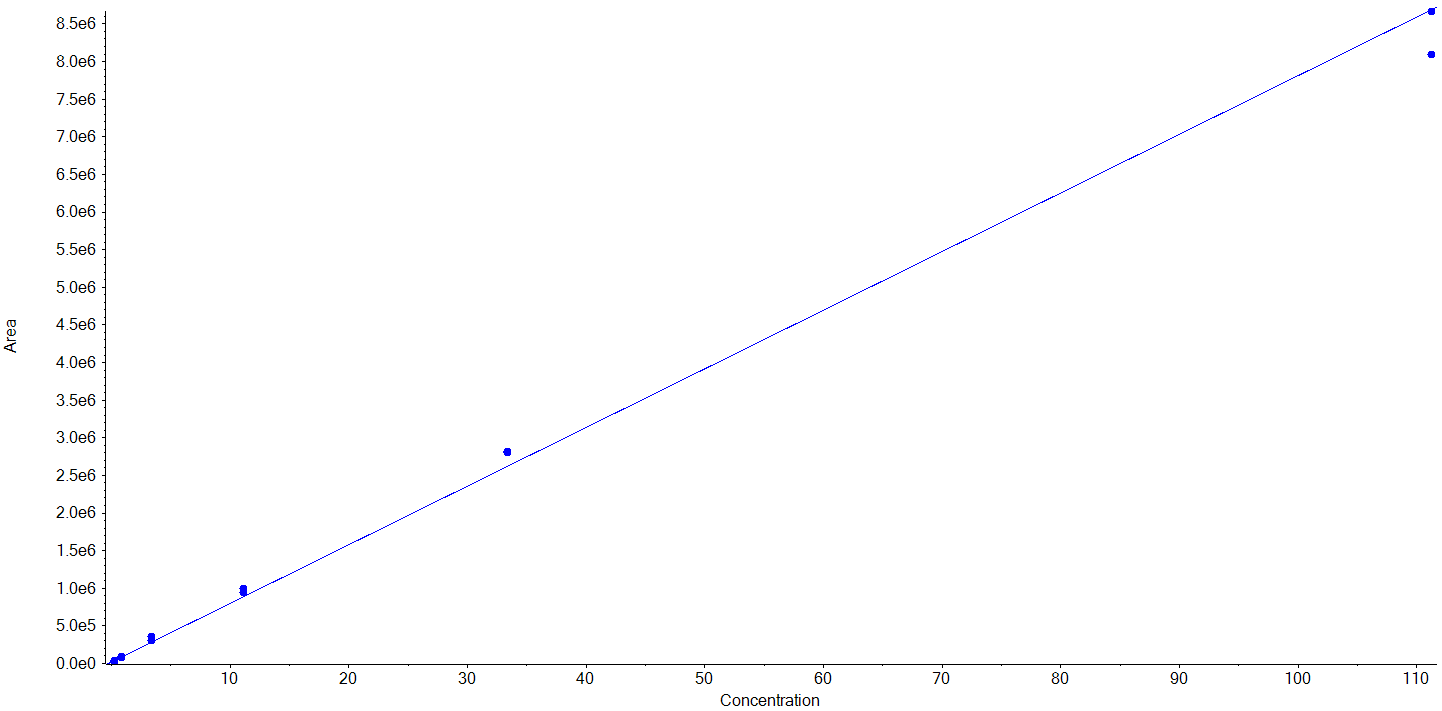 | p-Coumaric acid 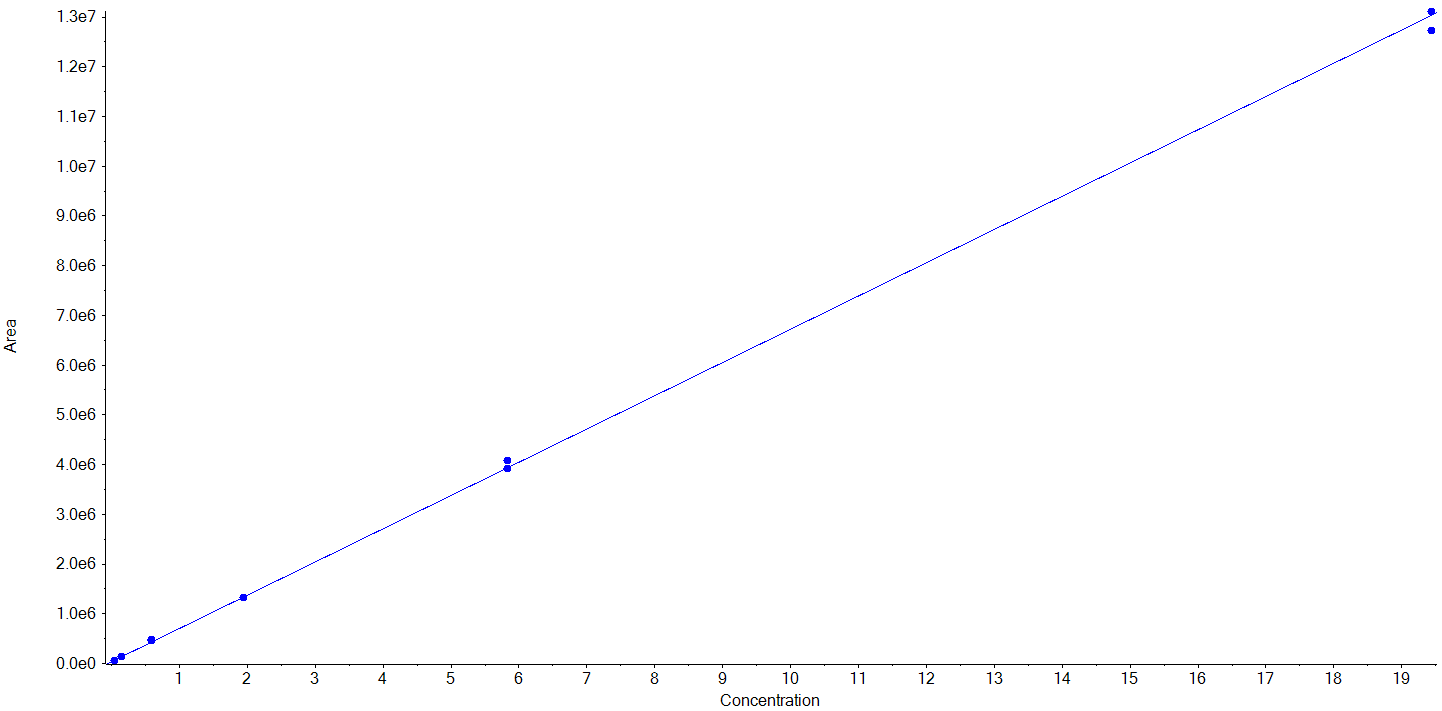 | trans-o-Coumaric acid 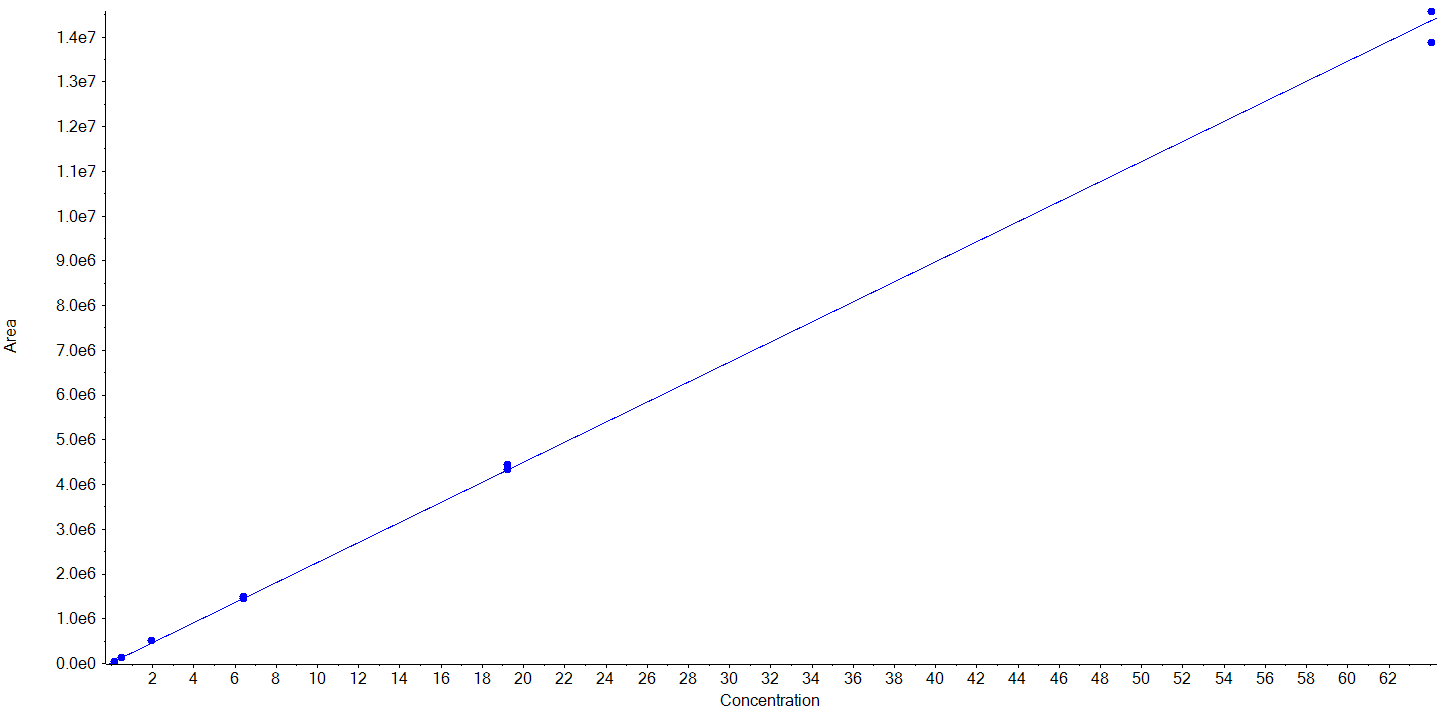 |
| 3-(3-Hydroxyphenyl)propionic acid 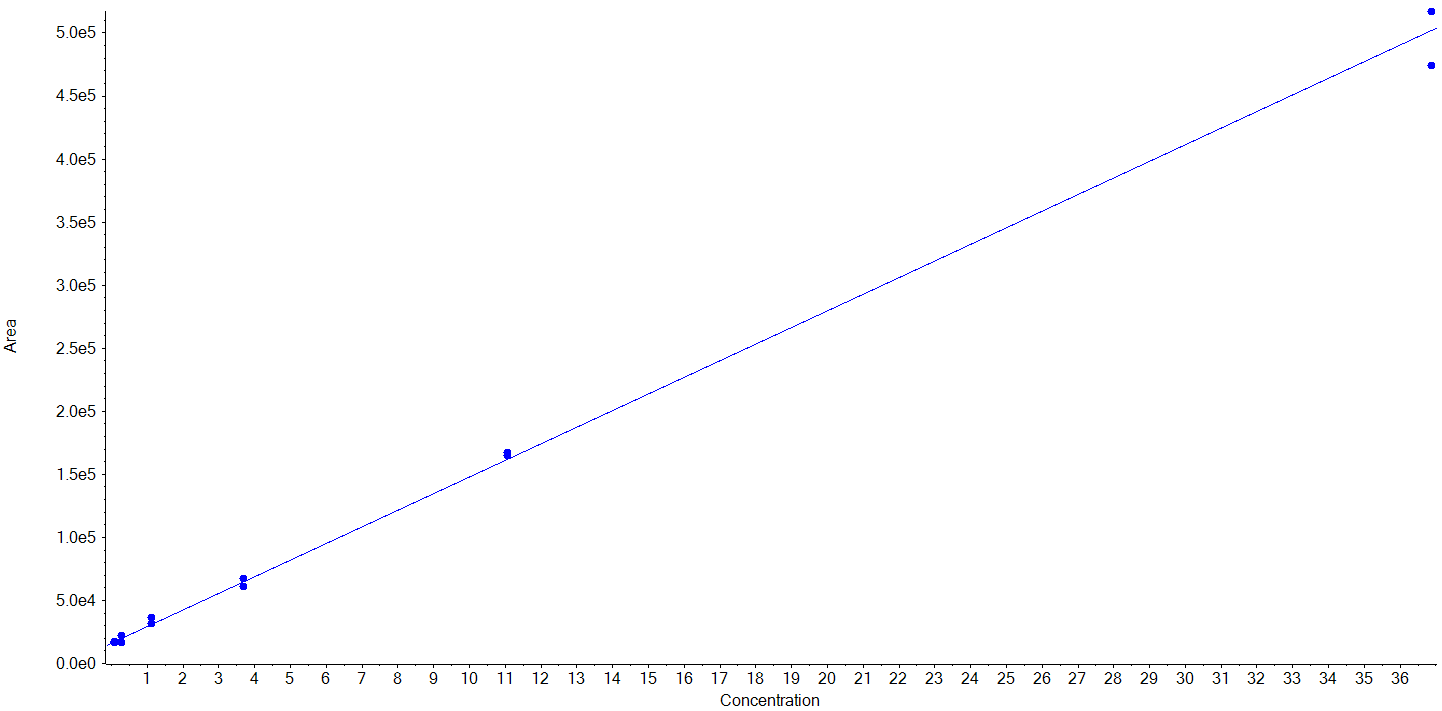 | Vanillic acid 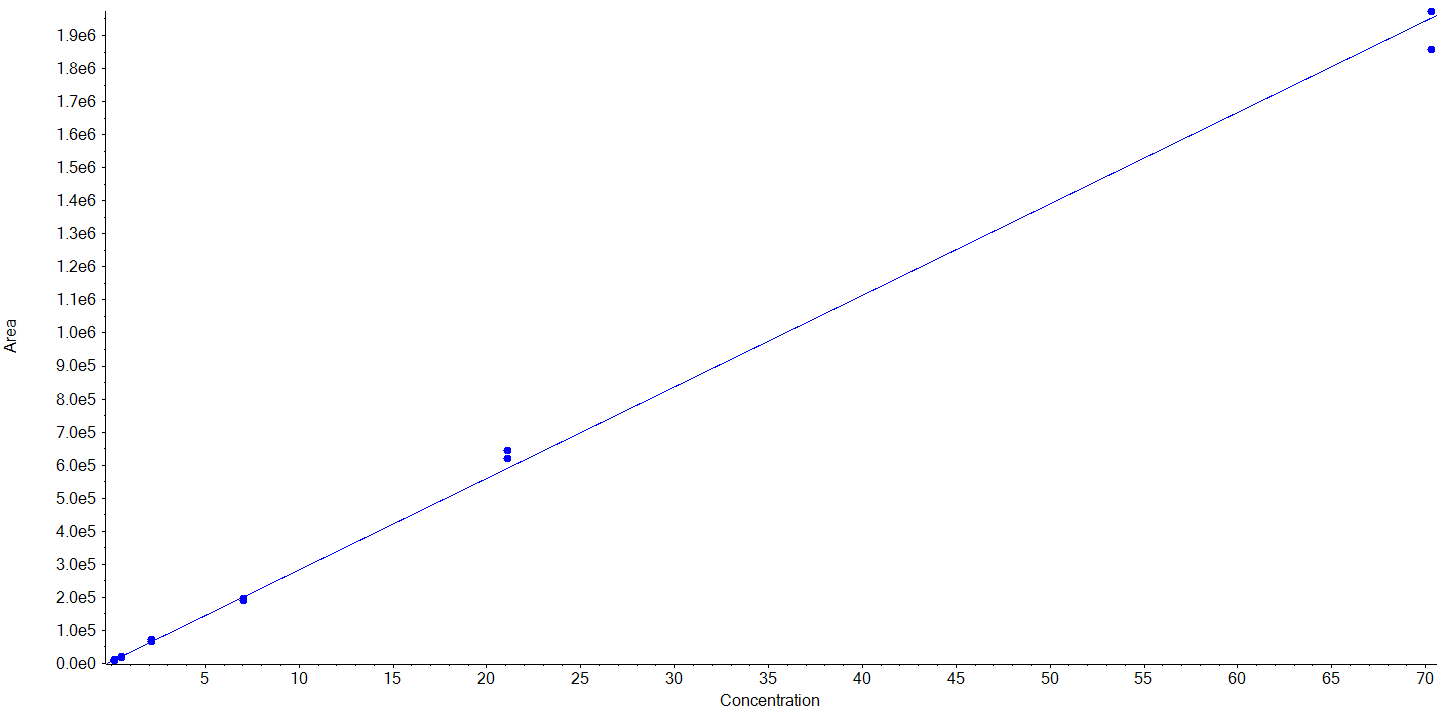 | Homoprotocatechuic acid 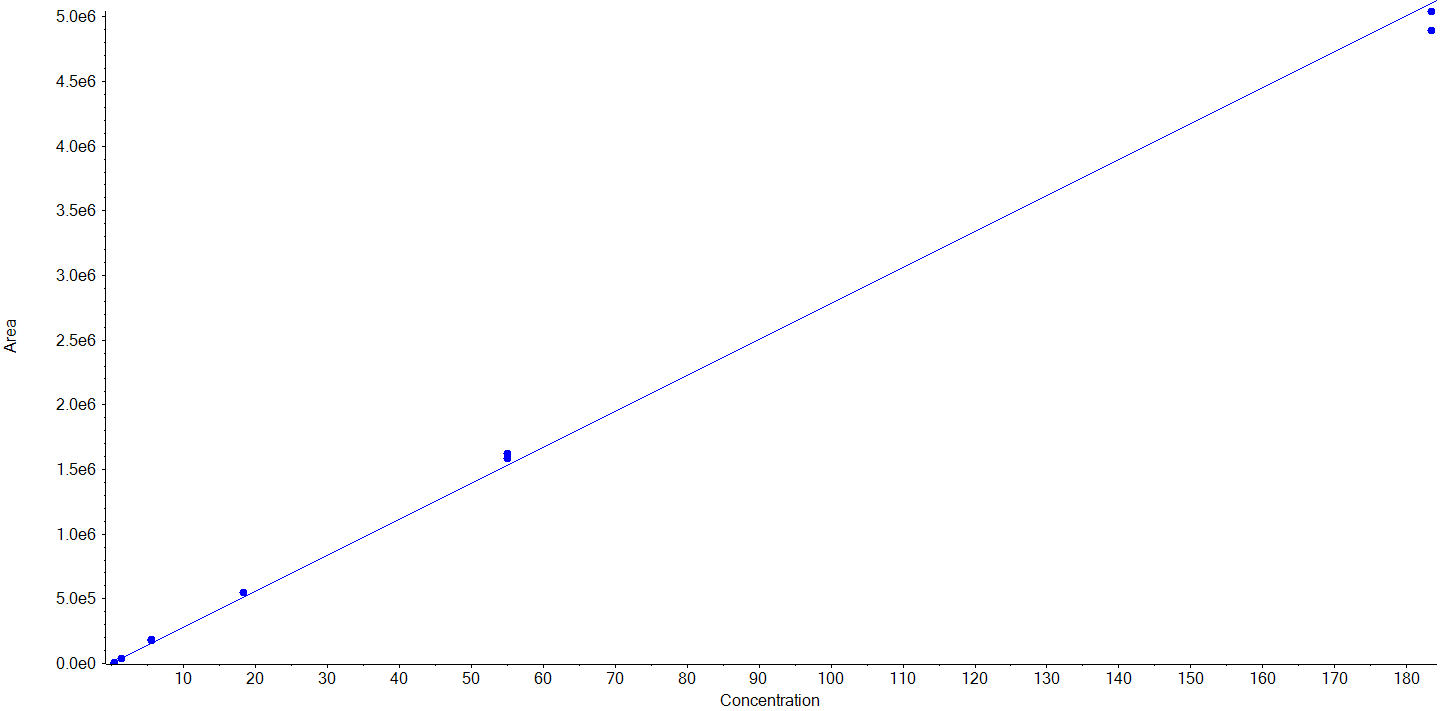 |
| Gallic acid 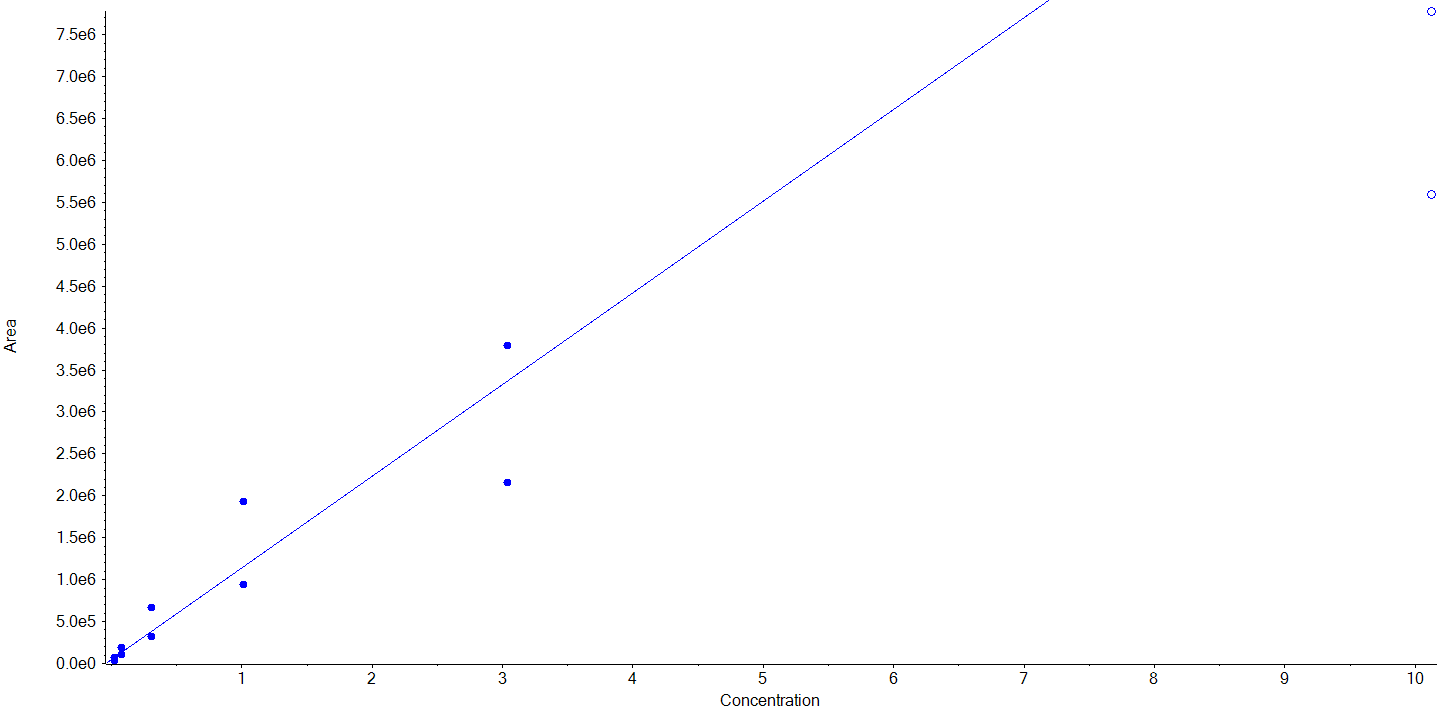 | Caffeic acid 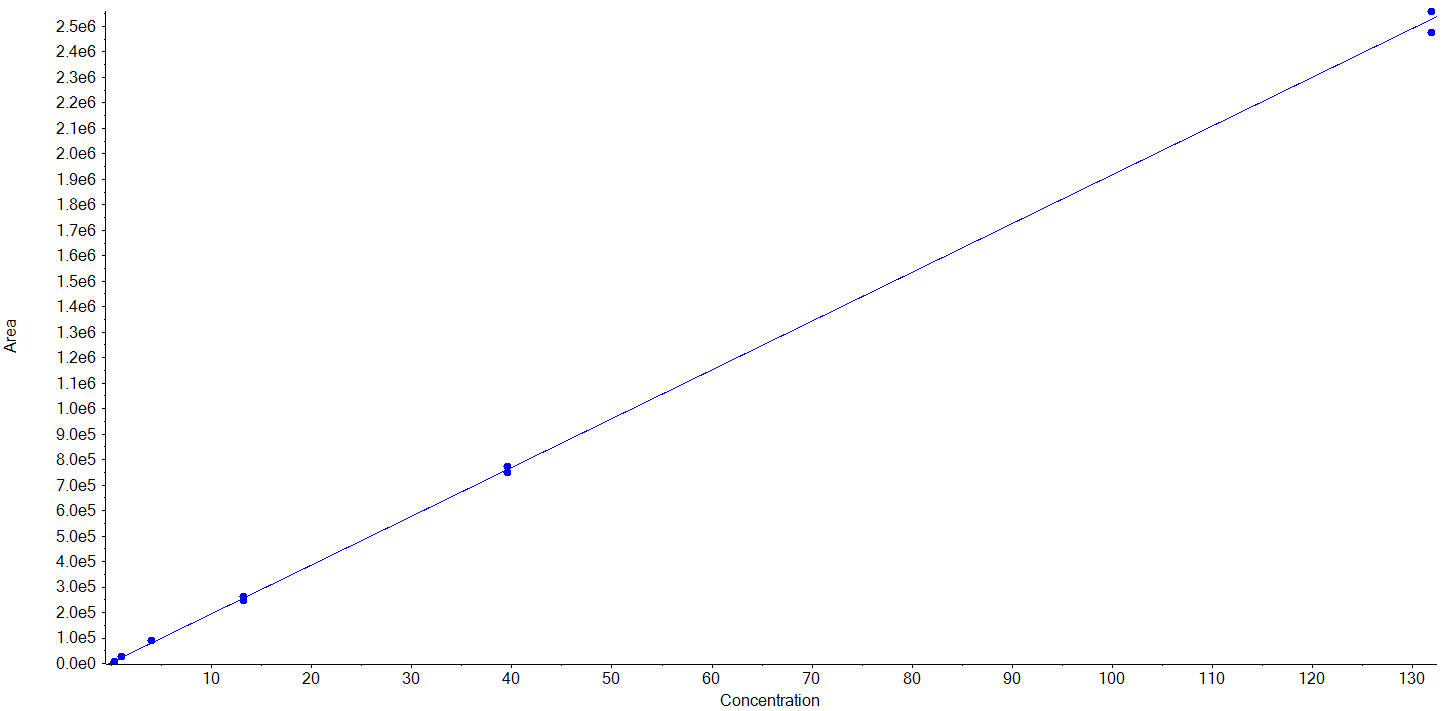 | Homovanillic acid 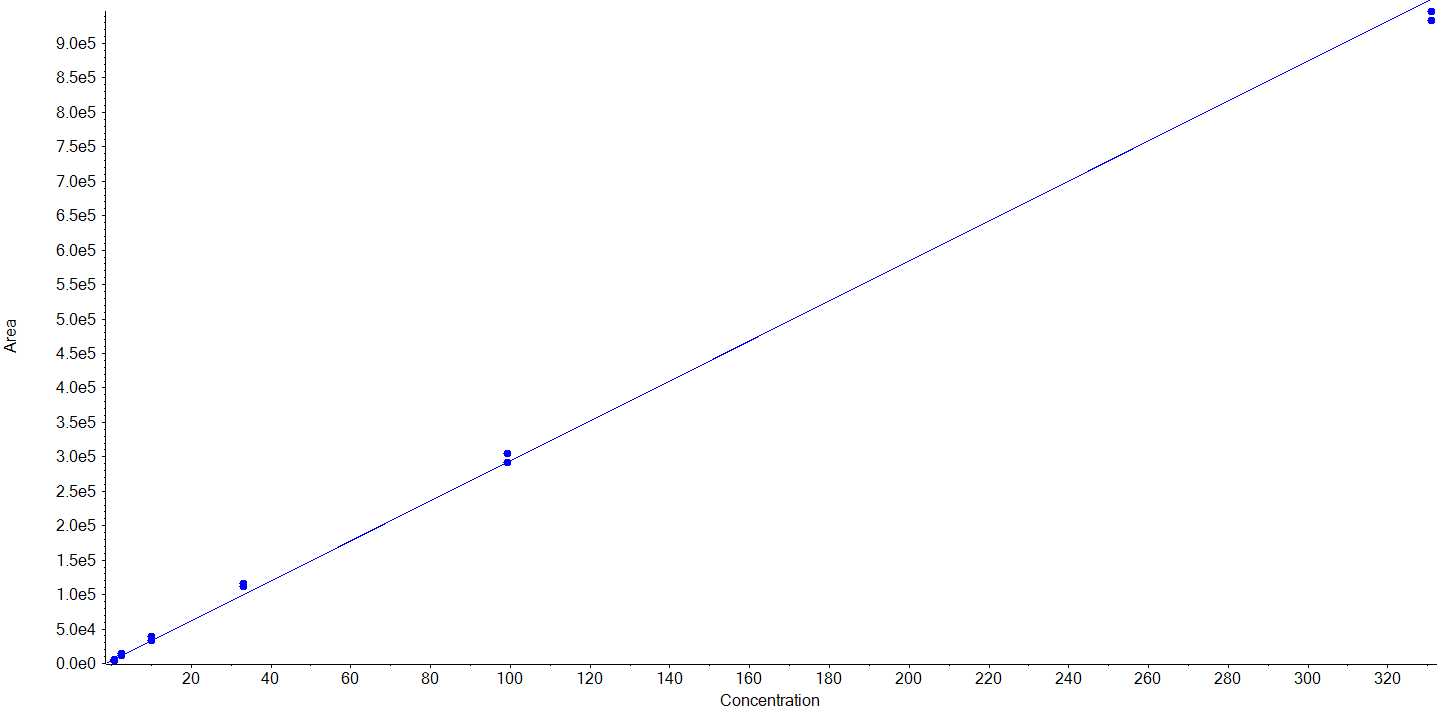 |
| Dihydrocaffeic acid 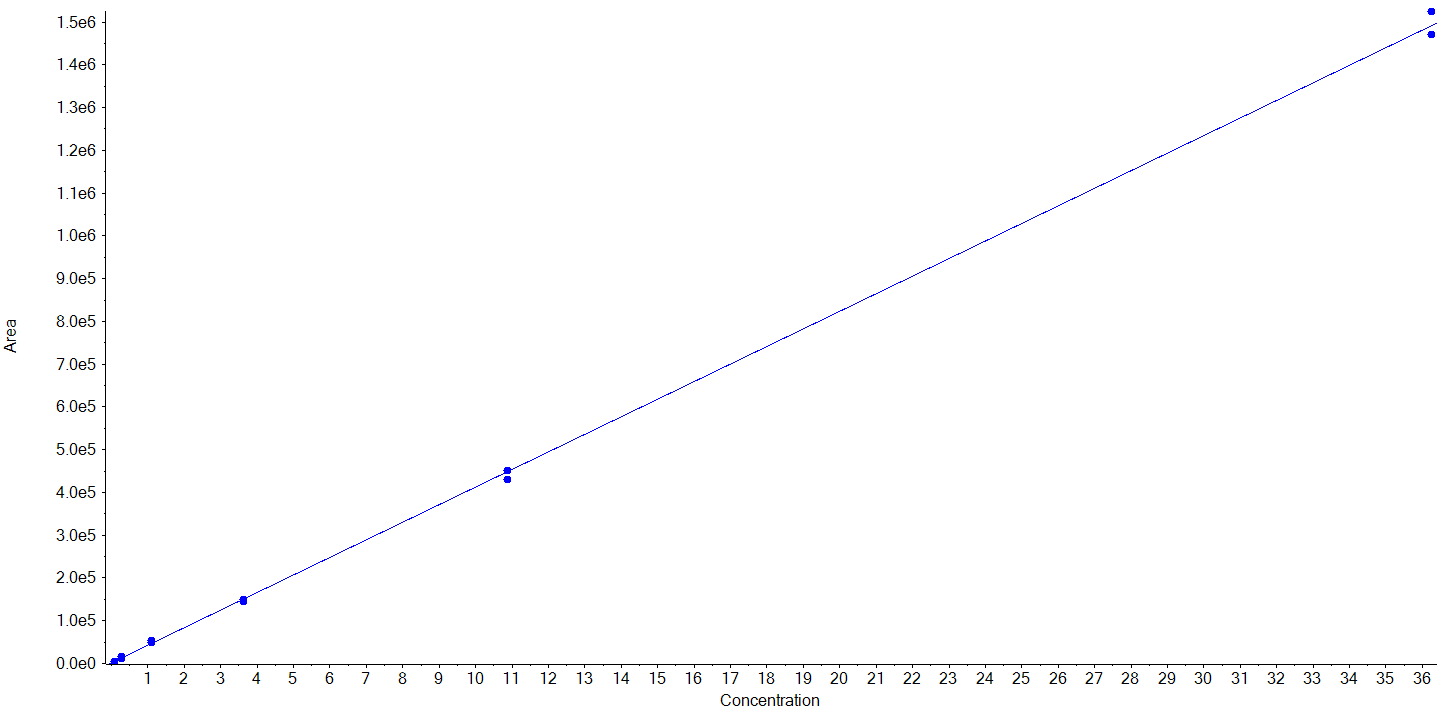 | Ferulic acid/Isoferulic acid 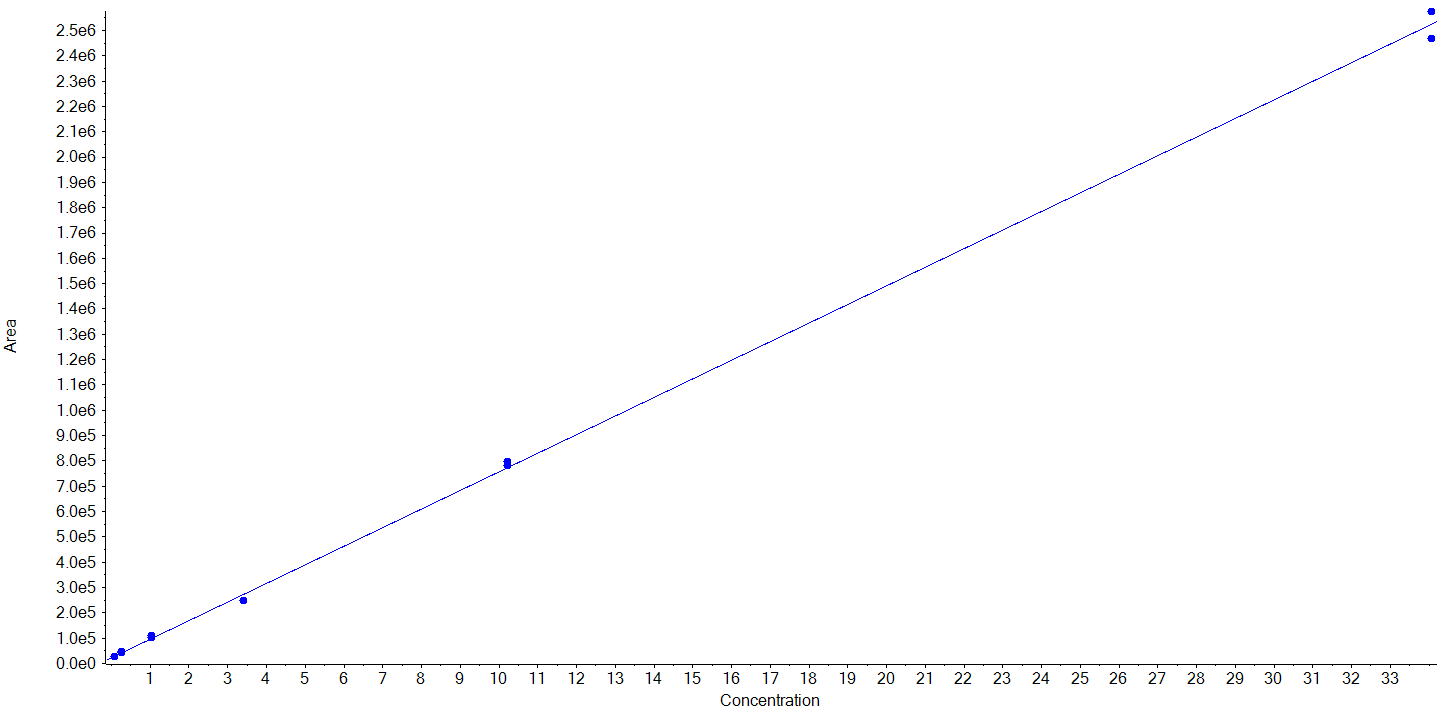 | Dihydroferulic acid 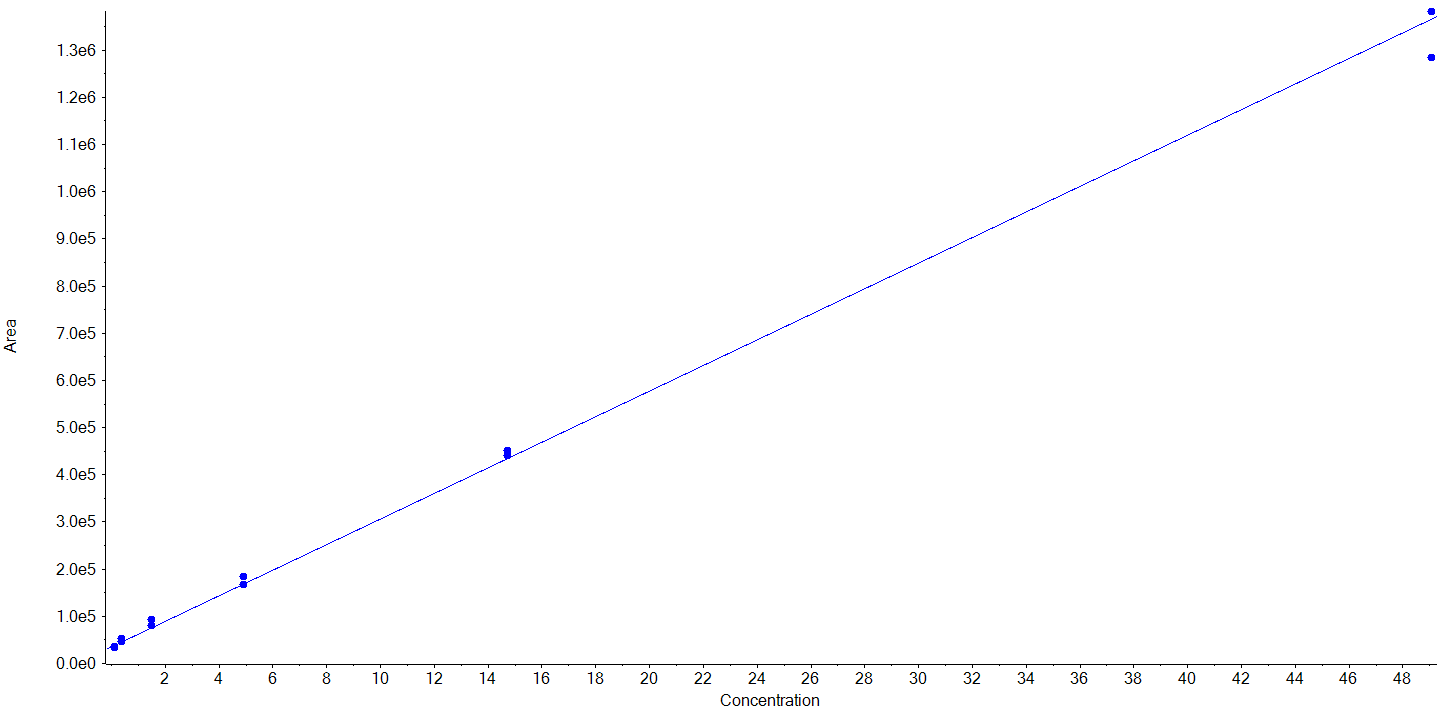 |
| Syringic acid 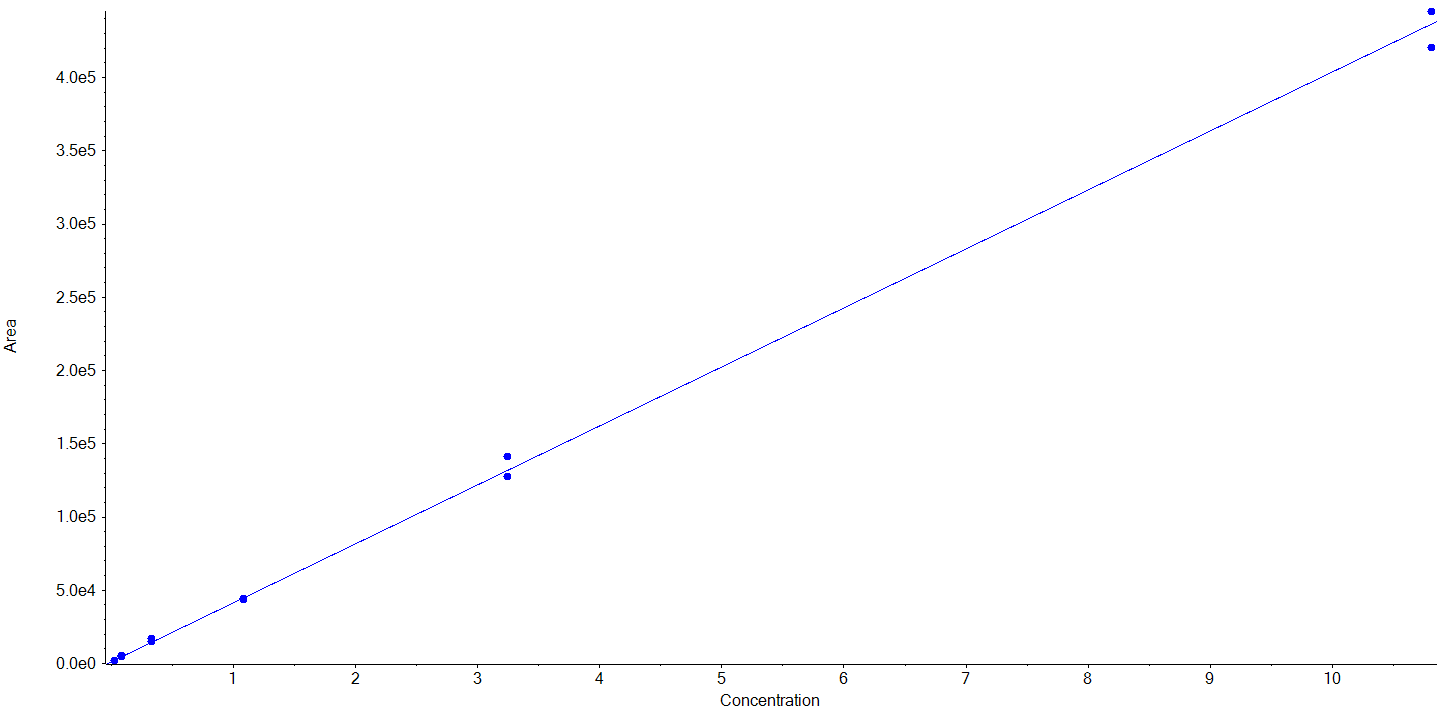 | Ethyl gallate 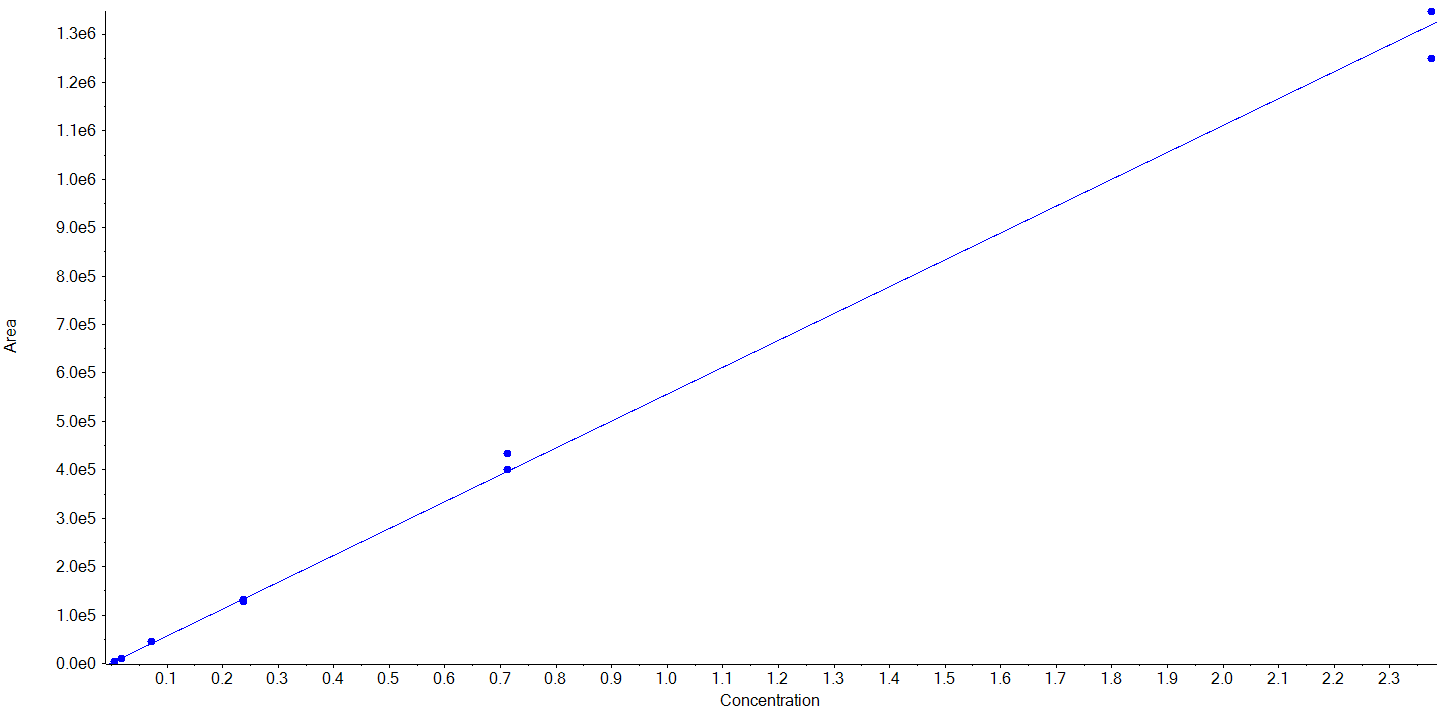 | 3,5-Dimethoxy-4 -hydroxyphenylacetic acid 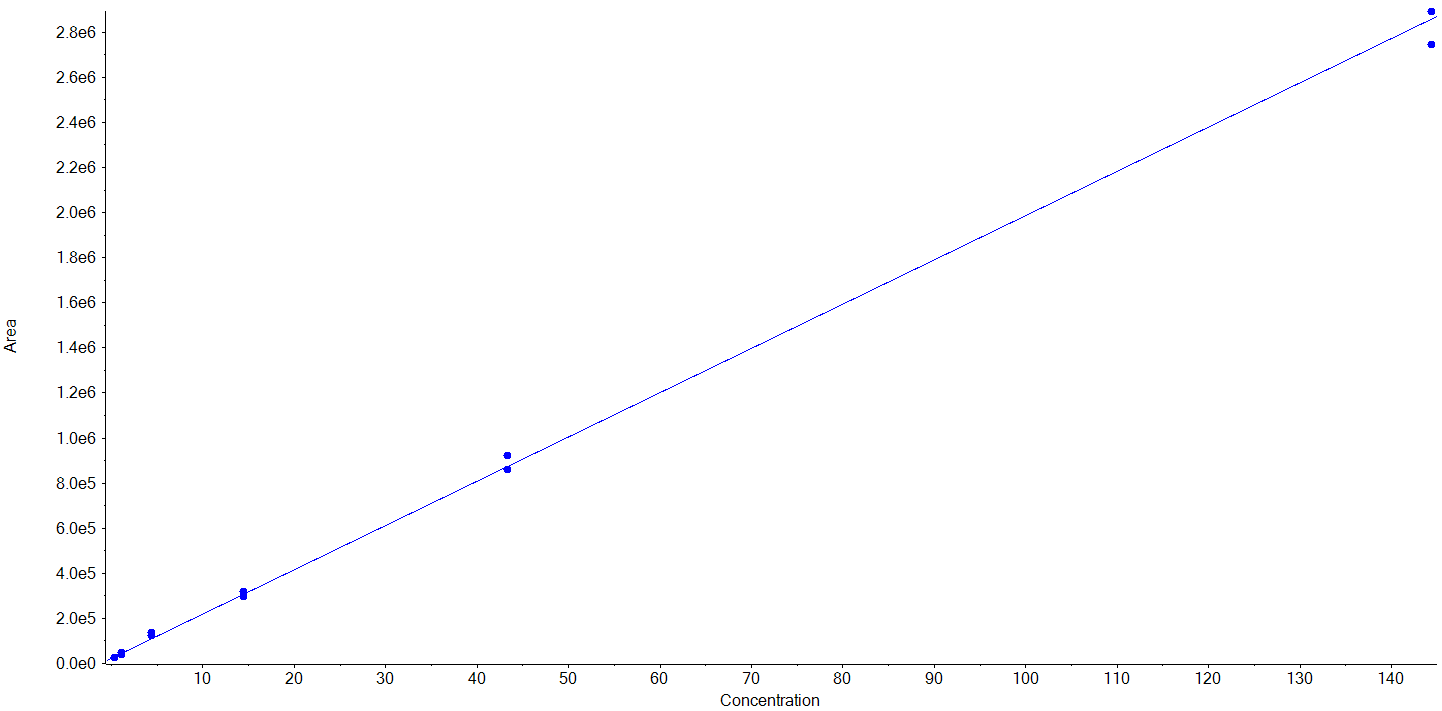 |
| Sinapic acid 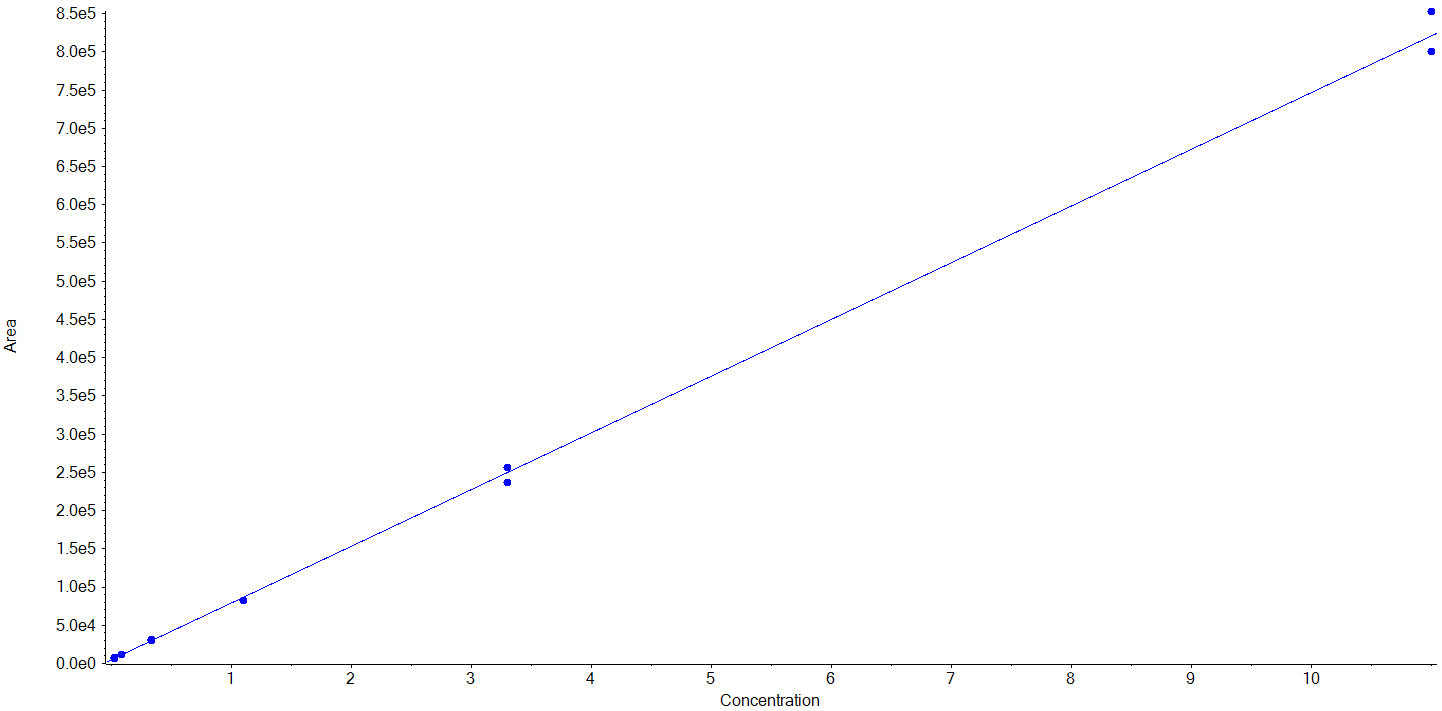 | Urolithin A 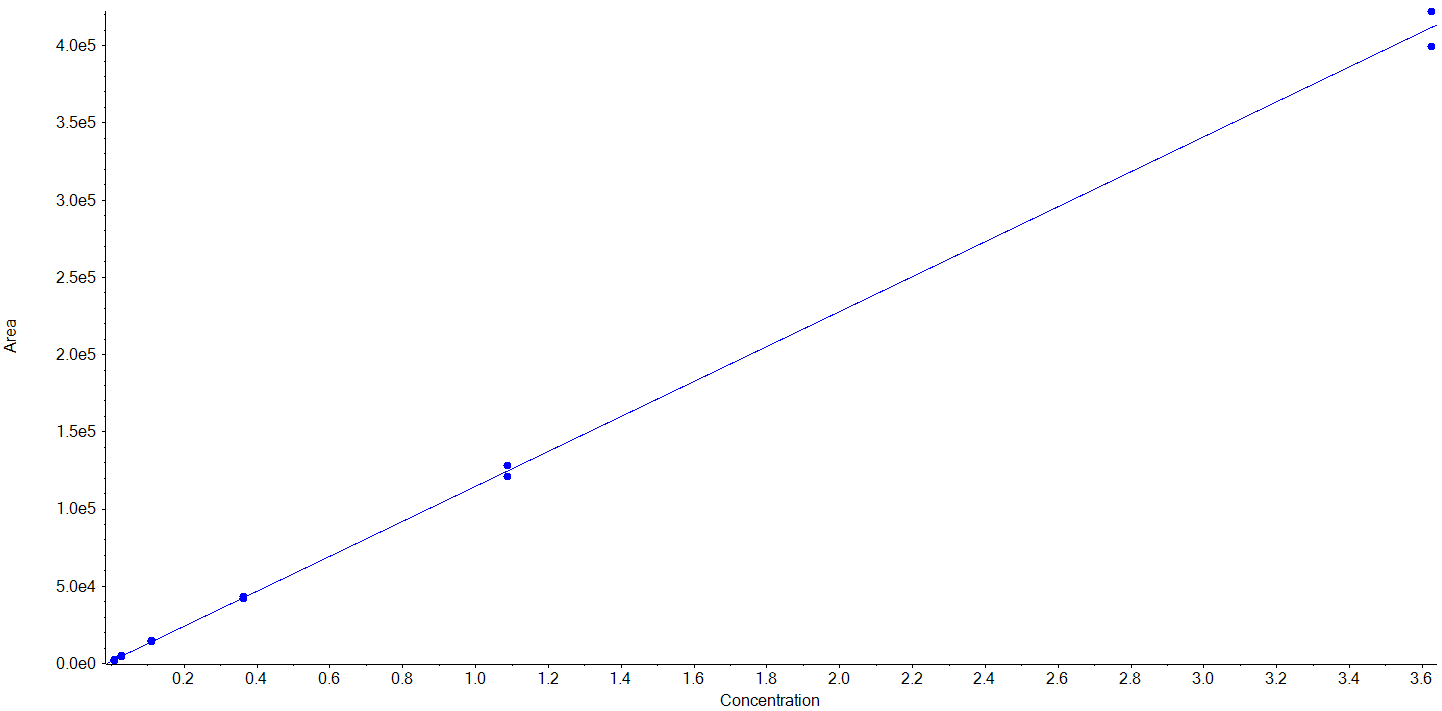 | Resveratrol 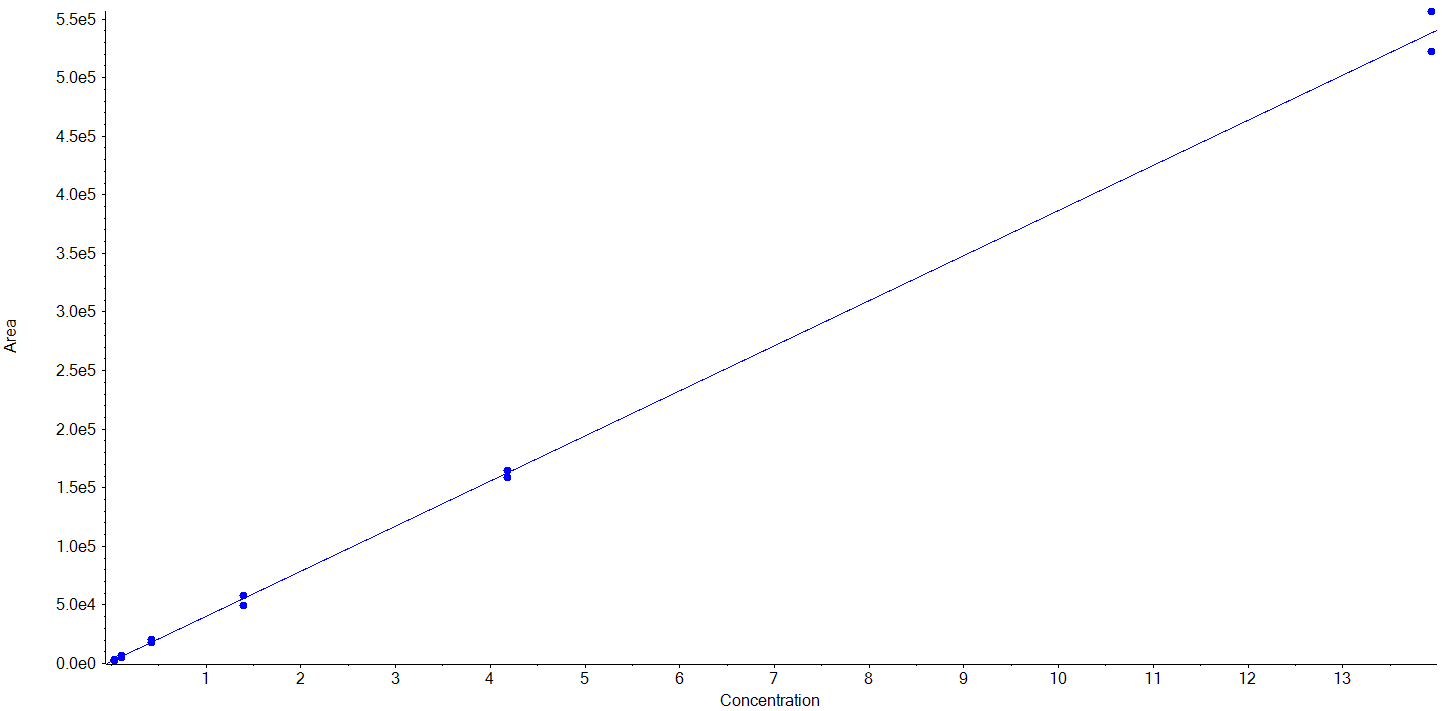 |
| S-Equol 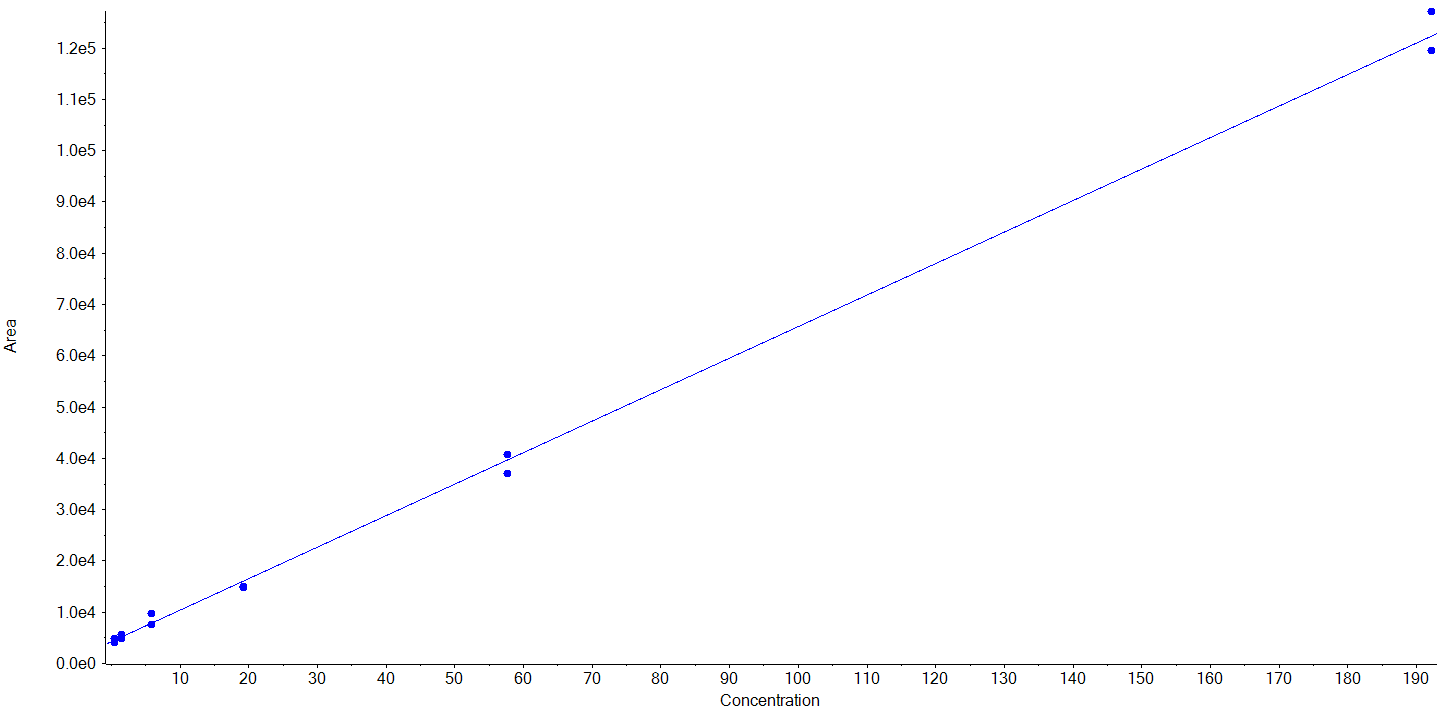 | Daidzein 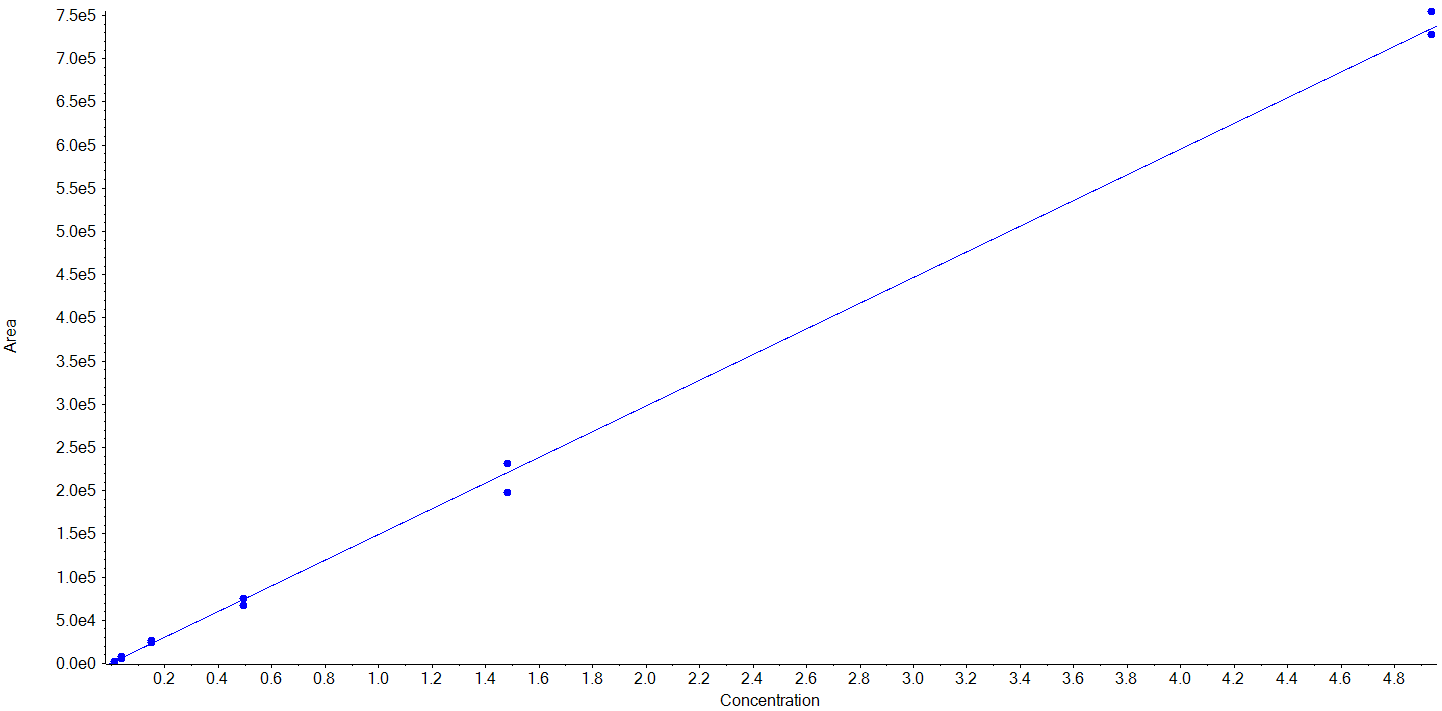 | Apigenin 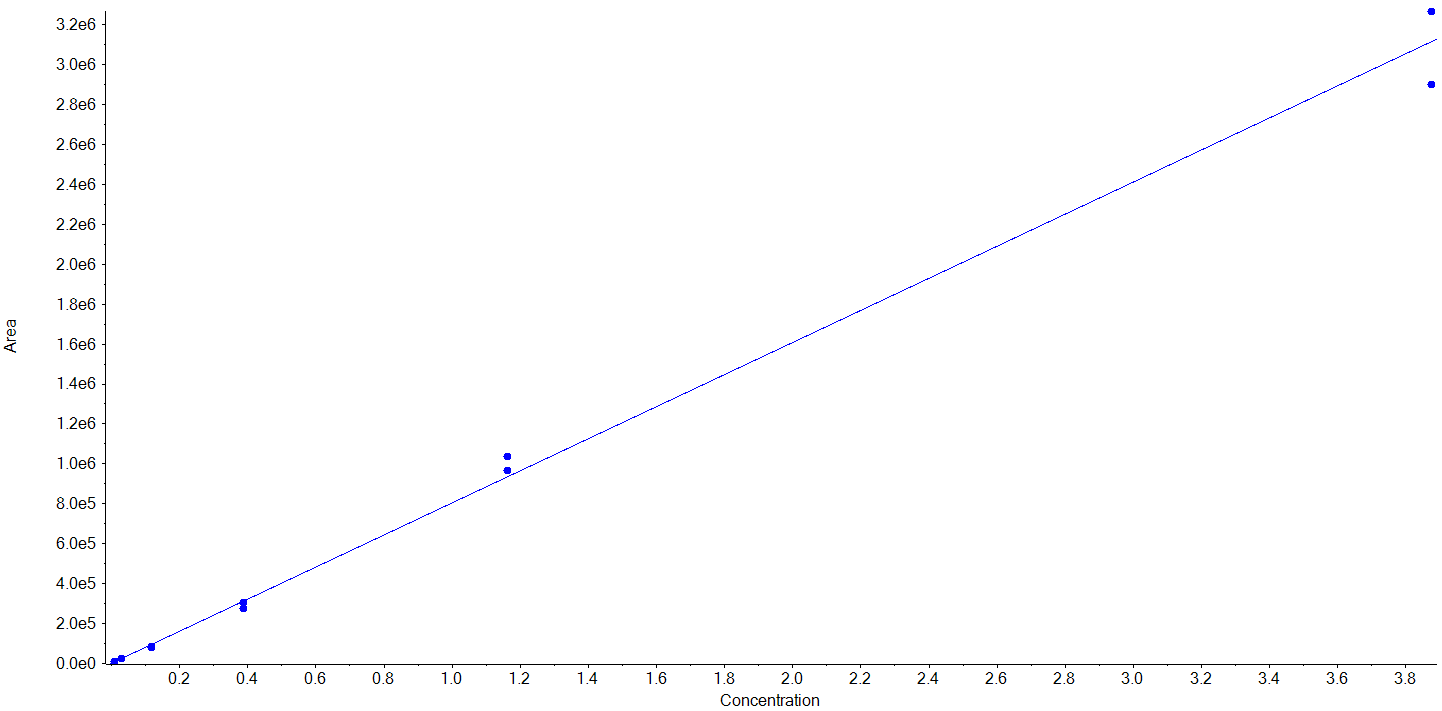 |
| Genistein 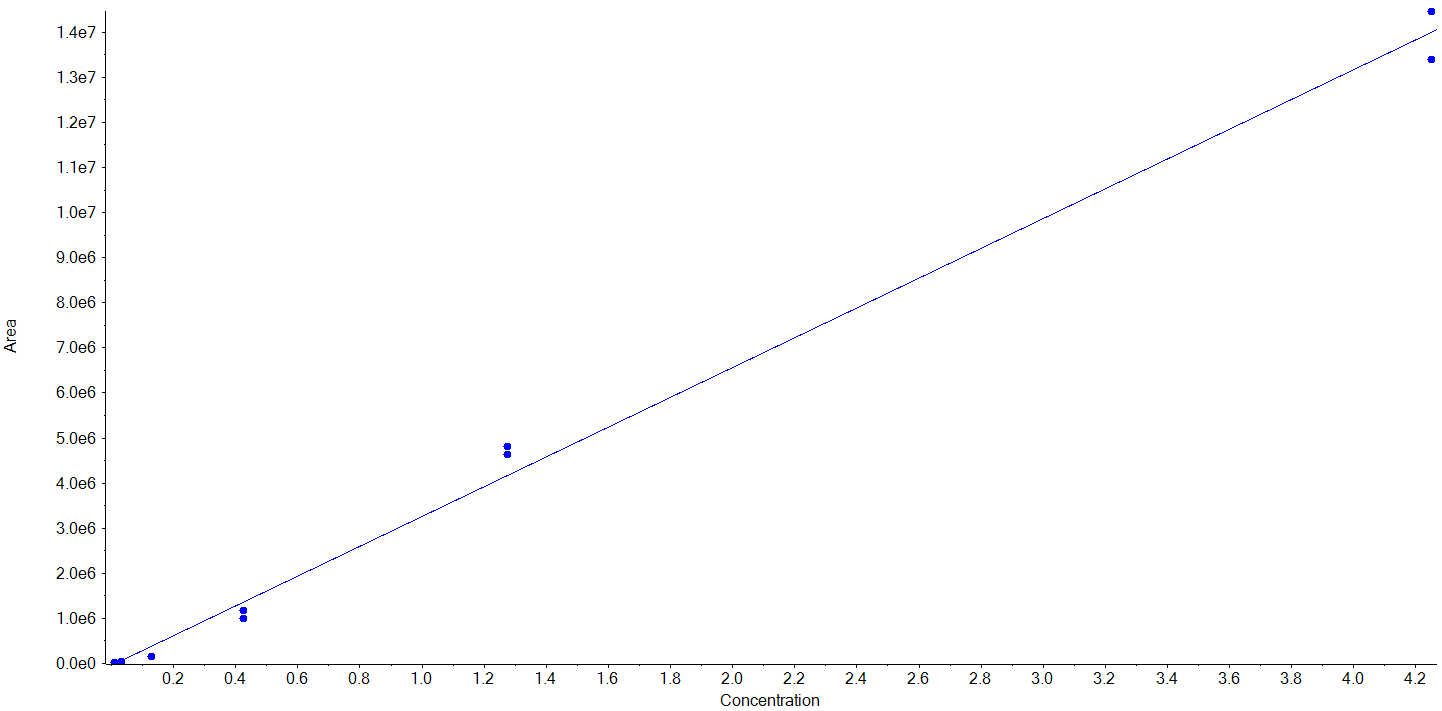 | (+/-)-Naringenin 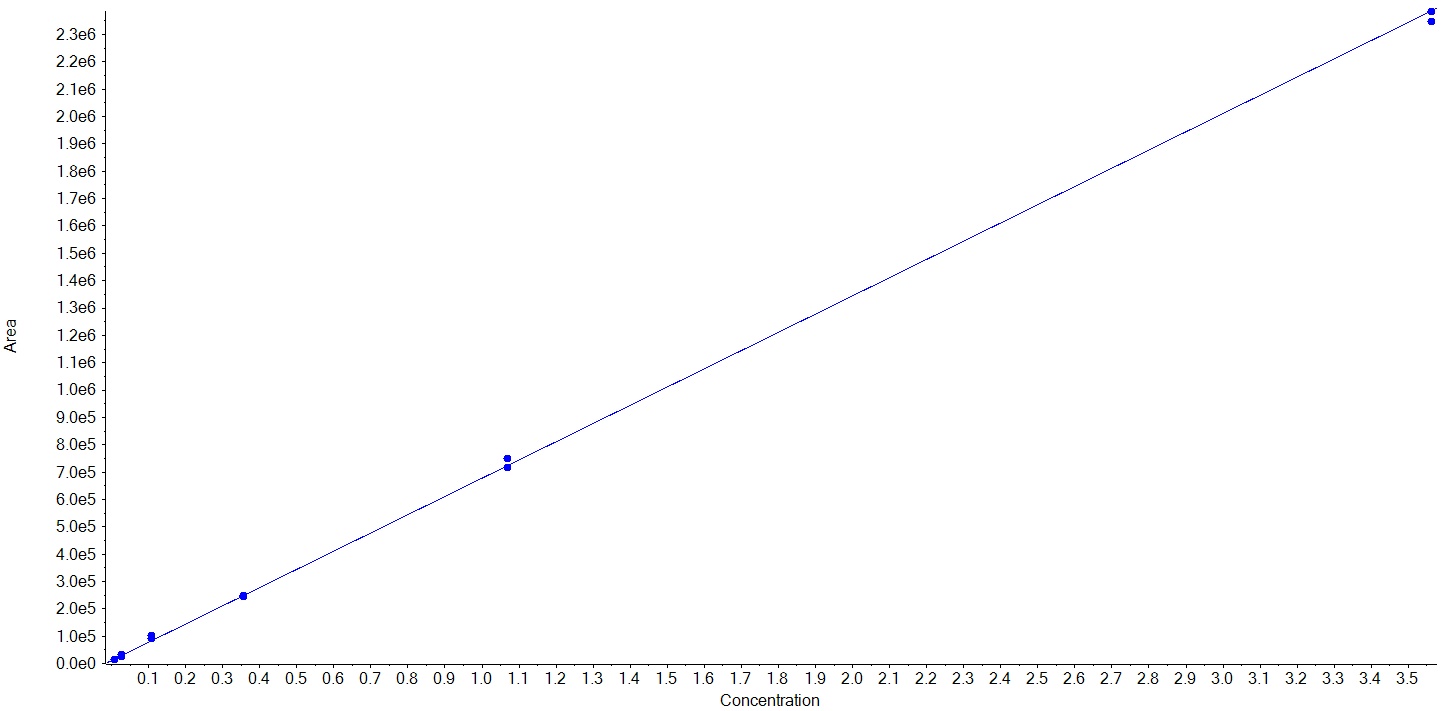 | Phloretin 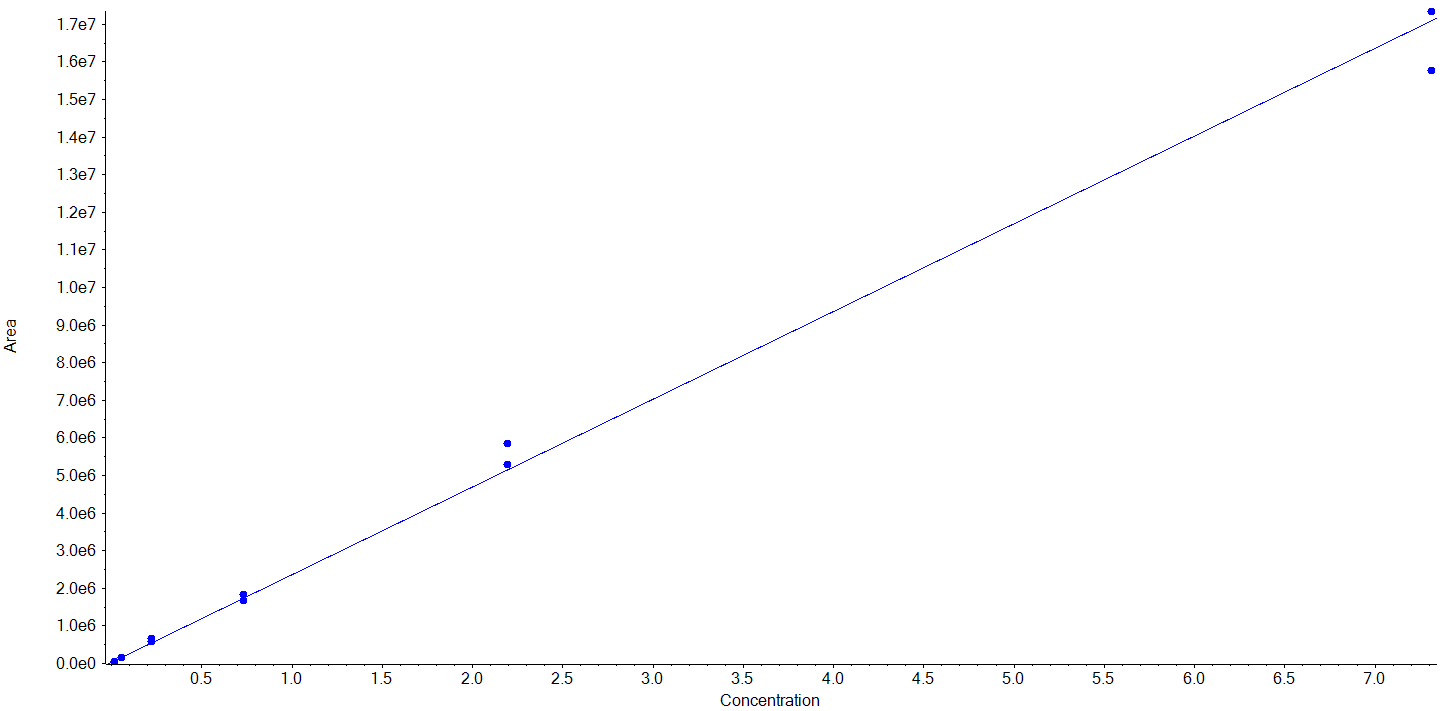 |
| Kaempferol 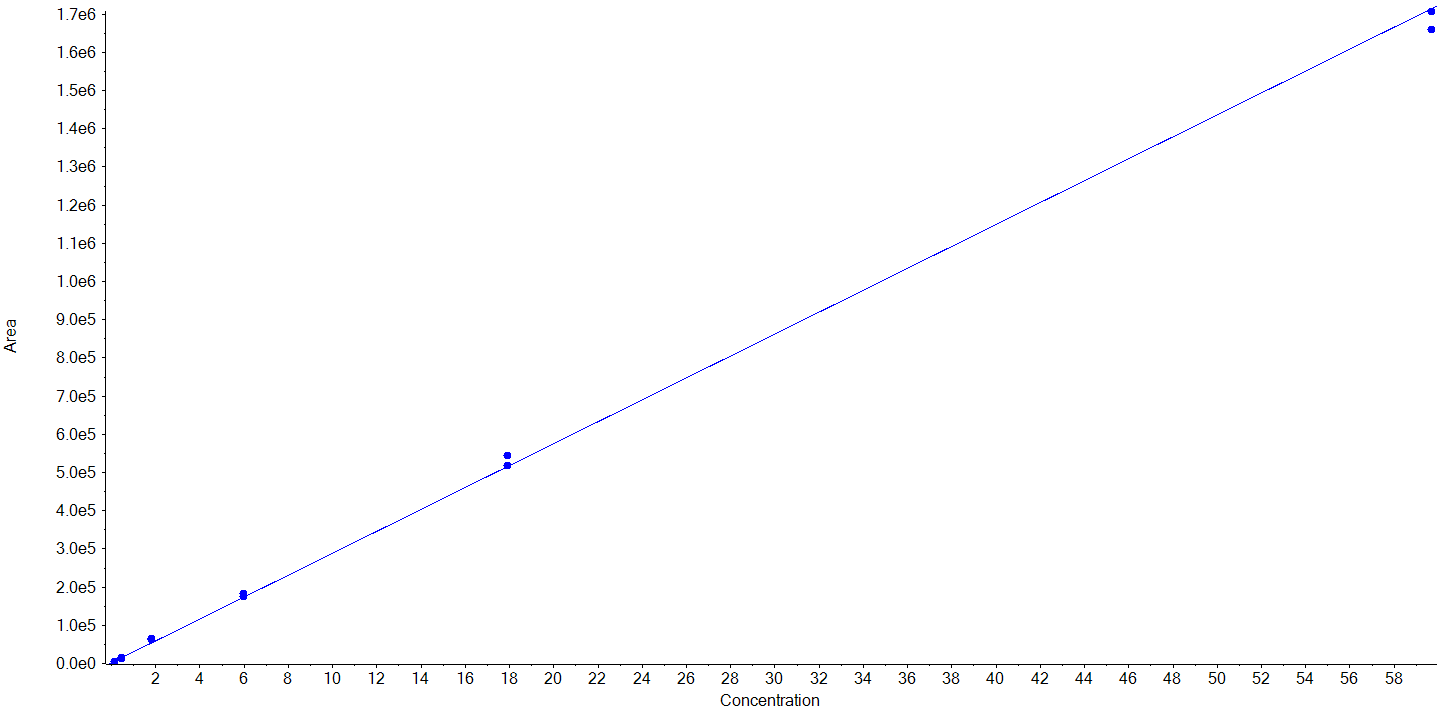 | (+)-Catechin 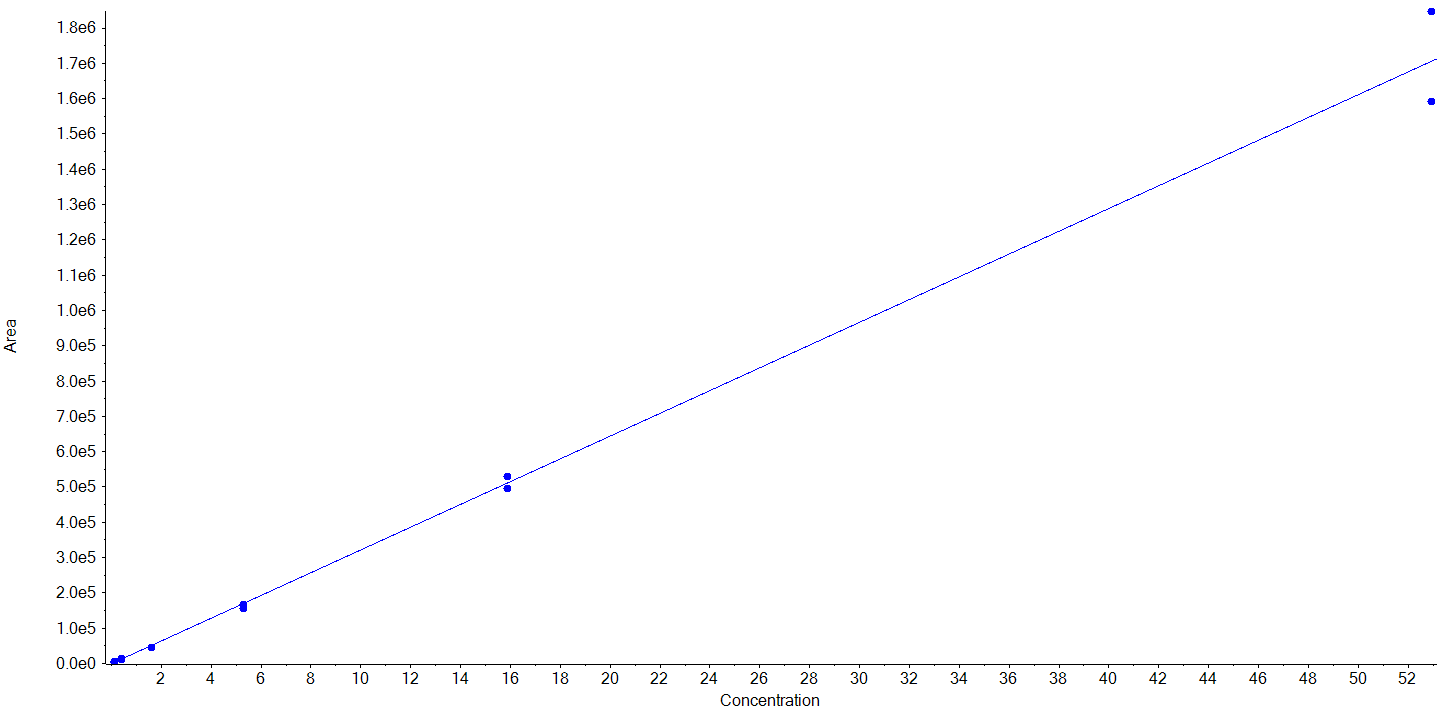 | (-)- Epicatechin 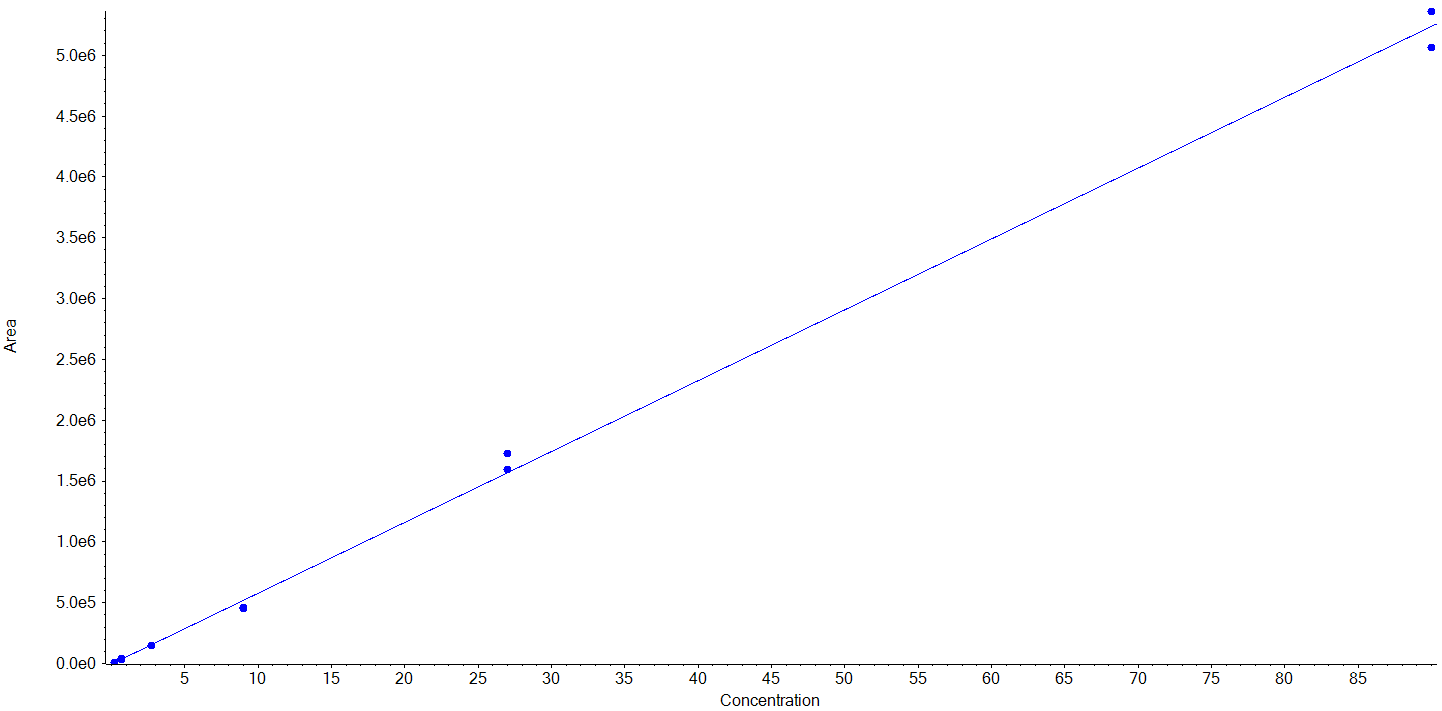 |
| Enterolactone 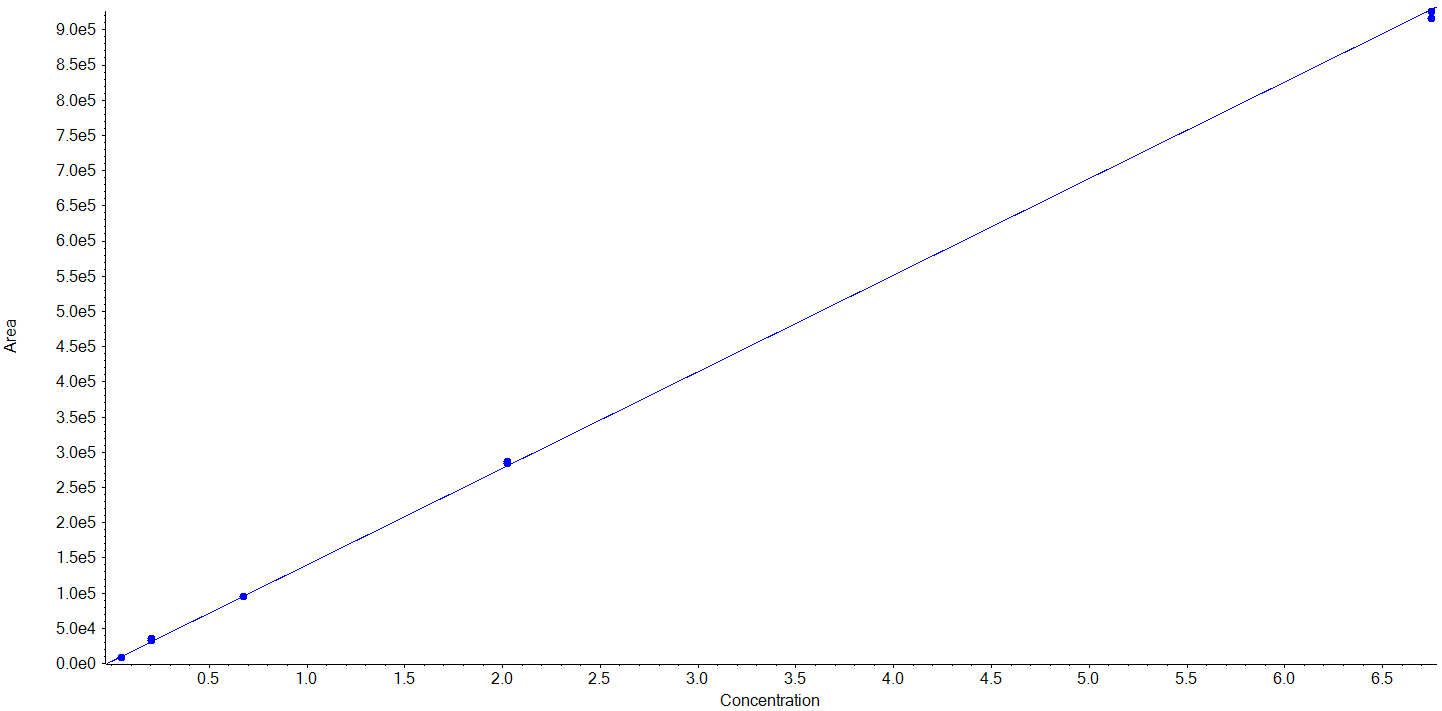 | Diosmetin 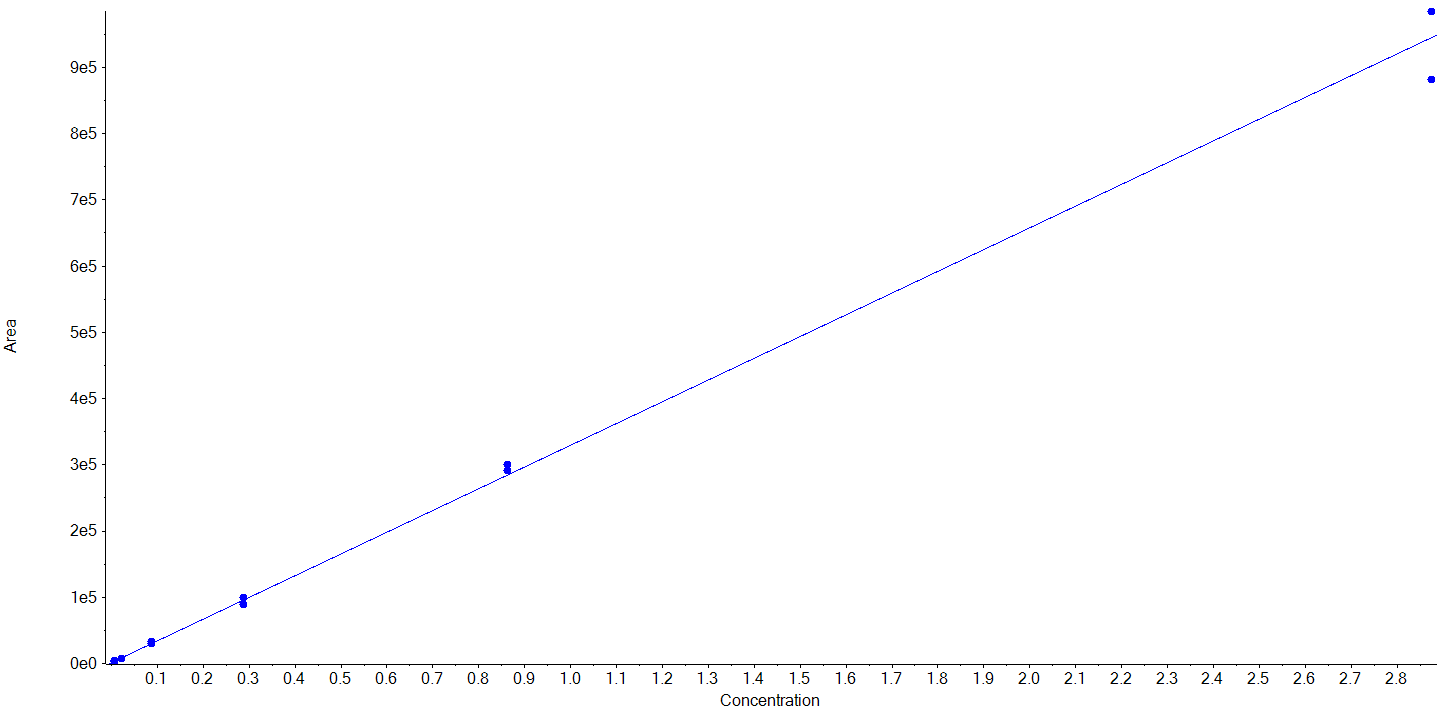 | Ellagic acid 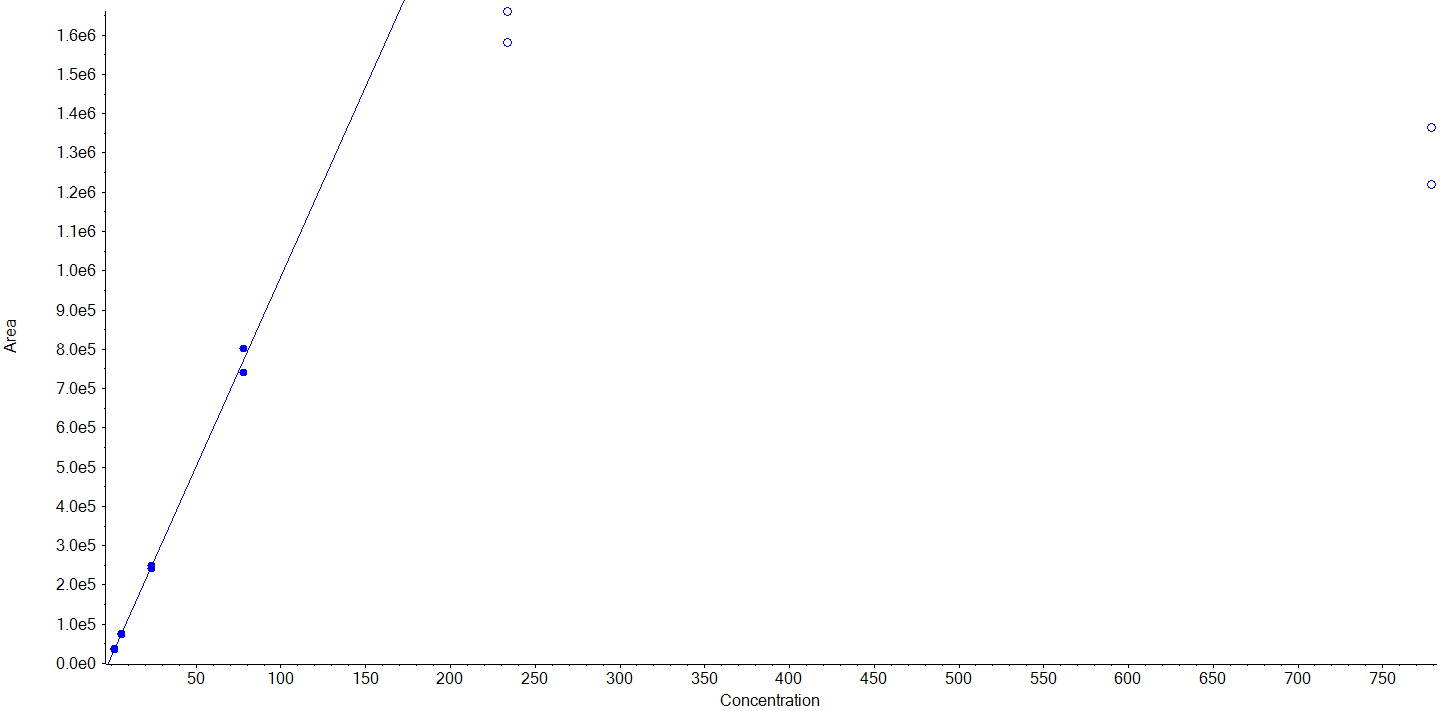 |
| Quercetin 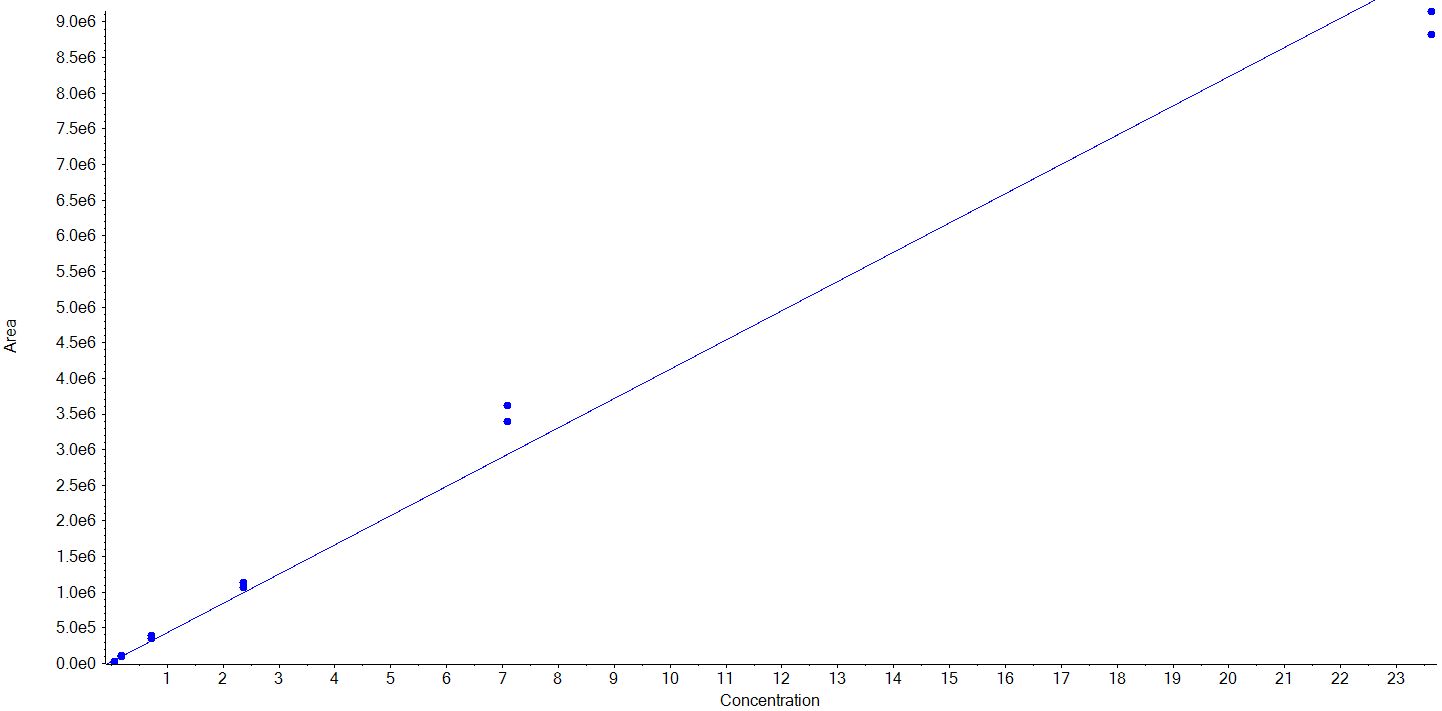 | Enterodiol 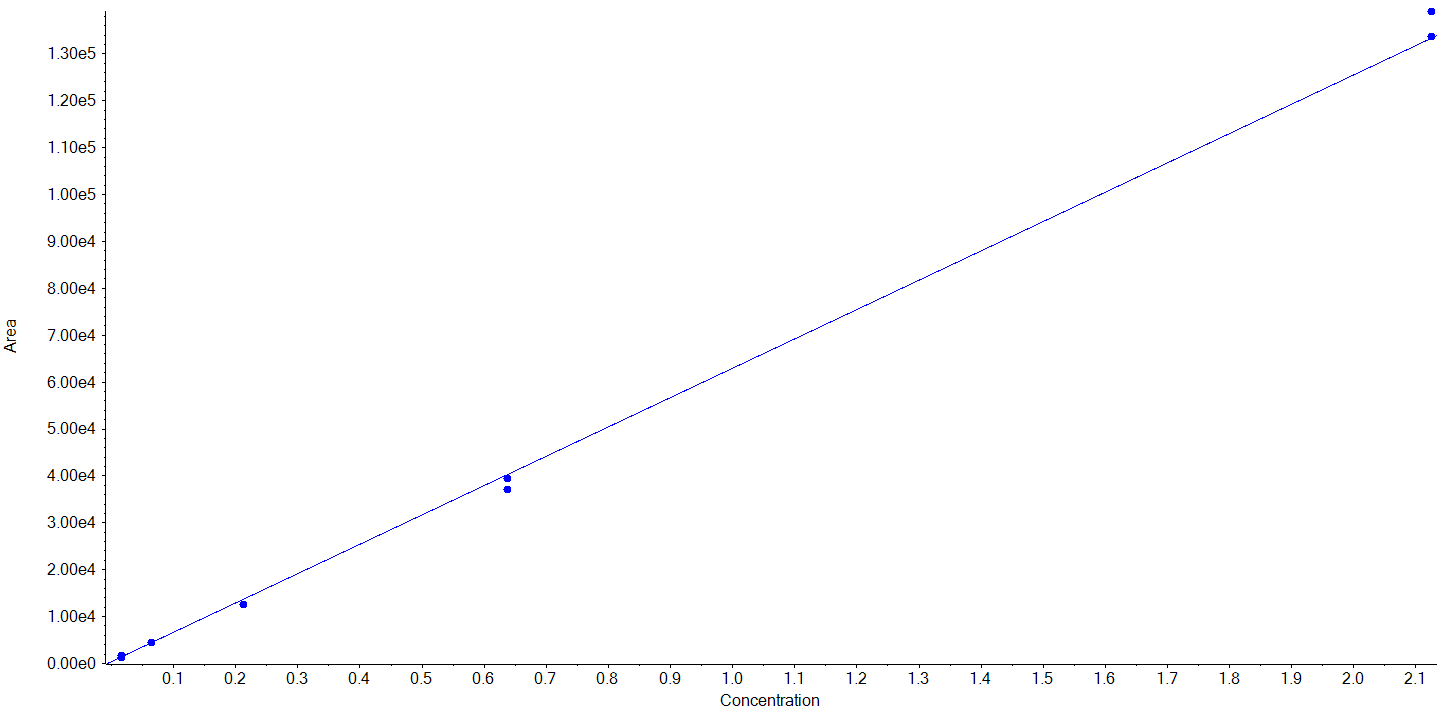 | Hesperetin 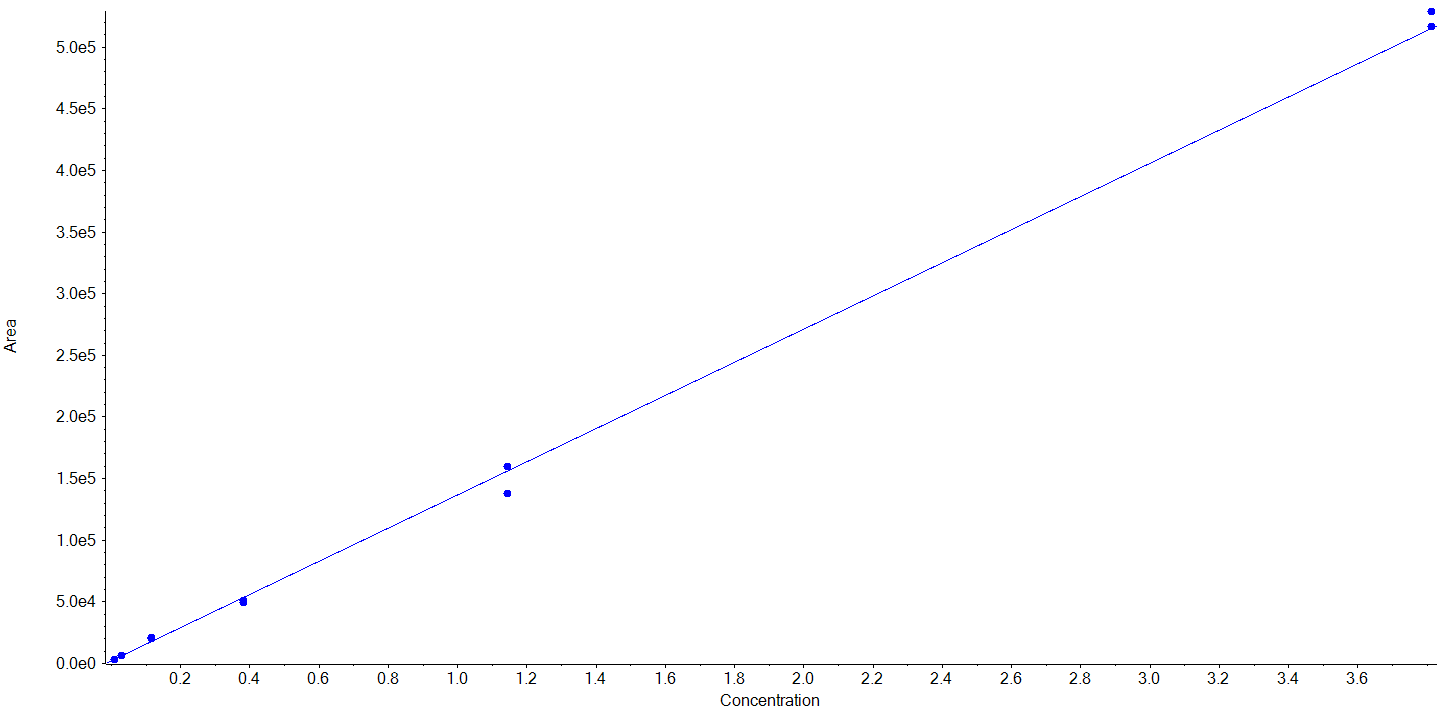 |
| (-)-Epigallocatechin 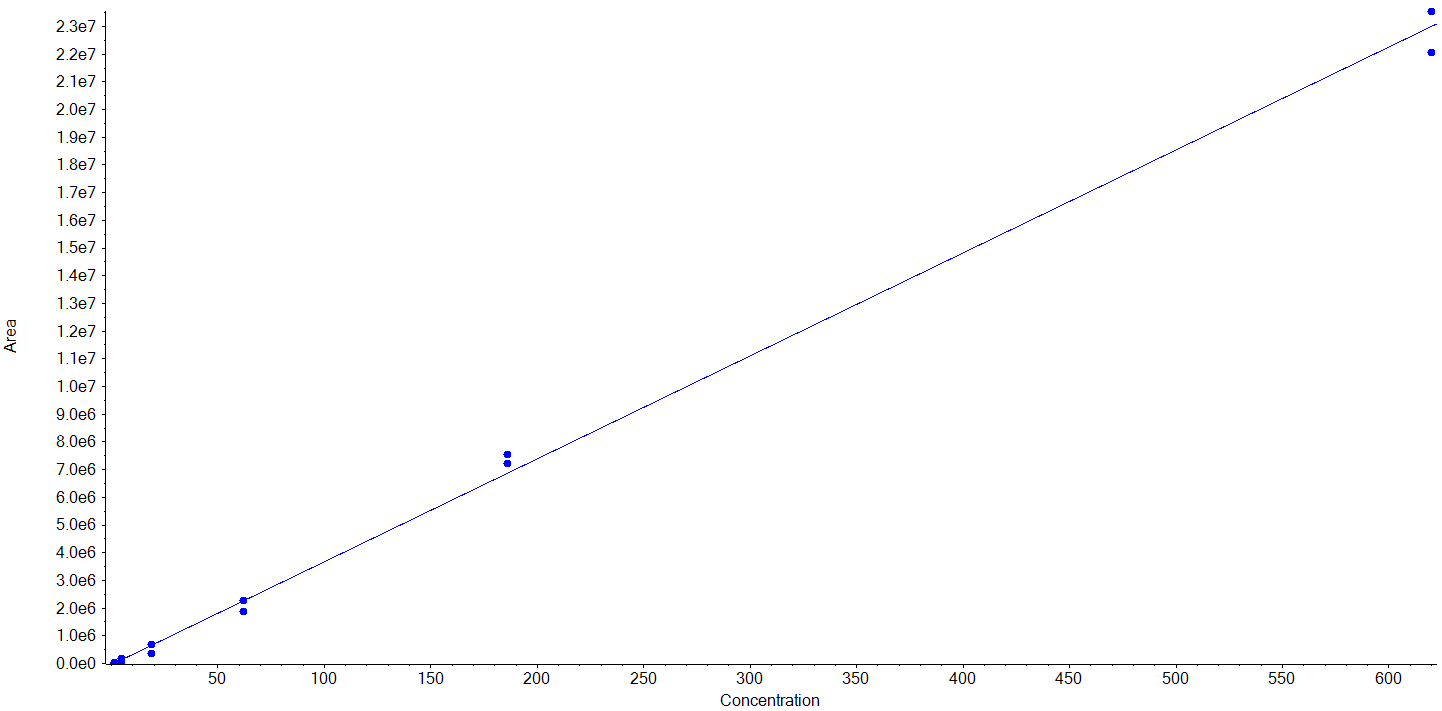 | (-)-Gallocatechin 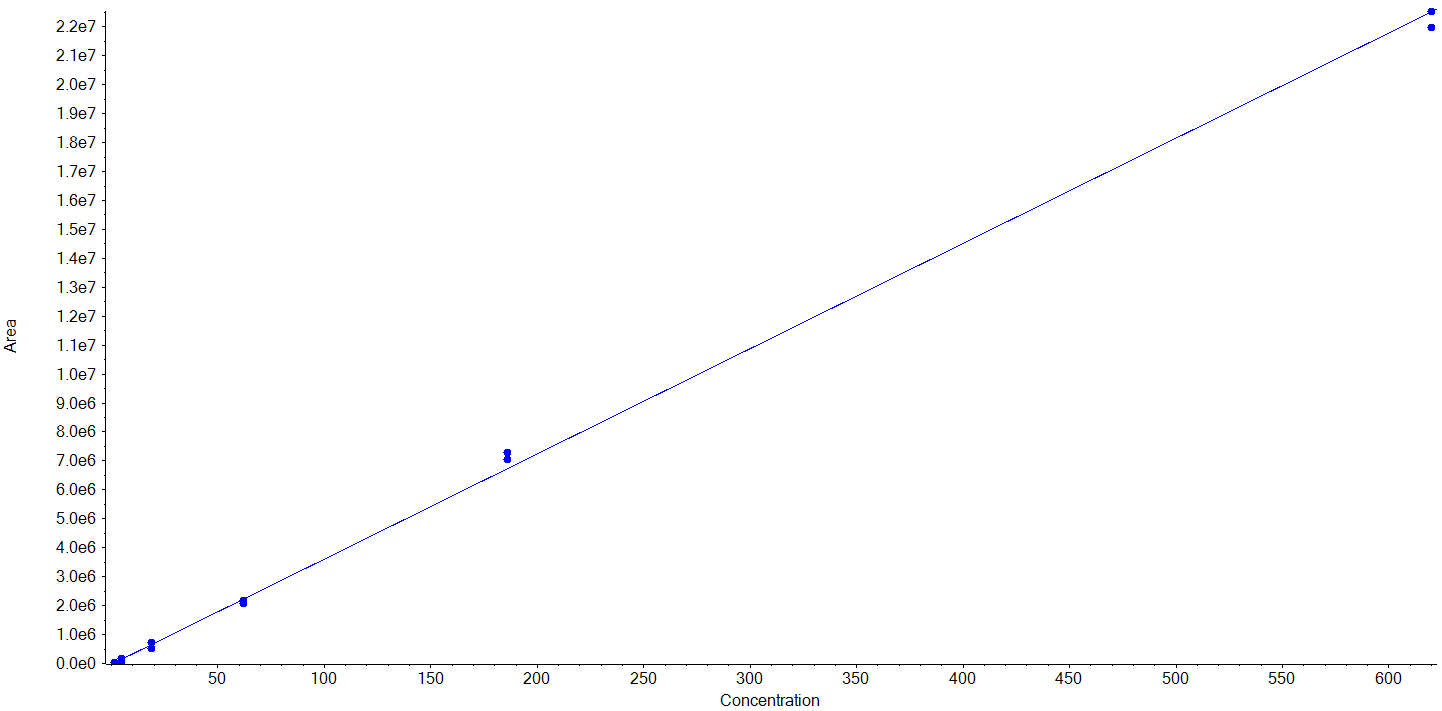 | Isorhamnetin 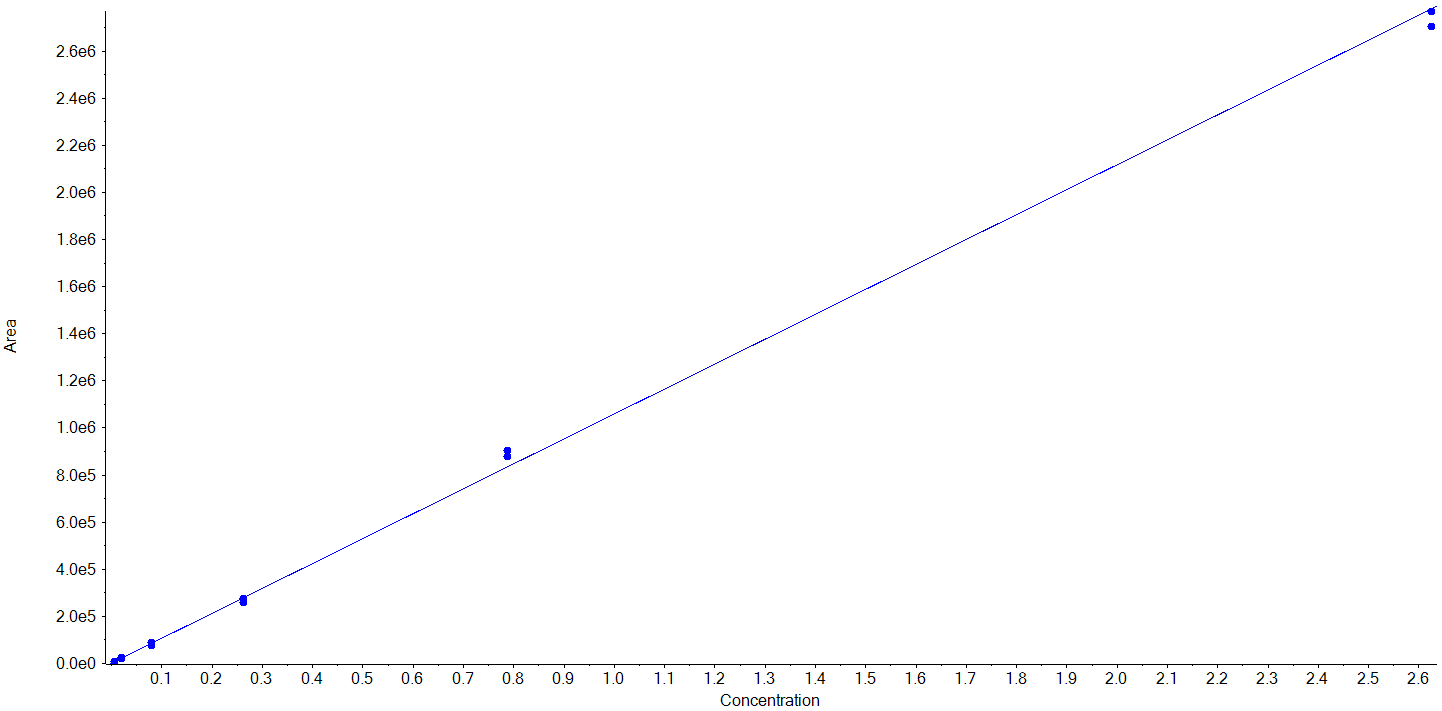 |
| 8-Prenylnaringenin 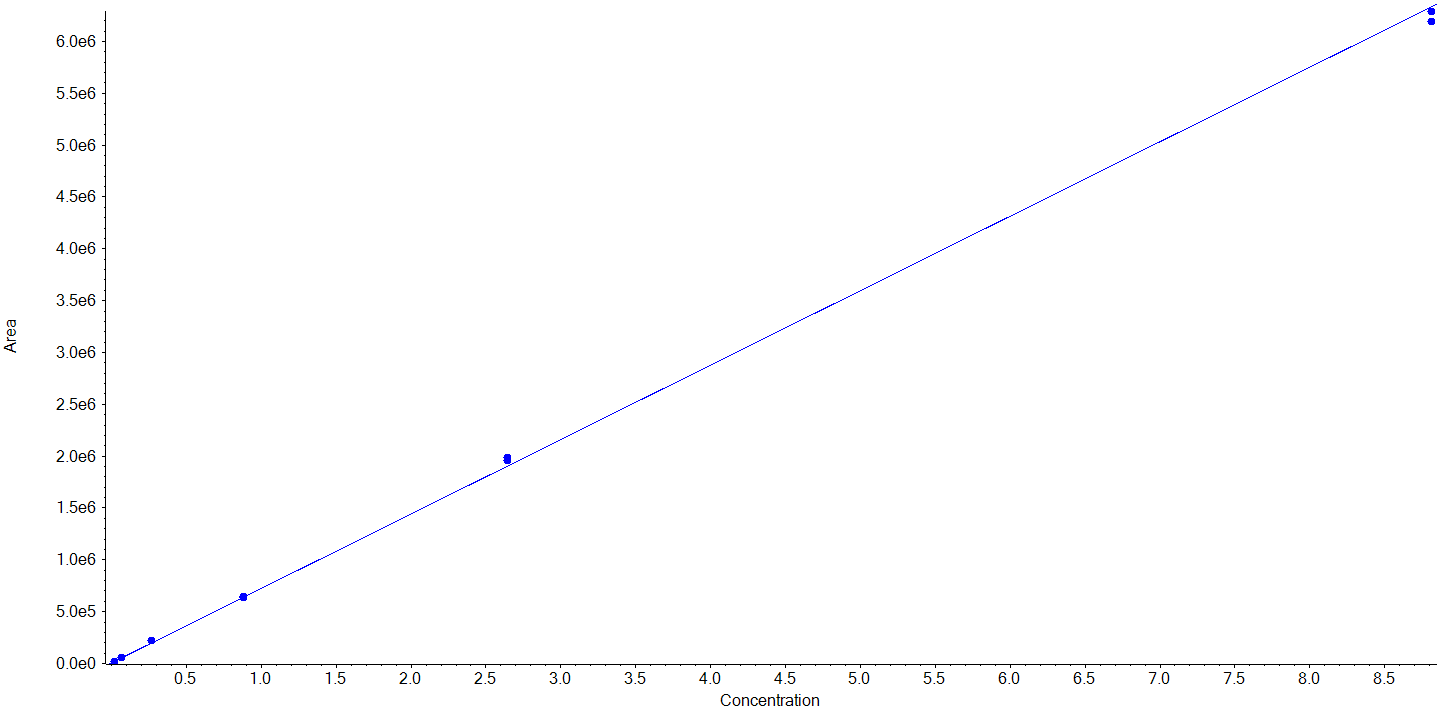 | Genistein-7-sulfate 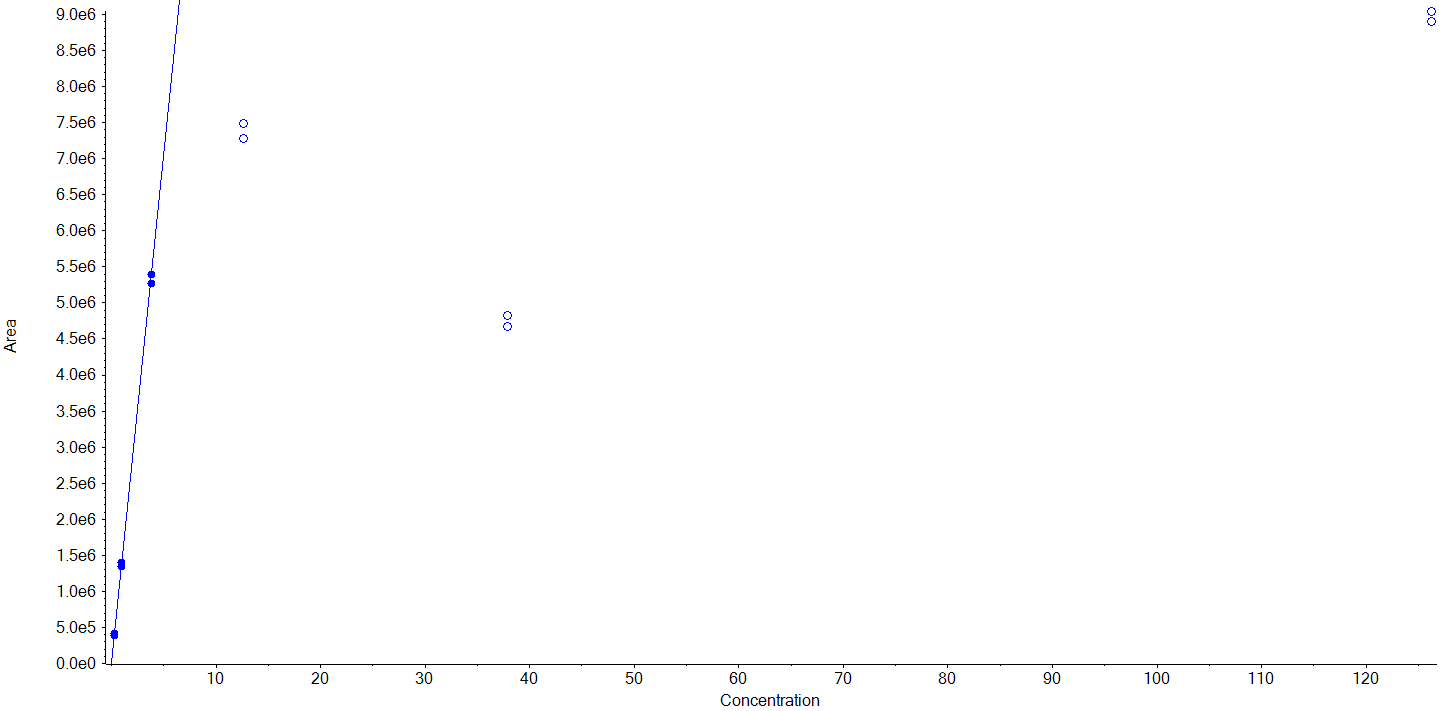 | Xanthohumol 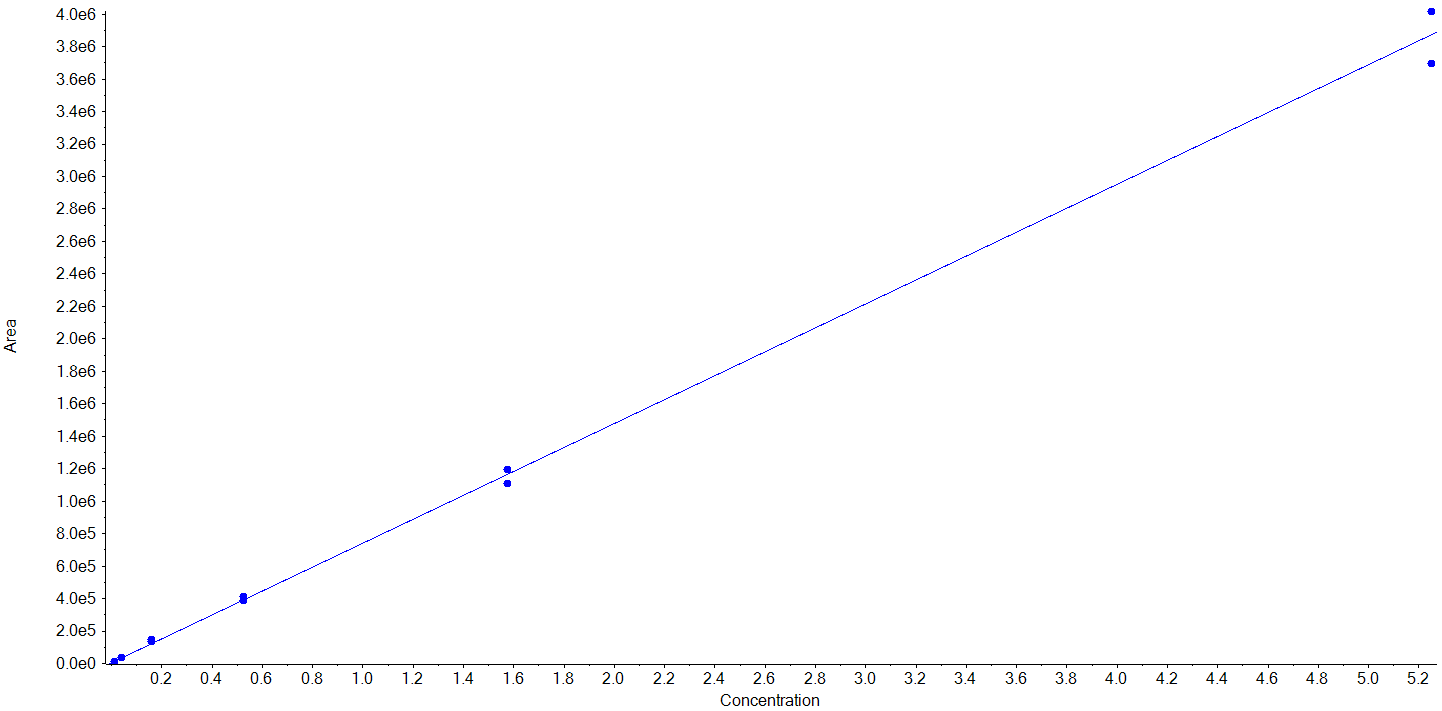 |
| Isoxanthohumol 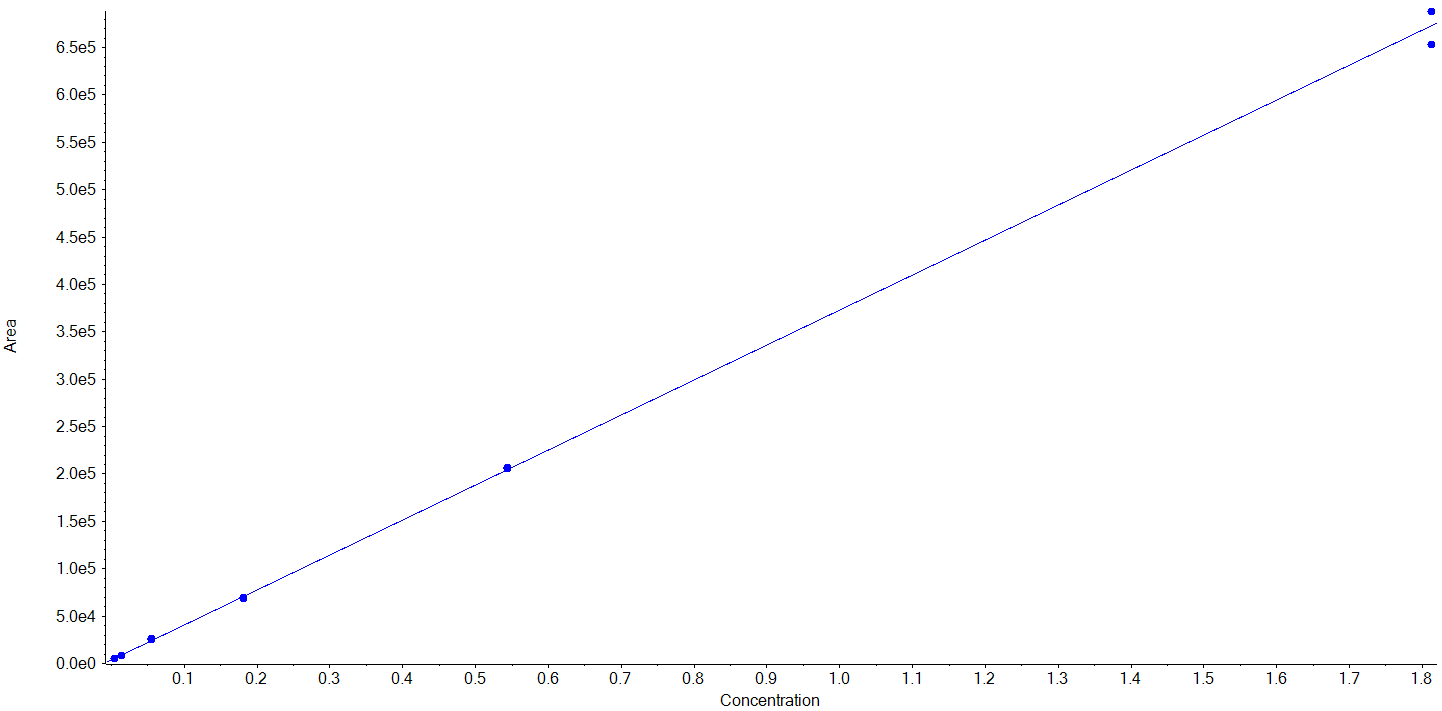 | Chlorogenic acid 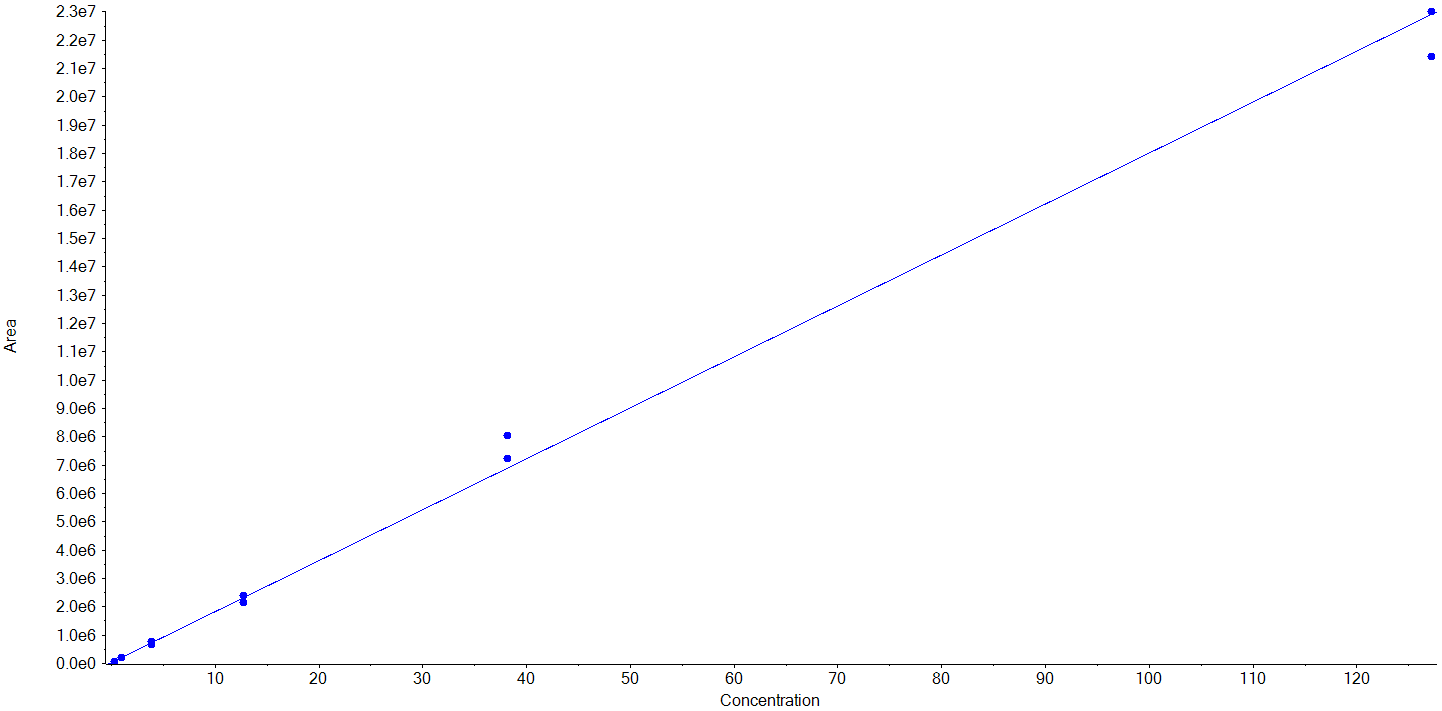 | Caffeic acid-3-β-D-glucuronide 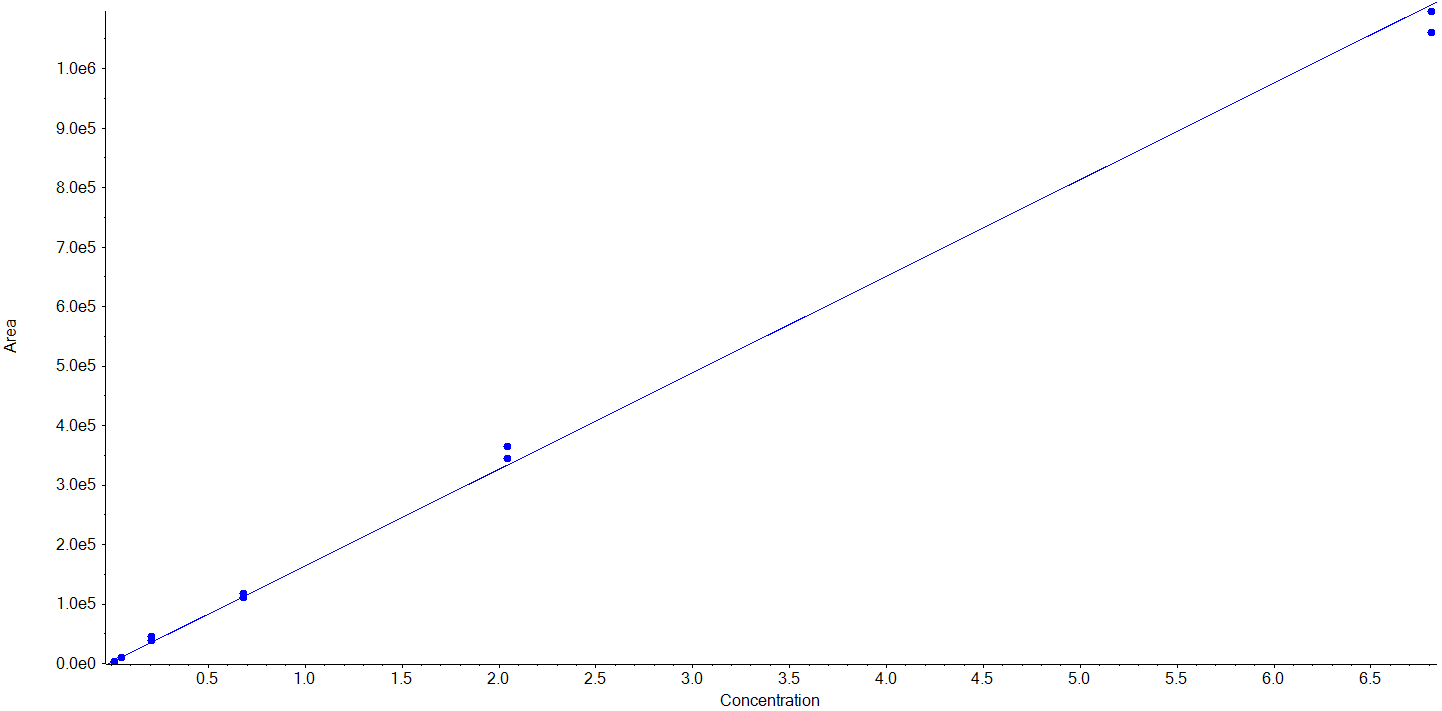 |
| Polydatin 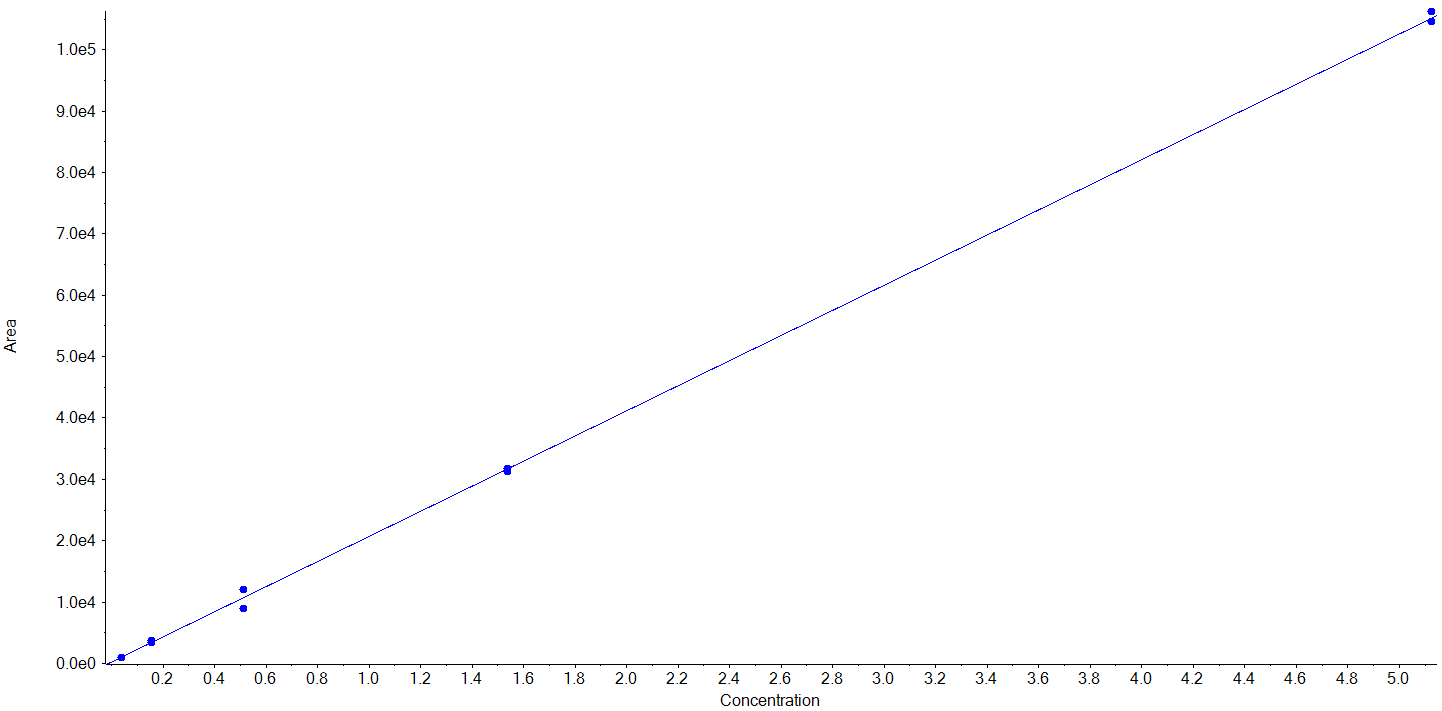 | Daidzein-7-β-D-glucuronide 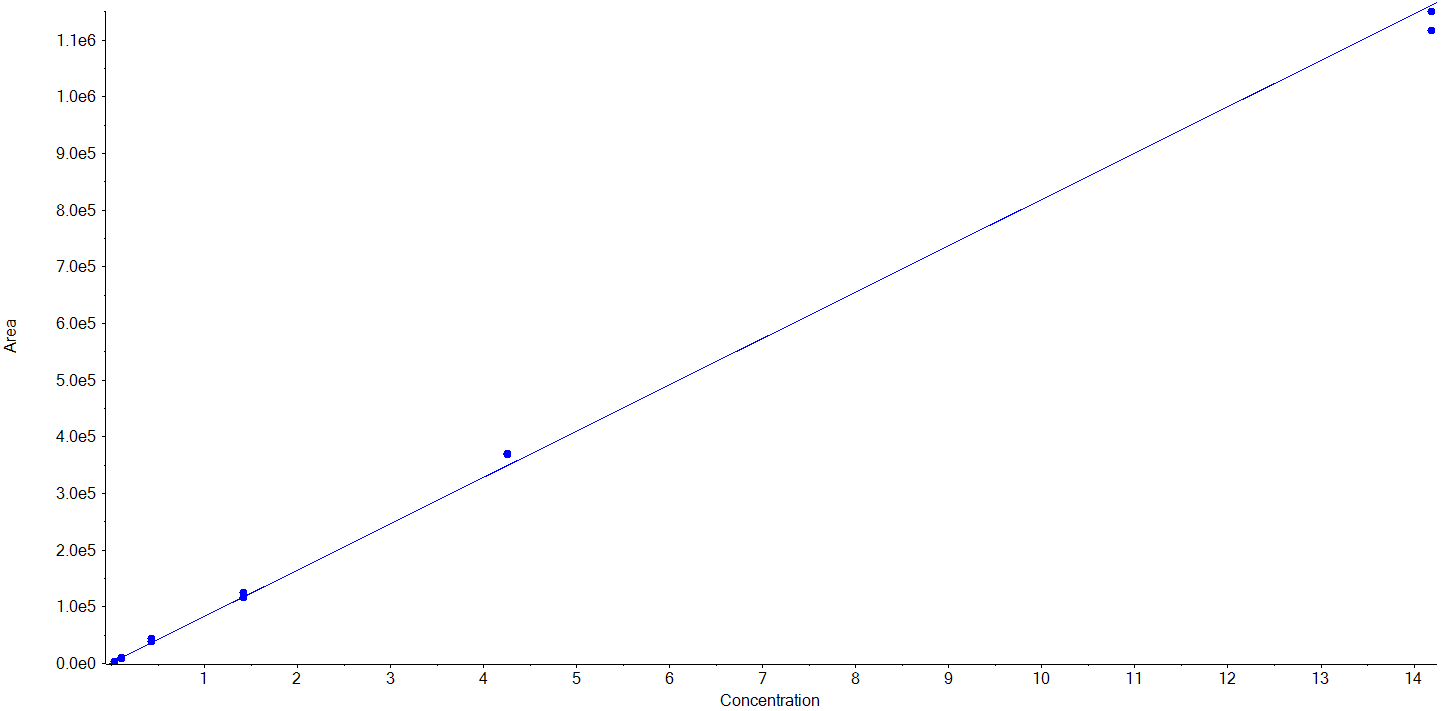 | (-)-Epicatechin gallate 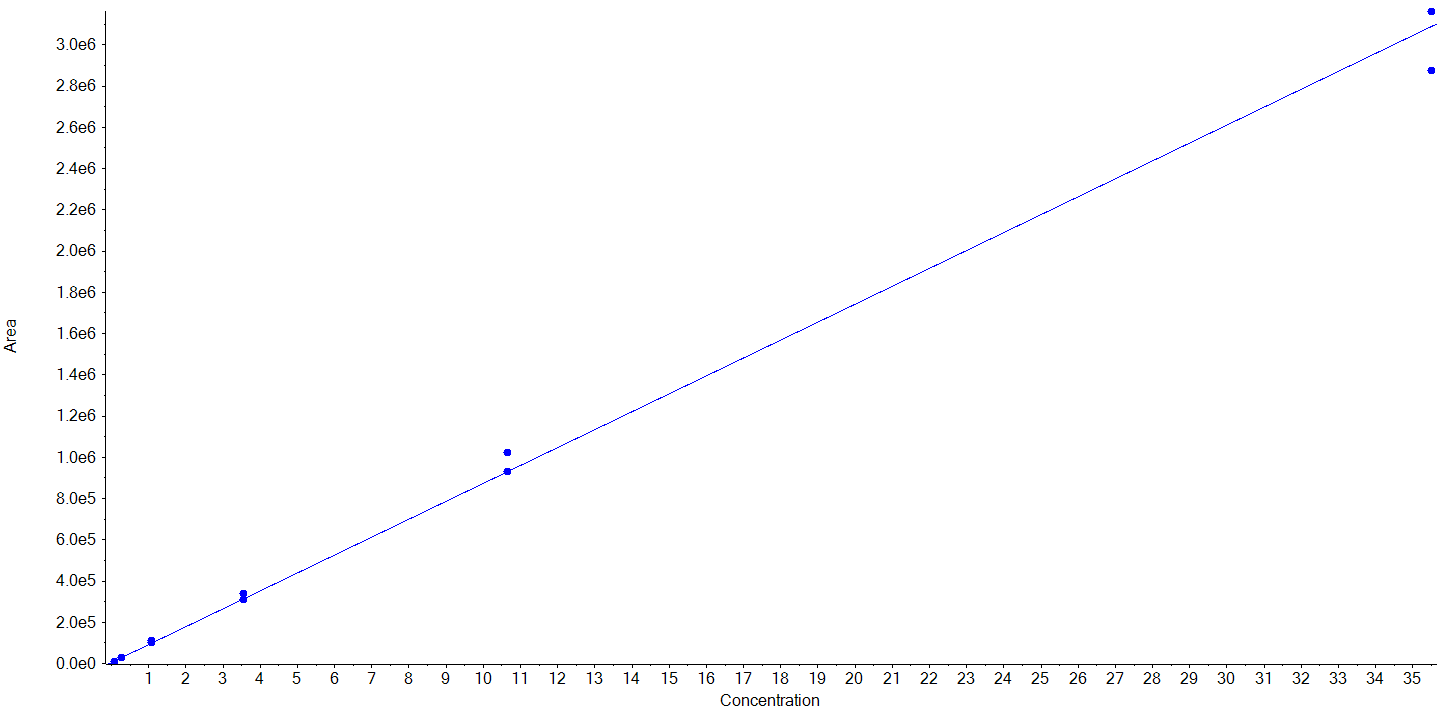 |
| Genistein-7-β-D-glucuronide 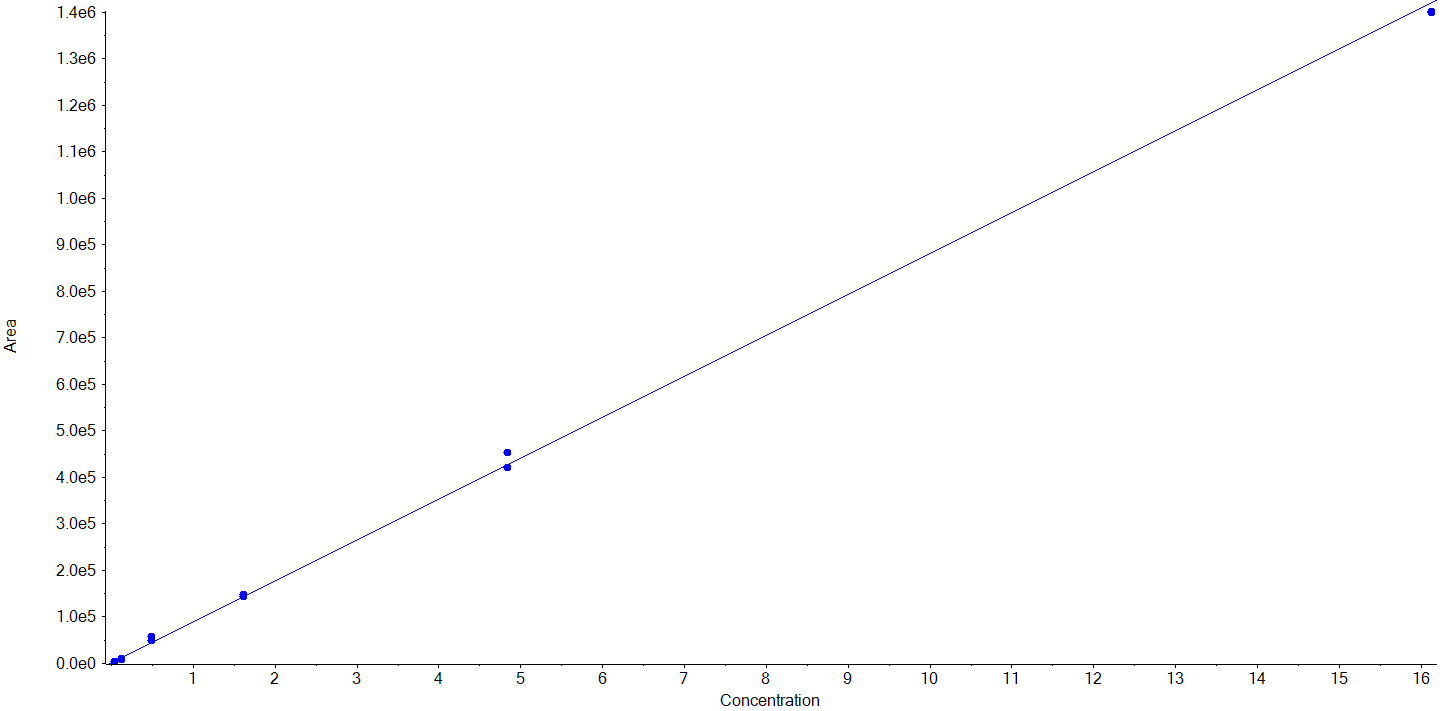 | (-)-Epigallocatechin gallate 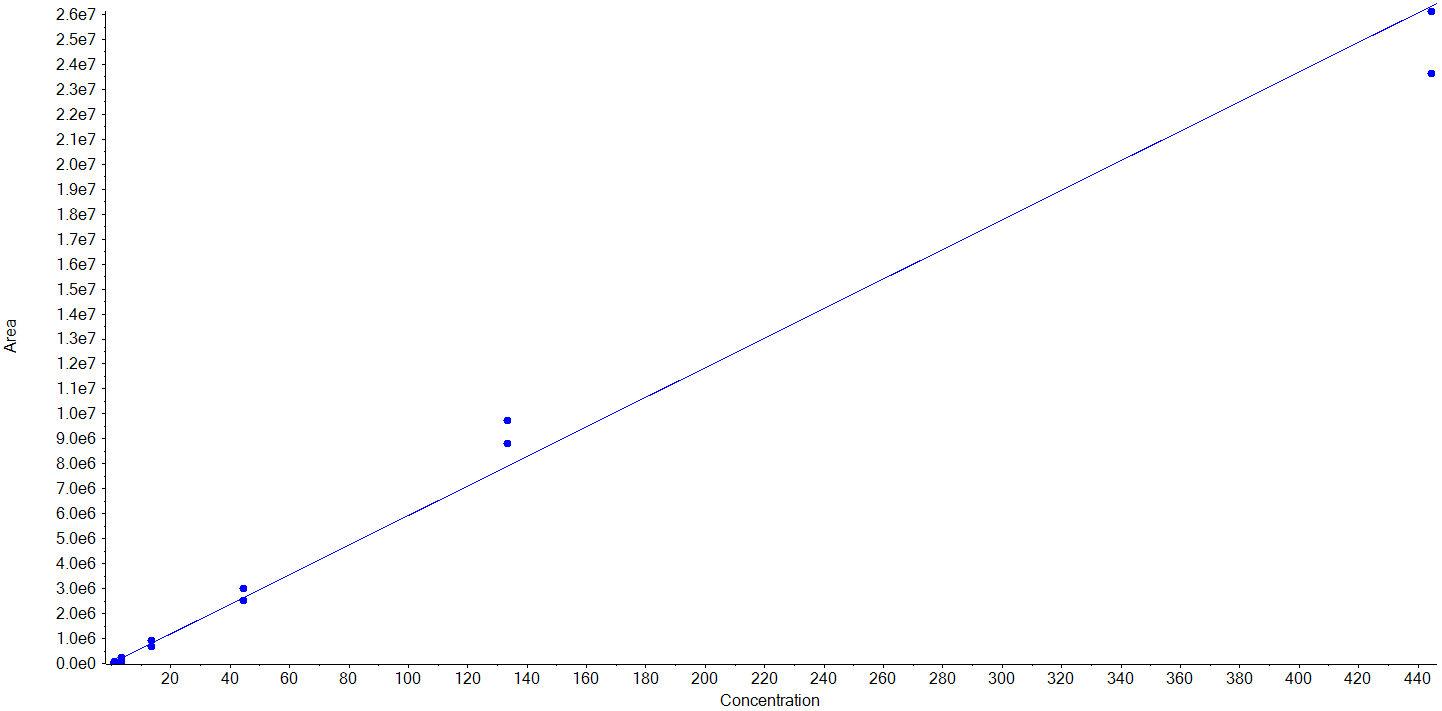 | Kaempferol-3-O-glucuronide 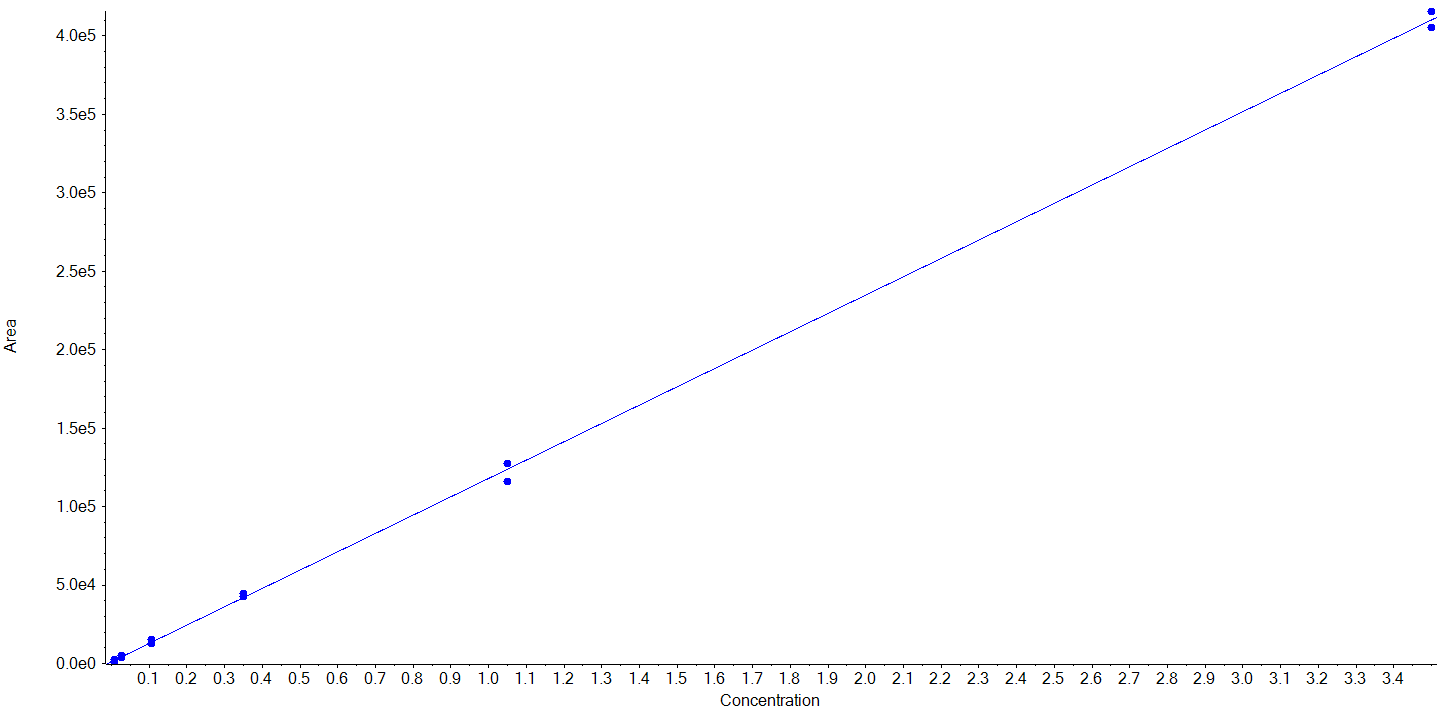 |
| Quercetin-7-O-β-D-glucuronide 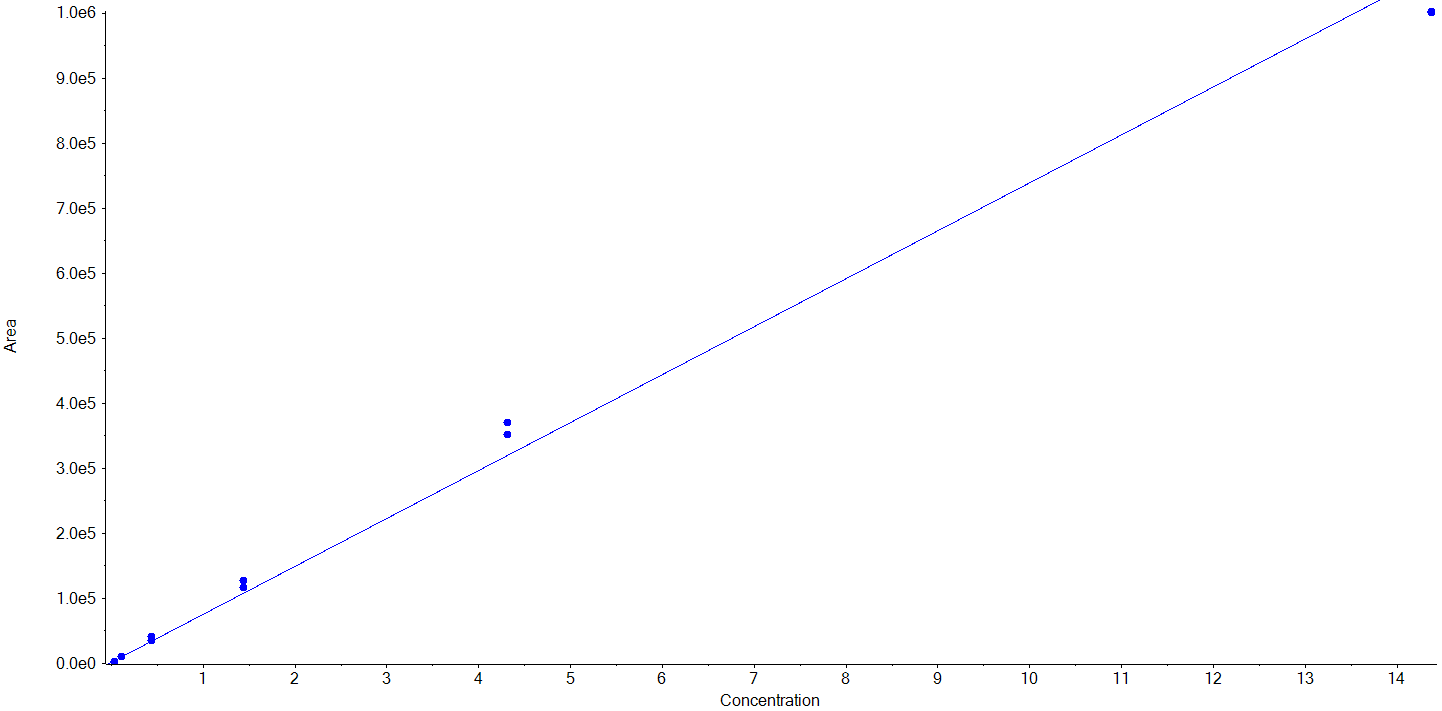 | Procyanidin A2 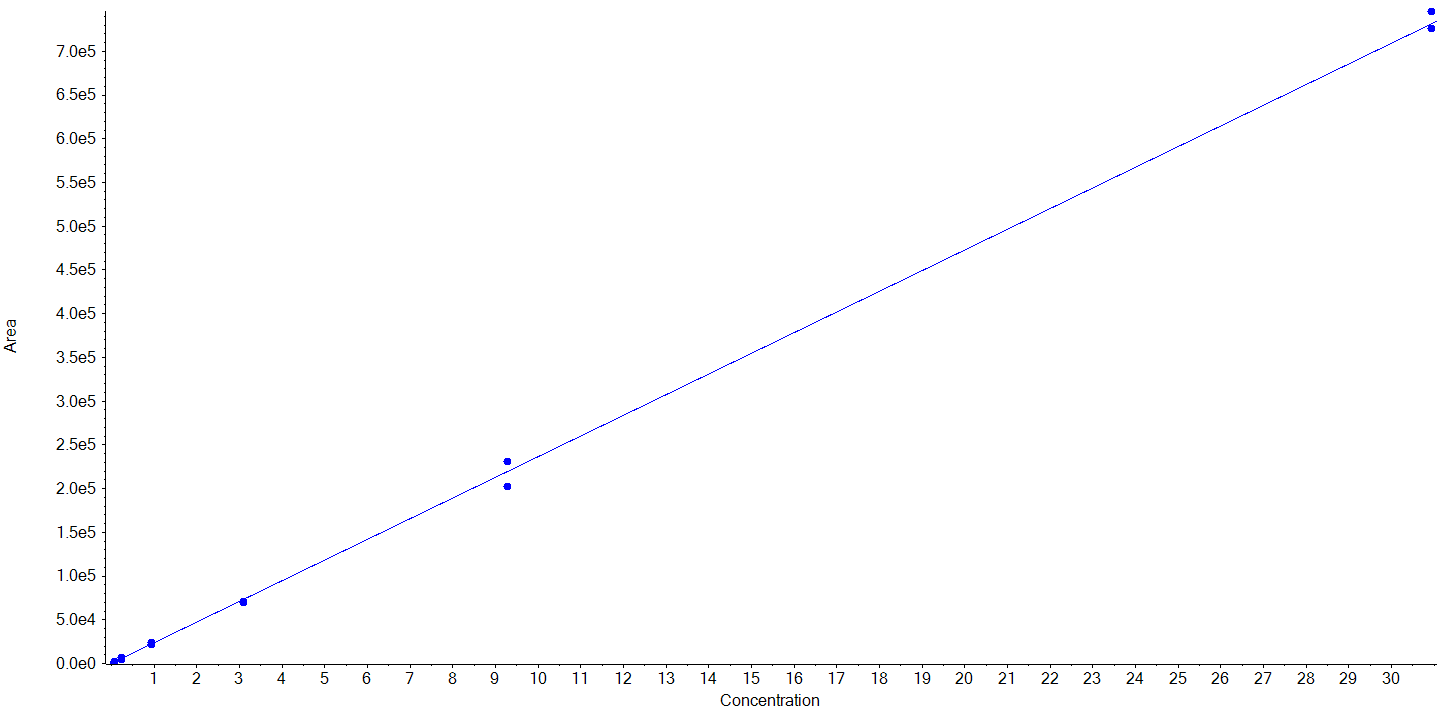 | Diosmin 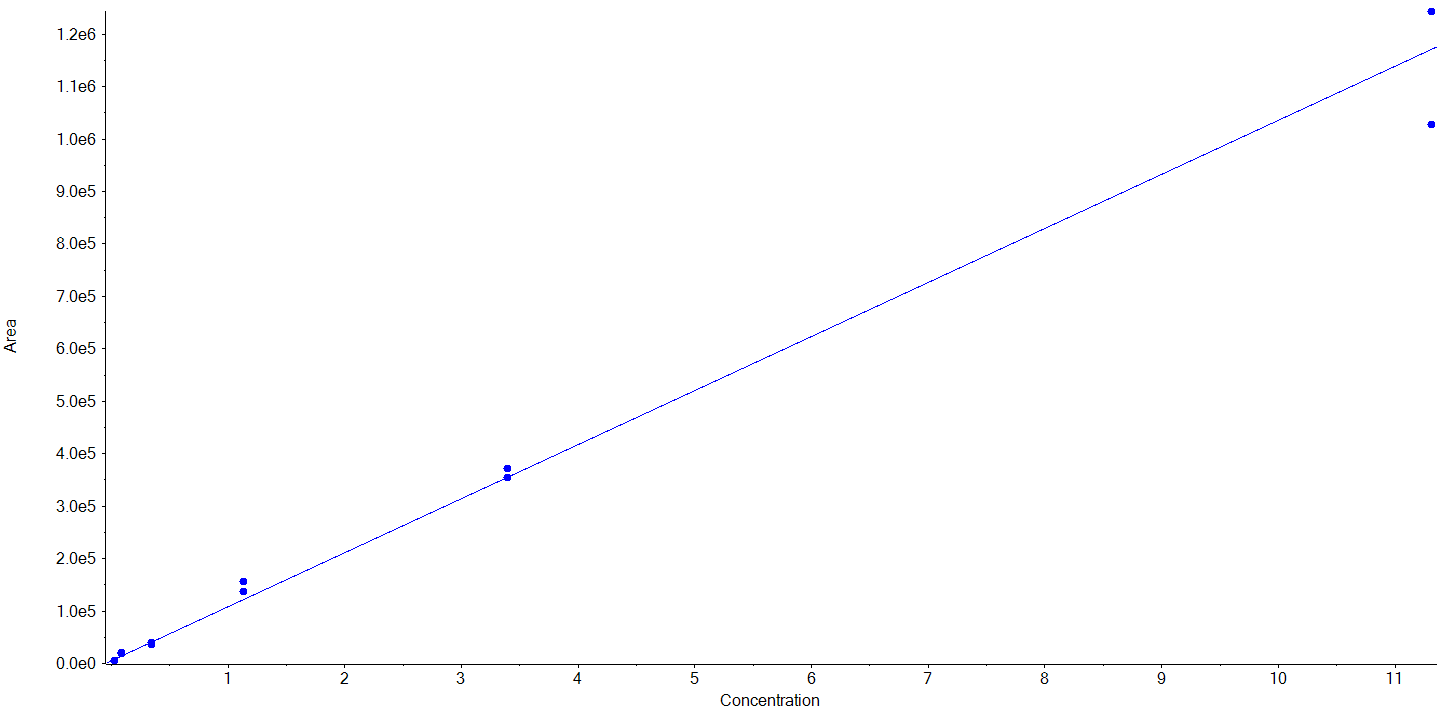 |
| (+)-Rutin 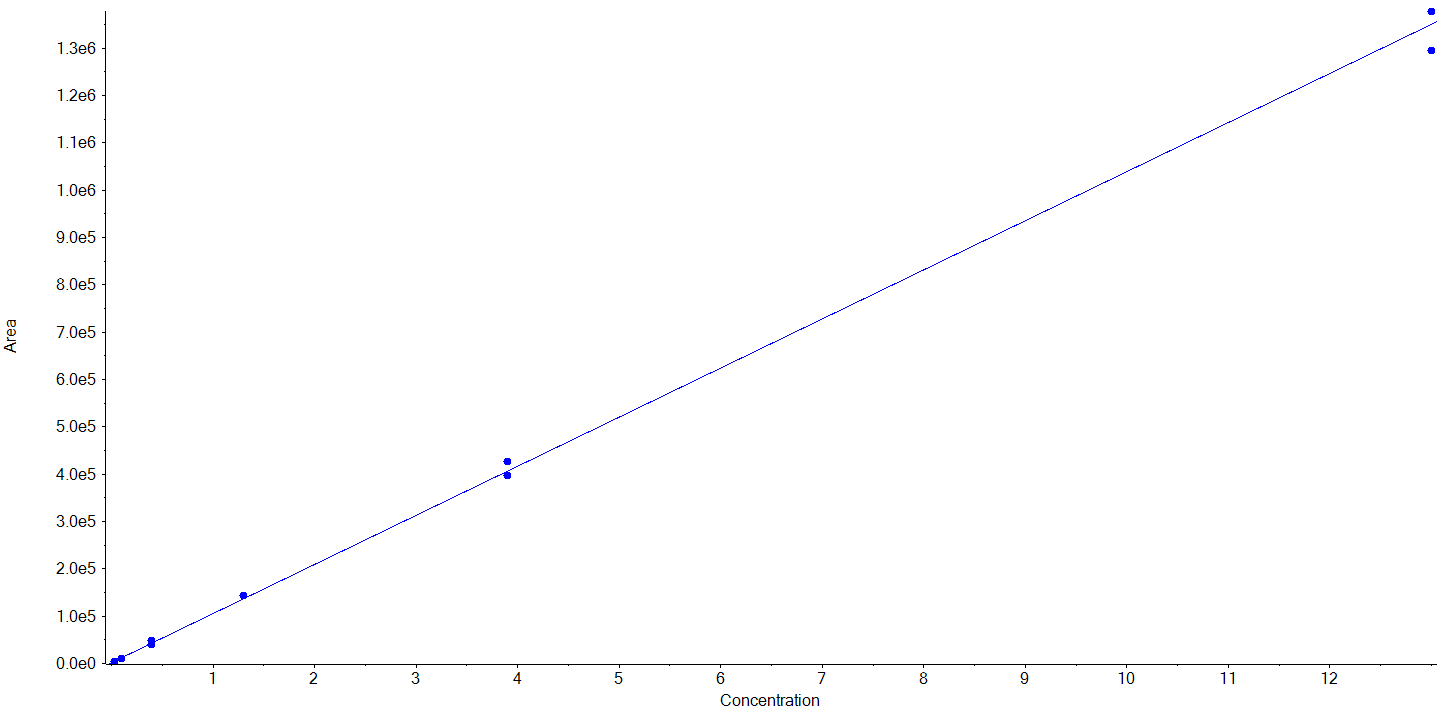 | Hesperidin 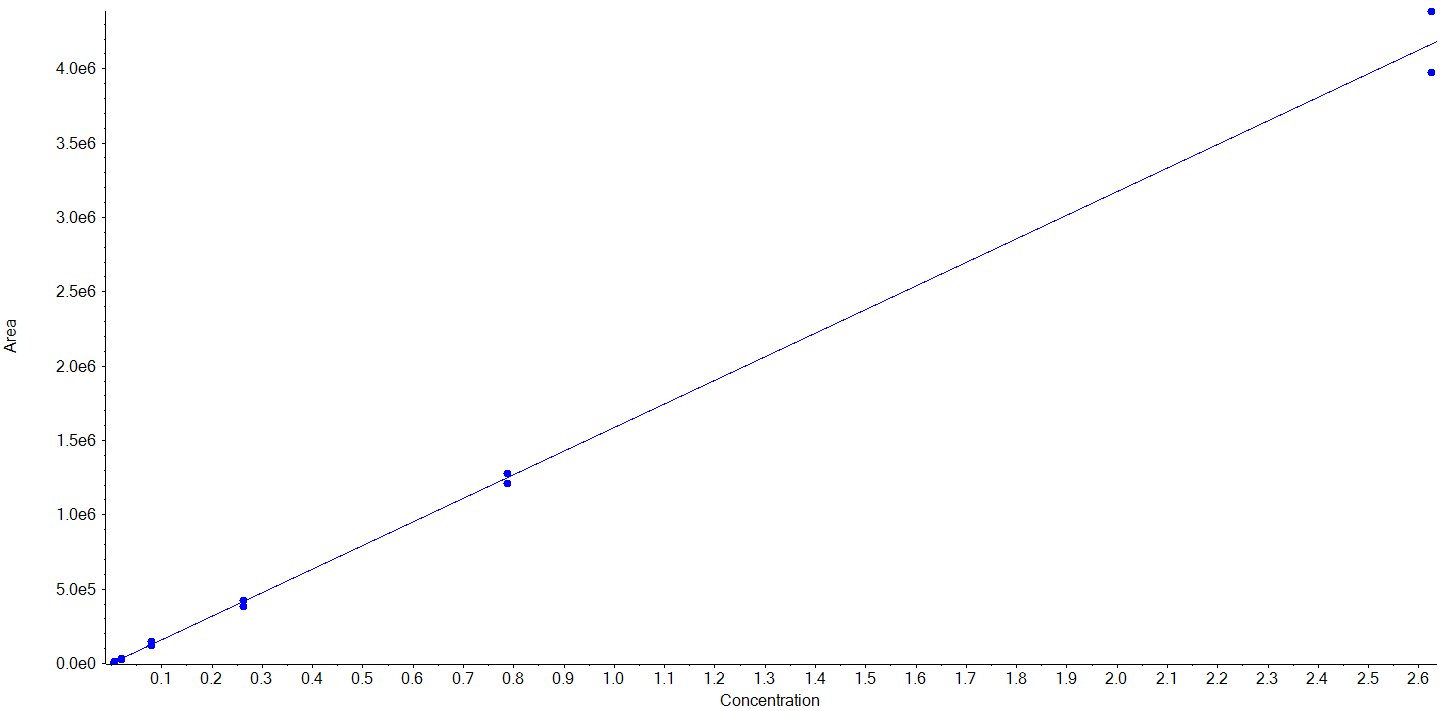 | Neohesperidin dihydrochalcone 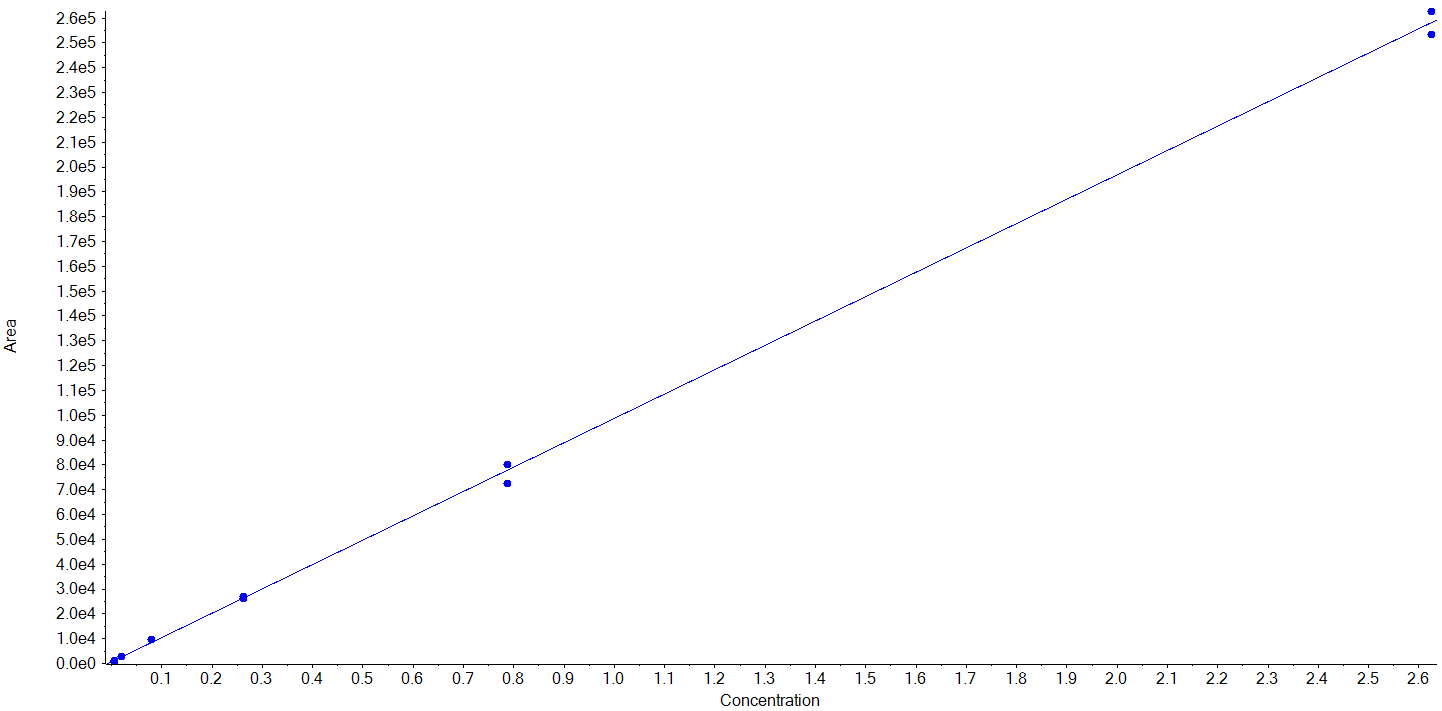 |
| Biochanin A 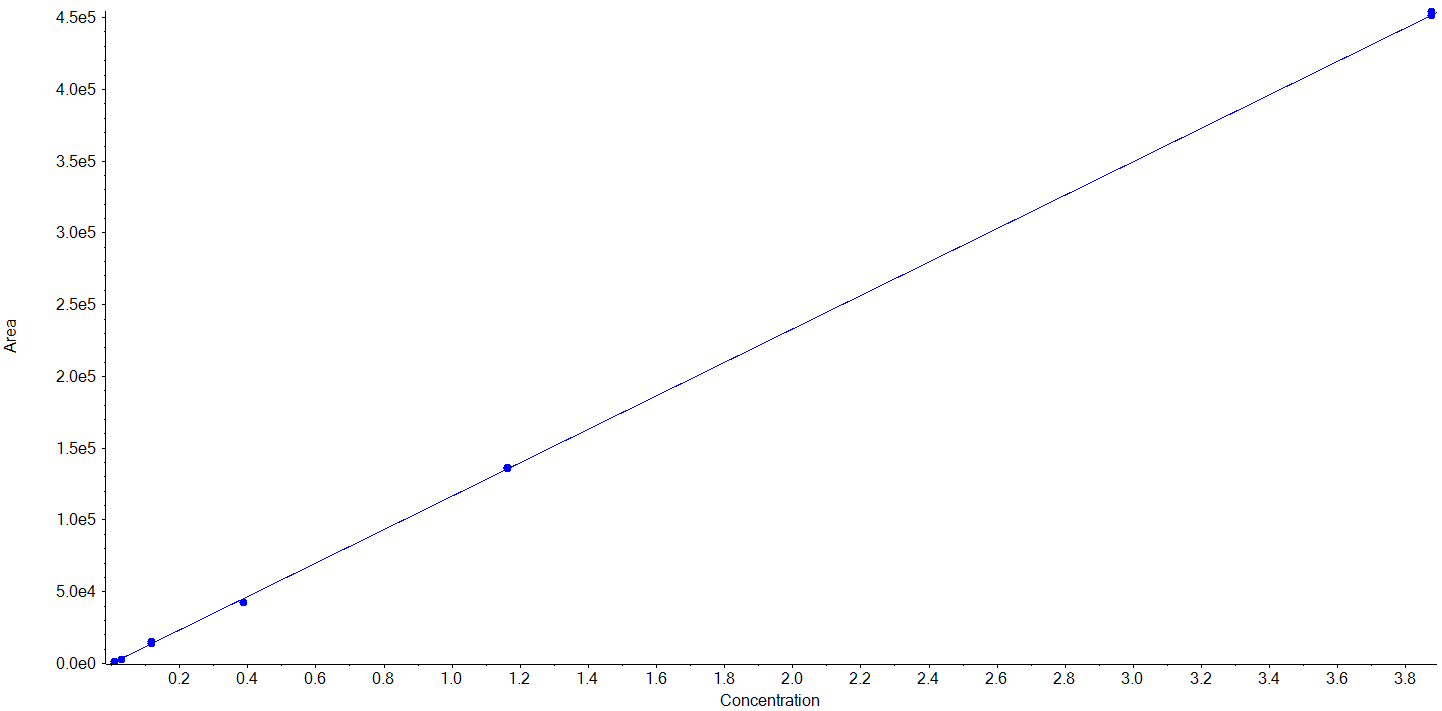 | 2,6-Dimethoxyphenol 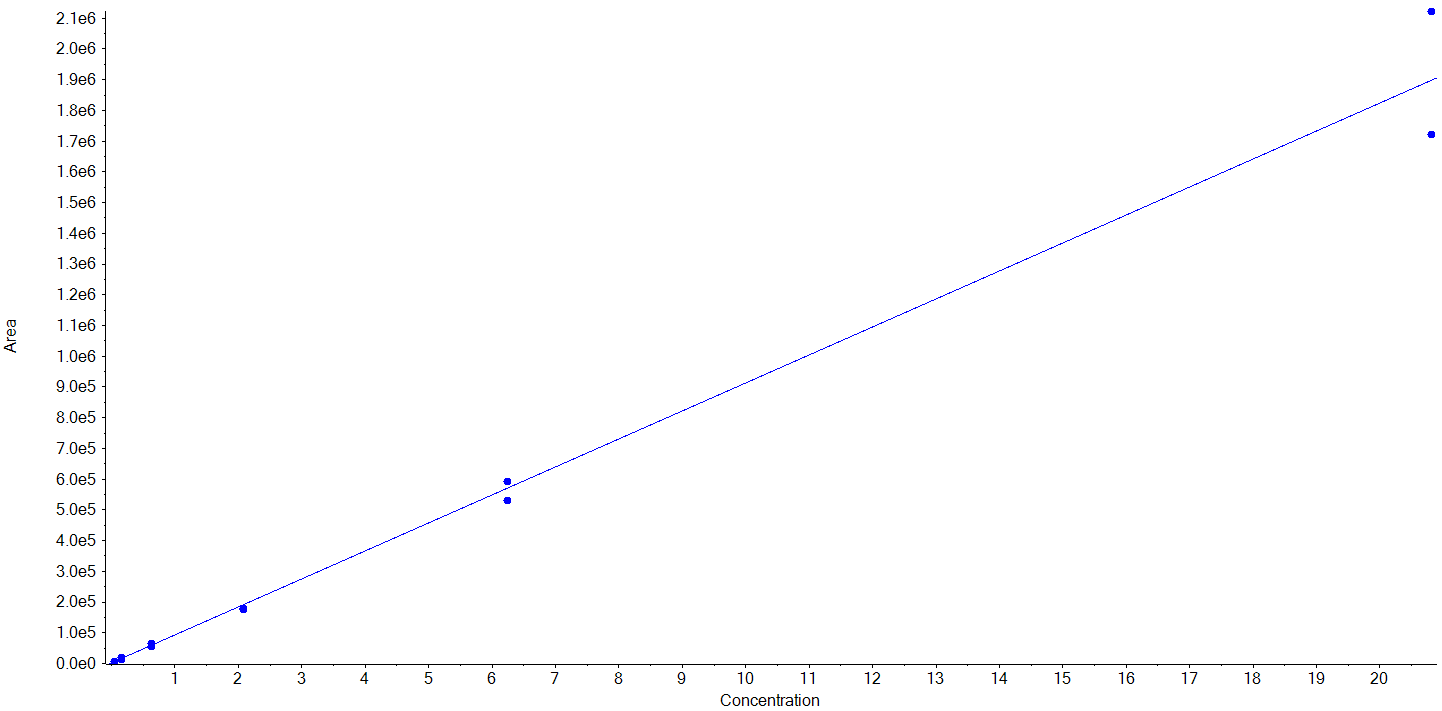 | Eugenol 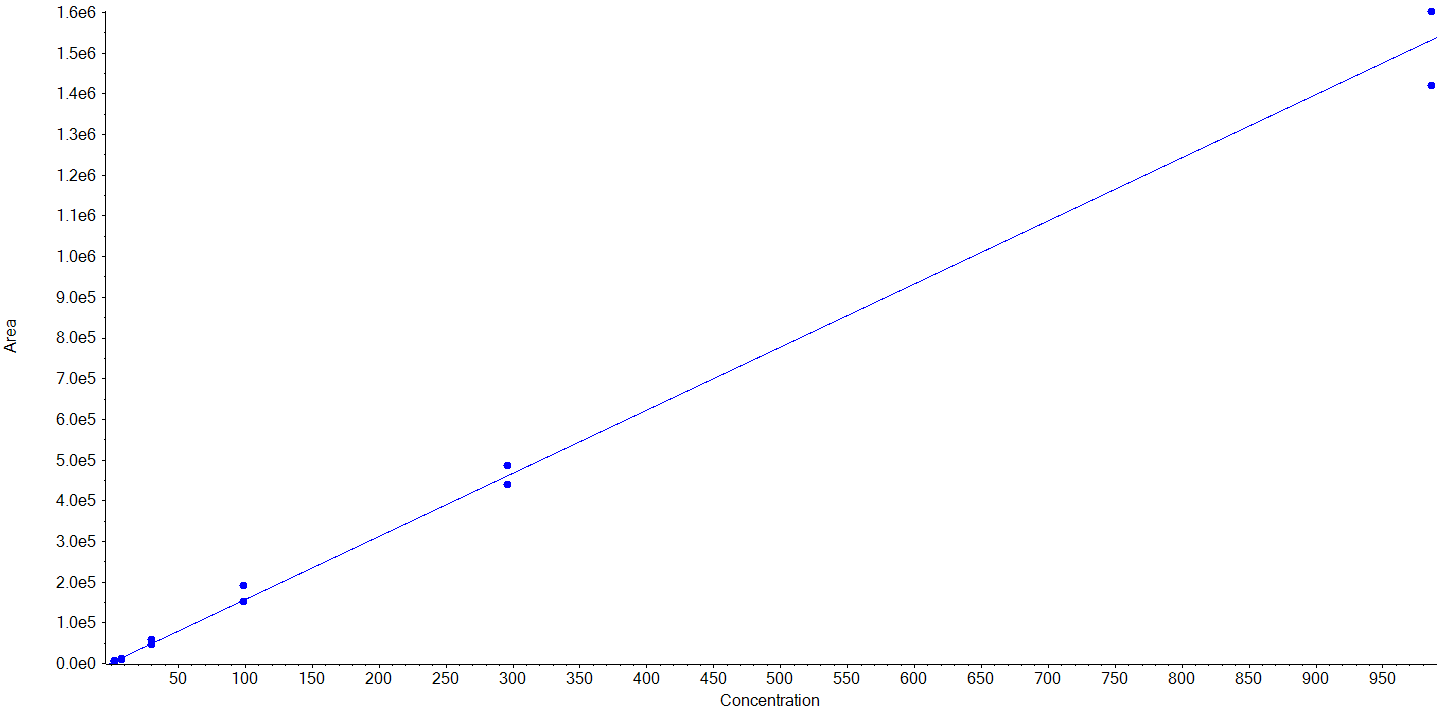 |
| Dihydroresveratrol 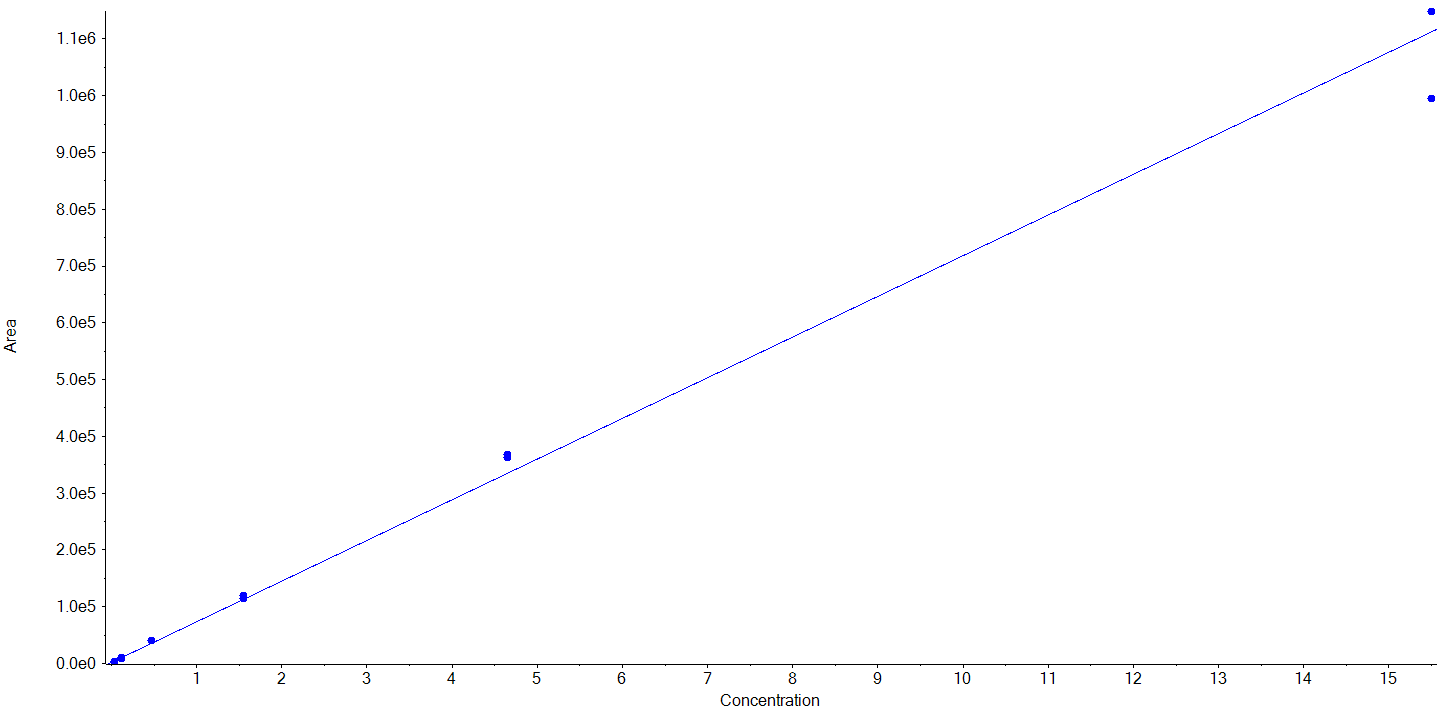 | Pterostilbene 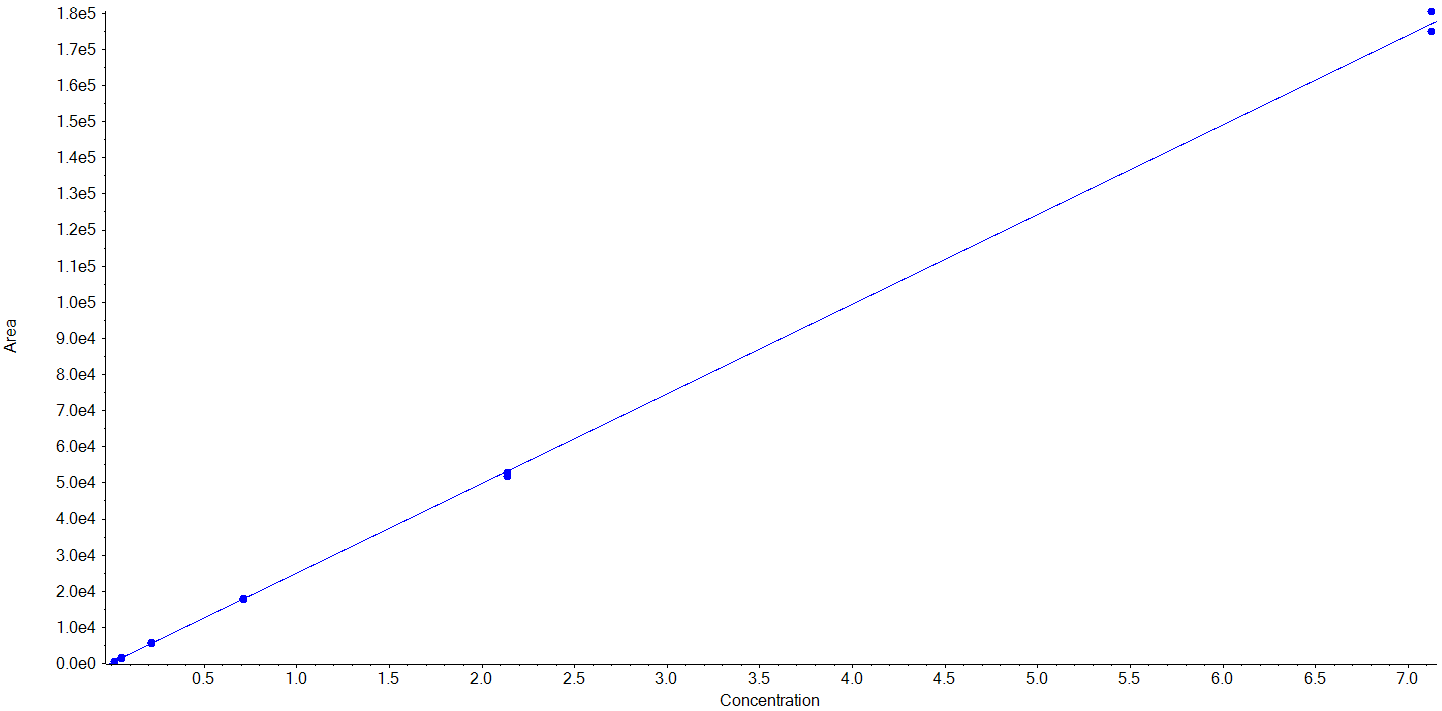 | Cyanidin 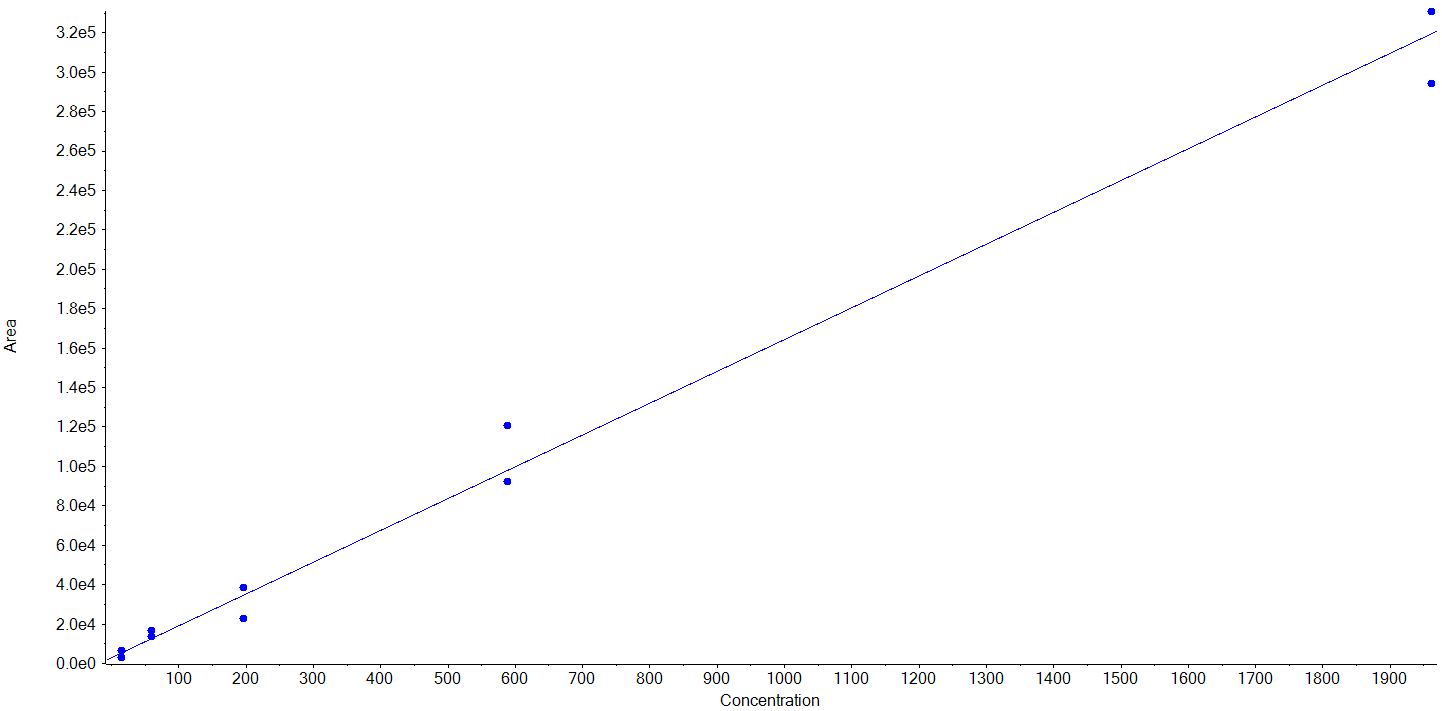 |
| Cyanidin-3-O-glucoside 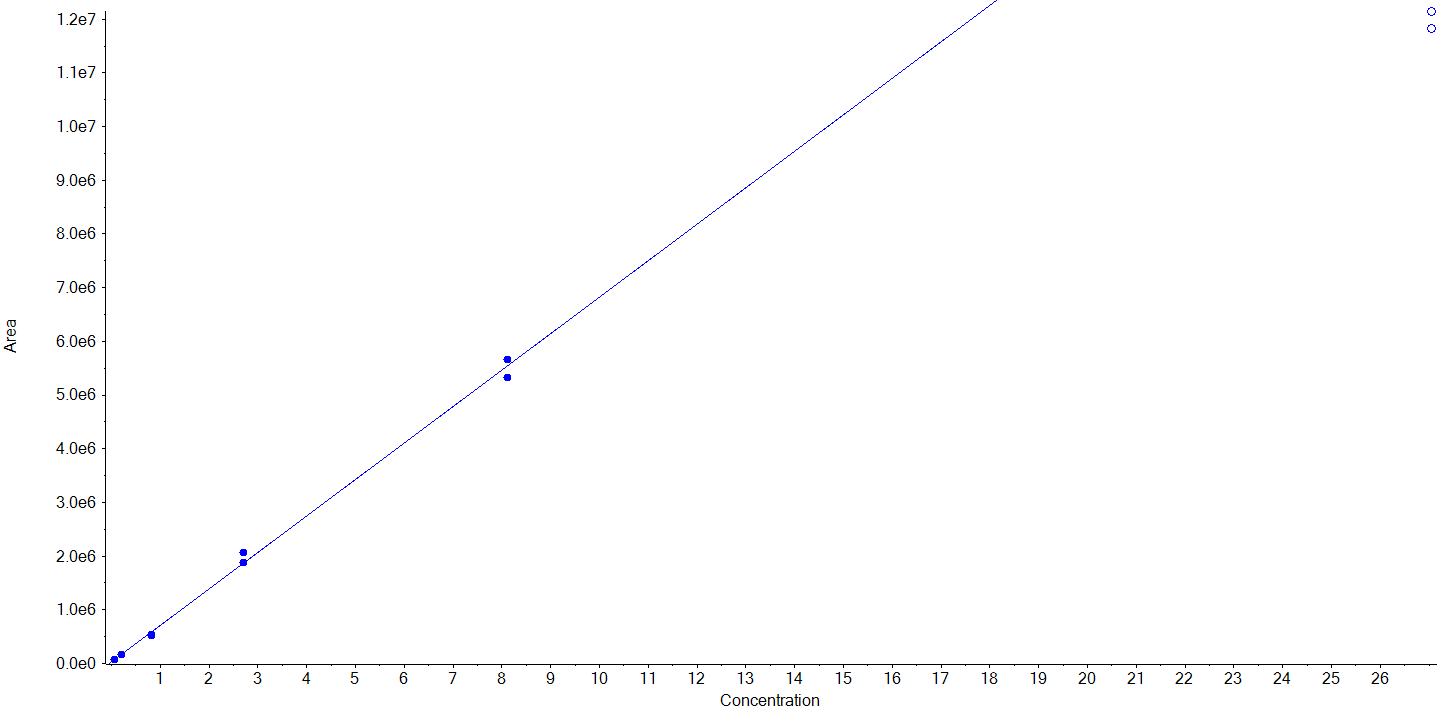 | Delphinidin-3-O-glucoside 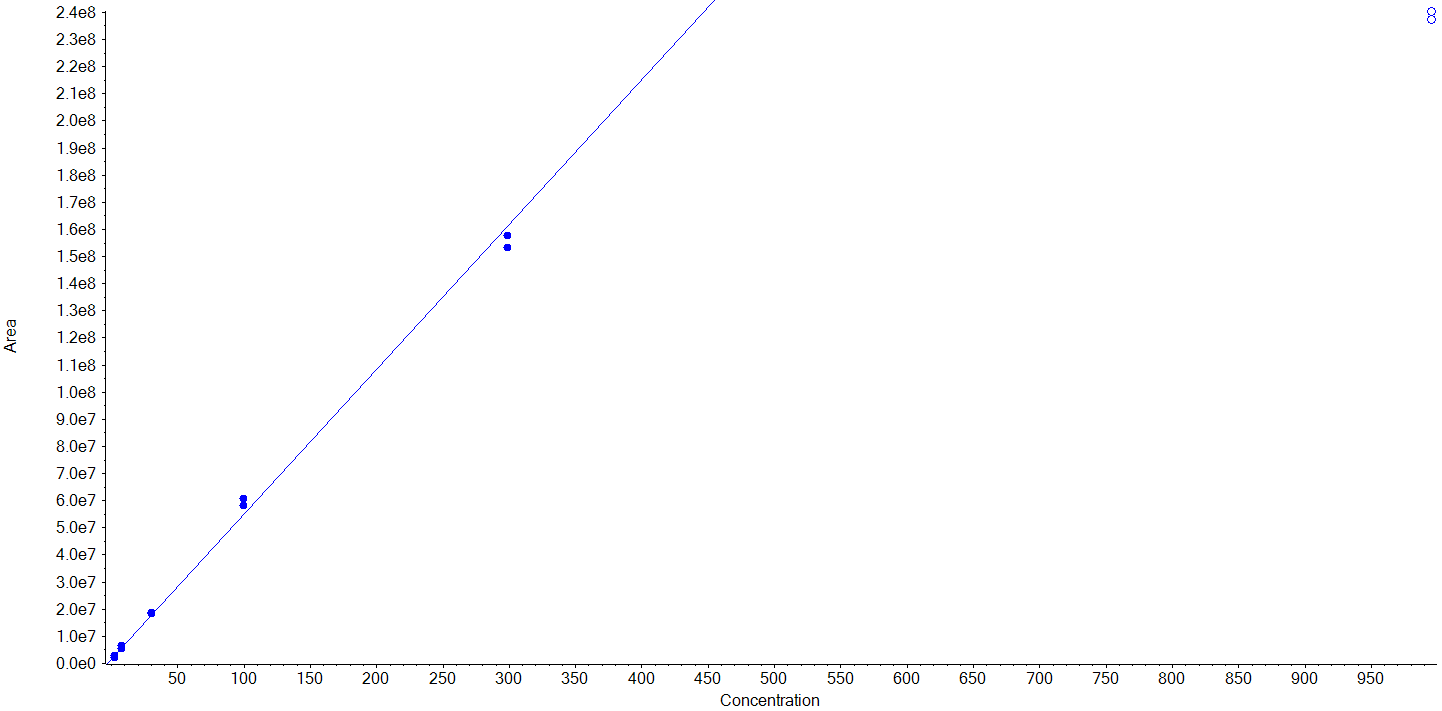 | Procyanidin B2 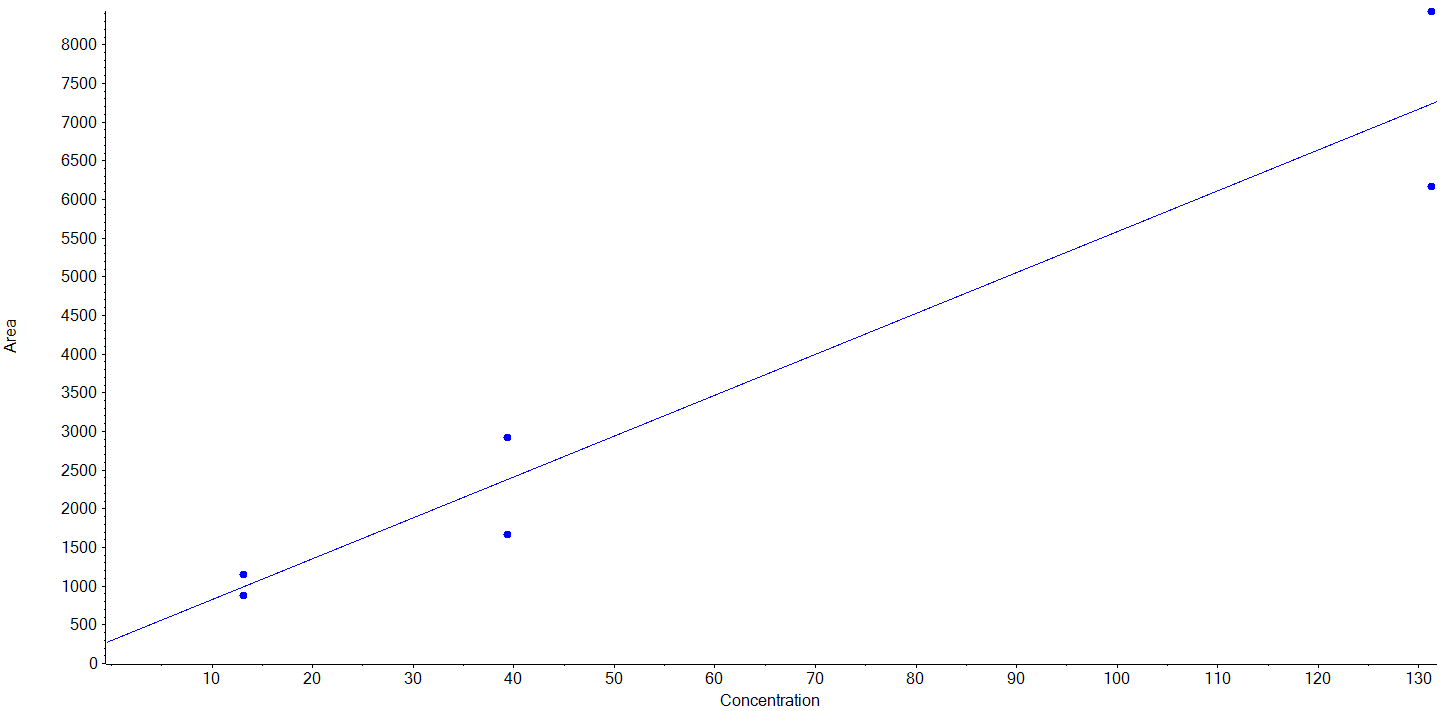 |
| Procyanidin B1 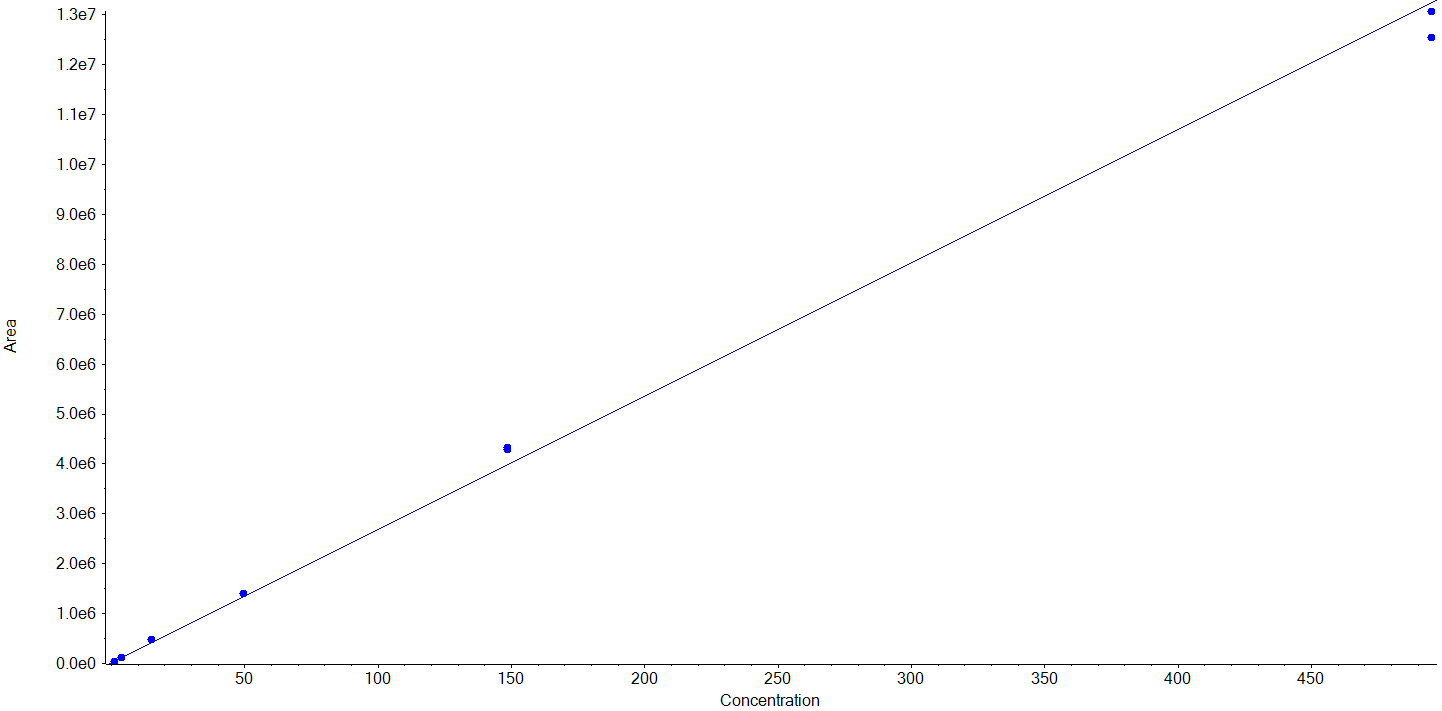 | Naringin 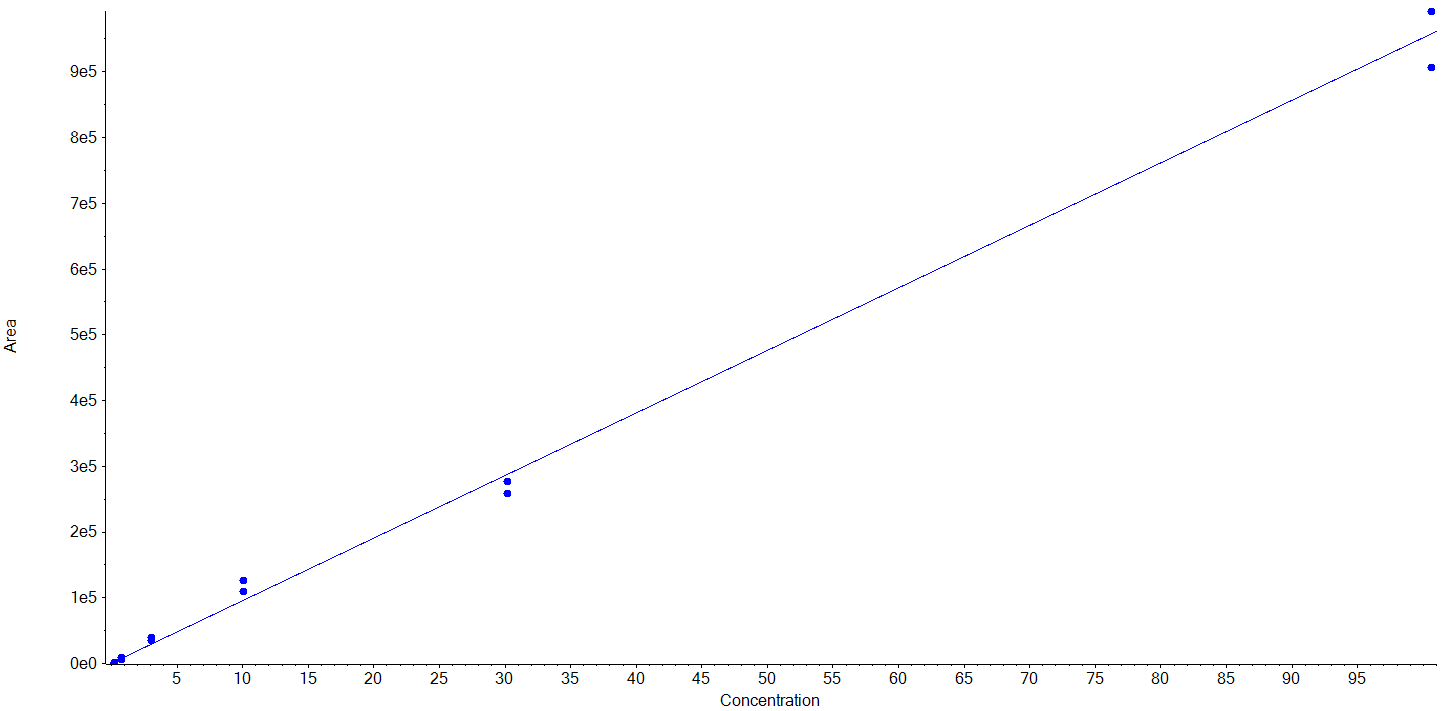 | Cyanidin-3-O-sambubioside 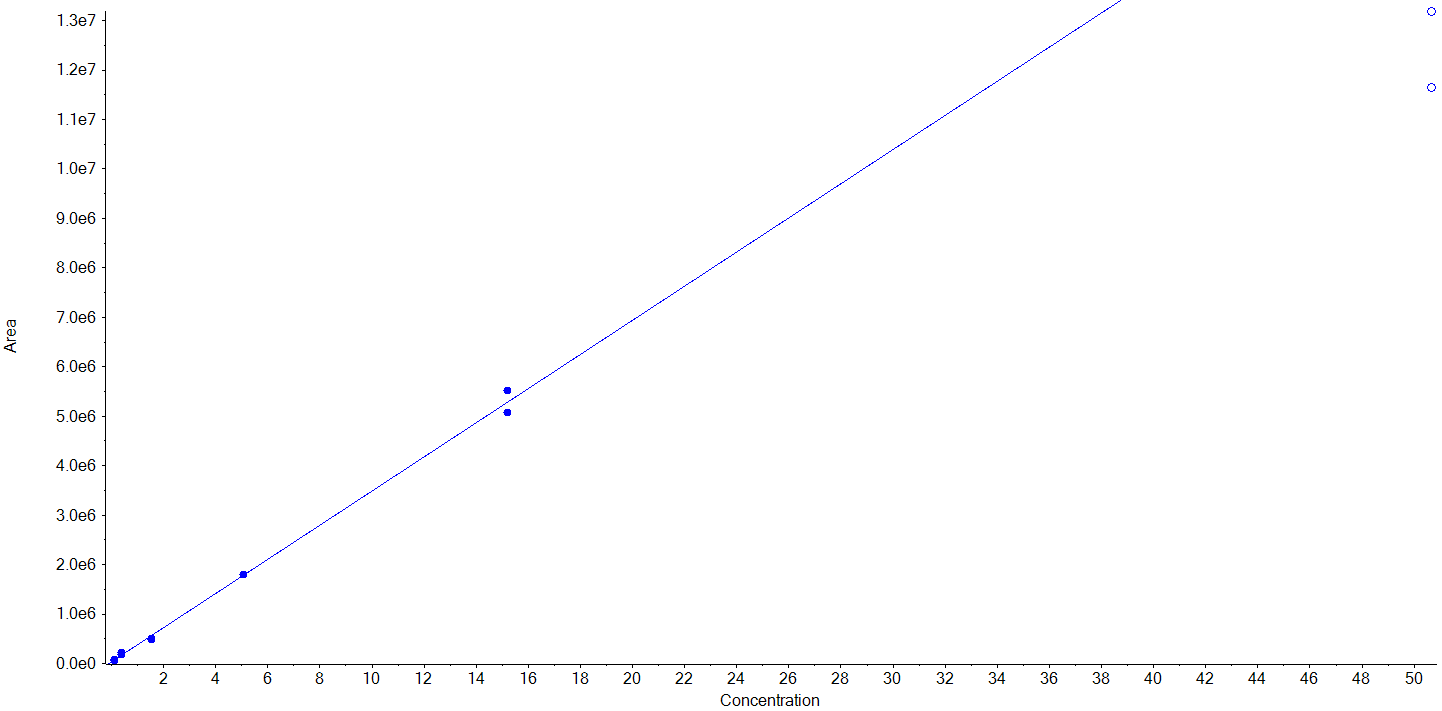 |
| Cyanidin-3-O-rutinoside 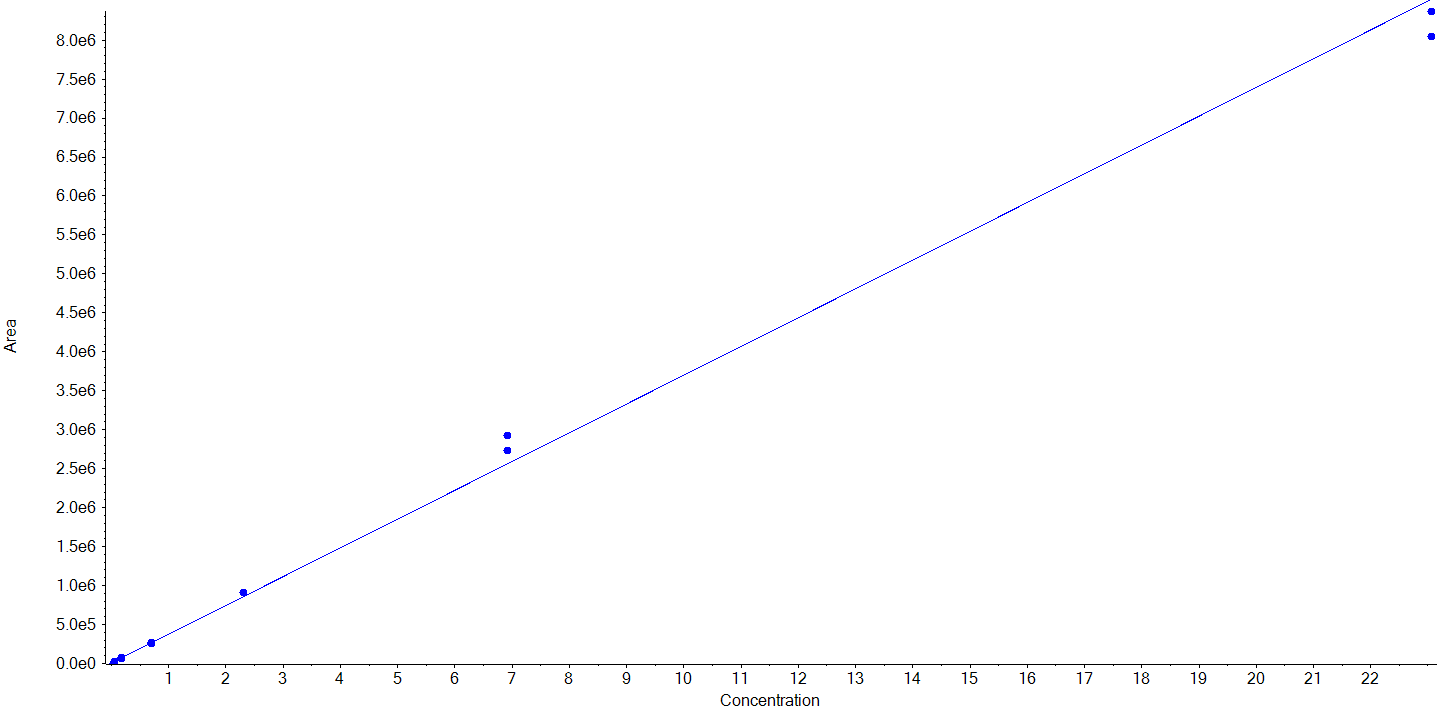 | Procyanidin C1 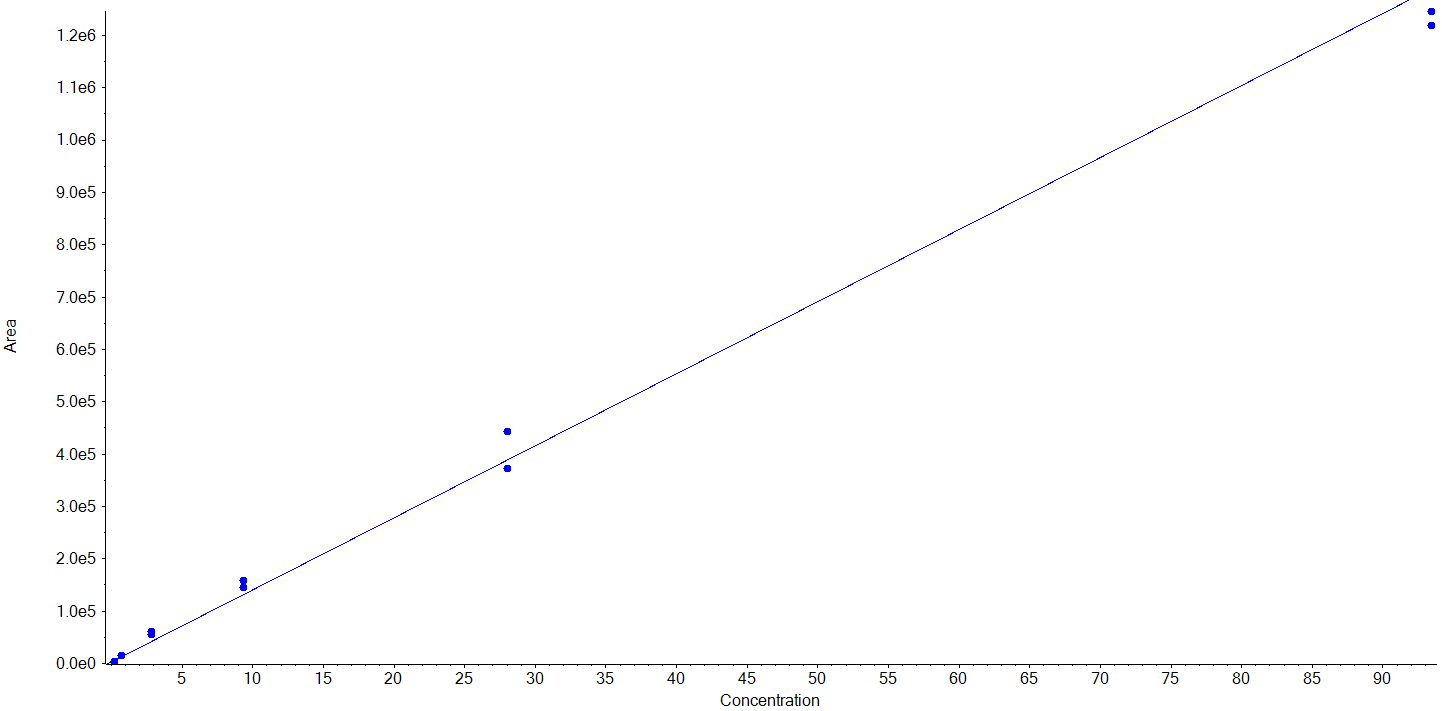 | Delphinidin 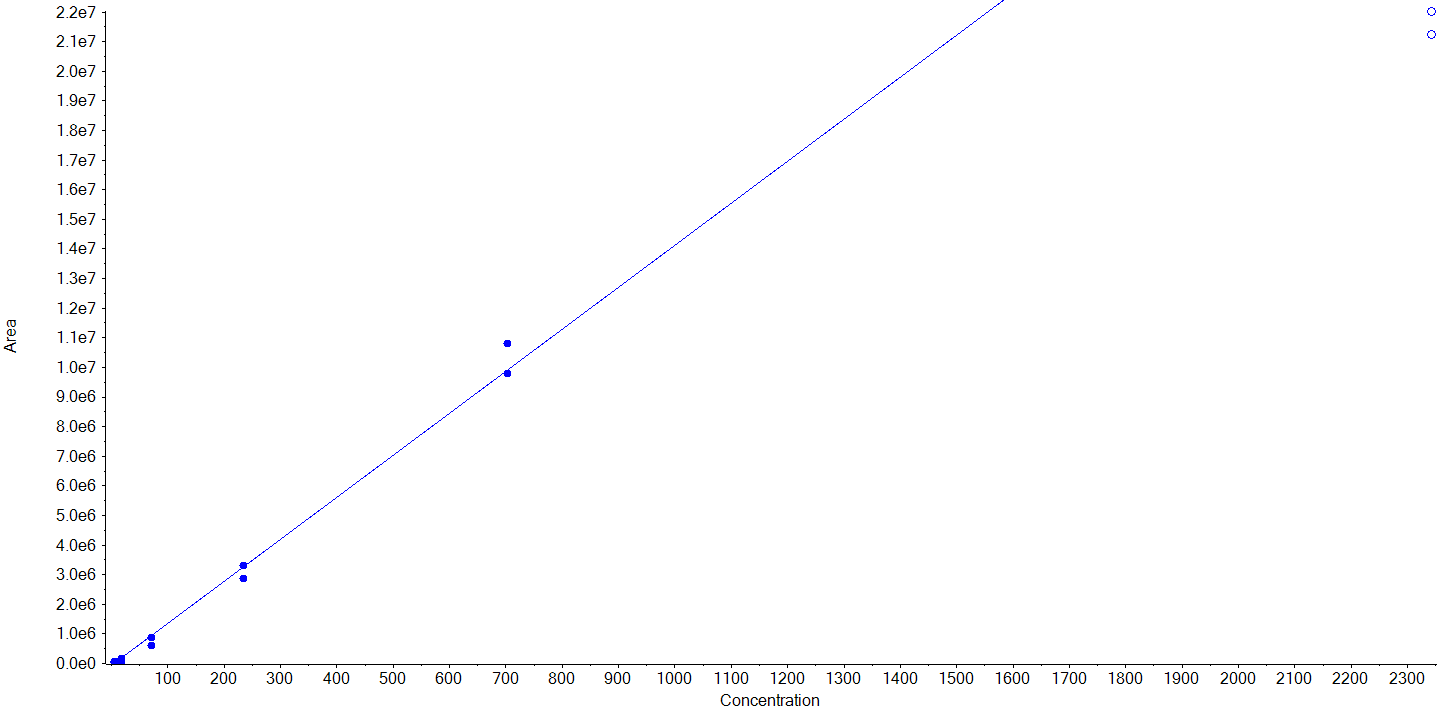 |
| Neohesperidin 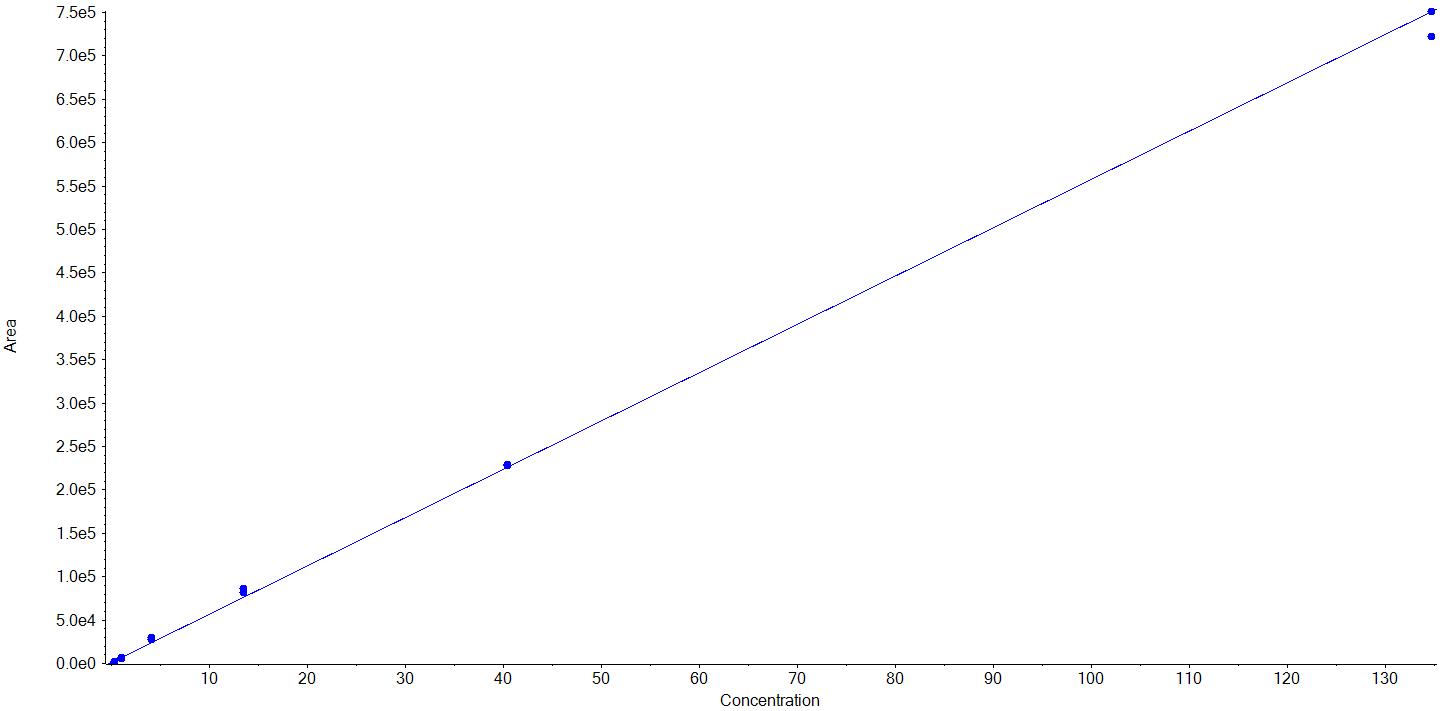 |  |  |

**References**

1. Oesterle I, Braun D, Rompel A, Warth B (2022) Quantifying up to 90 polyphenols simultaneously in human bio-fluids by LC-MS/MS. Anal Chim Acta 1216:339977. https://doi.org/10.1016/j.aca.2022.339977

1. No ion ratio could be determined since only one transition showed a chromatographic peak [↑](#footnote-ref-1)
2. Analyte with standard addition applied as a chromatographic peak was present in the matrix-matched blank. [↑](#footnote-ref-2)
